# Radical cascade cyclization of amino acid-tethered 1,6-enynones with sulfonyl hydrazides for N-terminal modification: synthesis of functionalized succinimide derivatives†

**DOI:** 10.1039/d5ra04754d

**Published:** 2025-08-01

**Authors:** Mathiyazhagan Sivanantham, Jenis Jacob Stanley, Kesavan Muthu, Sivan Velmathi, Gopal Chandru Senadi, Mohankumar Ramasamy

**Affiliations:** a Department of Chemistry, Faculty of Engineering and Technology, SRM Institute of Science and Technology SRM Nagar, Chengalpattu District Kattankulathur – 603 203 Tamil Nadu India chandrug@srmist.edu.in; b Interdisciplinary Institute of Indian System of Medicine (IIISM), SRM Institute of Science and Technology SRM Nagar, Chengalpattu District Kattankulathur – 603 203 Tamil Nadu India mohankur@srmist.edu.in; c Department of Chemistry, National Institute of Technology Tiruchirappalli – 620 015 Tamil Nadu India

## Abstract

A metal-free strategy for the N-terminal cyclization of amino acids has been developed by synthesizing highly functionalized succinimide derivatives through radical cyclization of amino acid-tethered 1,6-enynones with sulfonyl hydrazide using NIS and H_2_O_2_ as an oxidant. The notable advantages of this work includes time-efficient, good *E*/*Z* ratio, moderate to good yields, and was synthesized on a gram-scale. Furthermore, the synthetic utility of the product 5aa was performed by (i) Suzuki coupling reaction with iodo-functionality; and (ii) dipeptide formation using glycine methyl ester.

## Abstract 2

NIS/H_2_O_2_-mediated metal-free radical cascade cyclization of amino acid-tethered 1,6-enynones with sulfonyl hydrazide has been developed for selective N-terminal cyclization of amino acids by producing highly functionalized succinimide derivatives.

## Graphical abstract

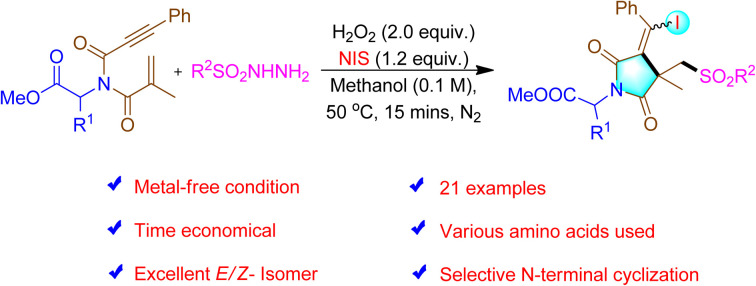

## 1 Introduction

Radical cascade cyclizations are an effective strategy for synthesizing complex organic skeletons, drug molecules, and functional materials^1*a*,*b*^ without the need for pre-functionalization or expensive transition metals.^1*c*^ Moreover, radical addition reactions have garnered significant attention for generating protein and peptide libraries with site-selective modifications and cyclizations.^2^ Among radical cascade cyclization's 1,6-enyne derivatives are particularly important substrates for preparing succinimides, an important N-containing five-membered heterocycle found in active pharmaceutical ingredients (APIs), biologically active natural compounds, and drug candidates.^3,4^ Likewise, sulfonyl-containing groups are highly significant in pharmaceutical, agricultural, and materials chemistry owing to their extensive biological activity and synthetic adaptability.^5^

**Scheme 1:**
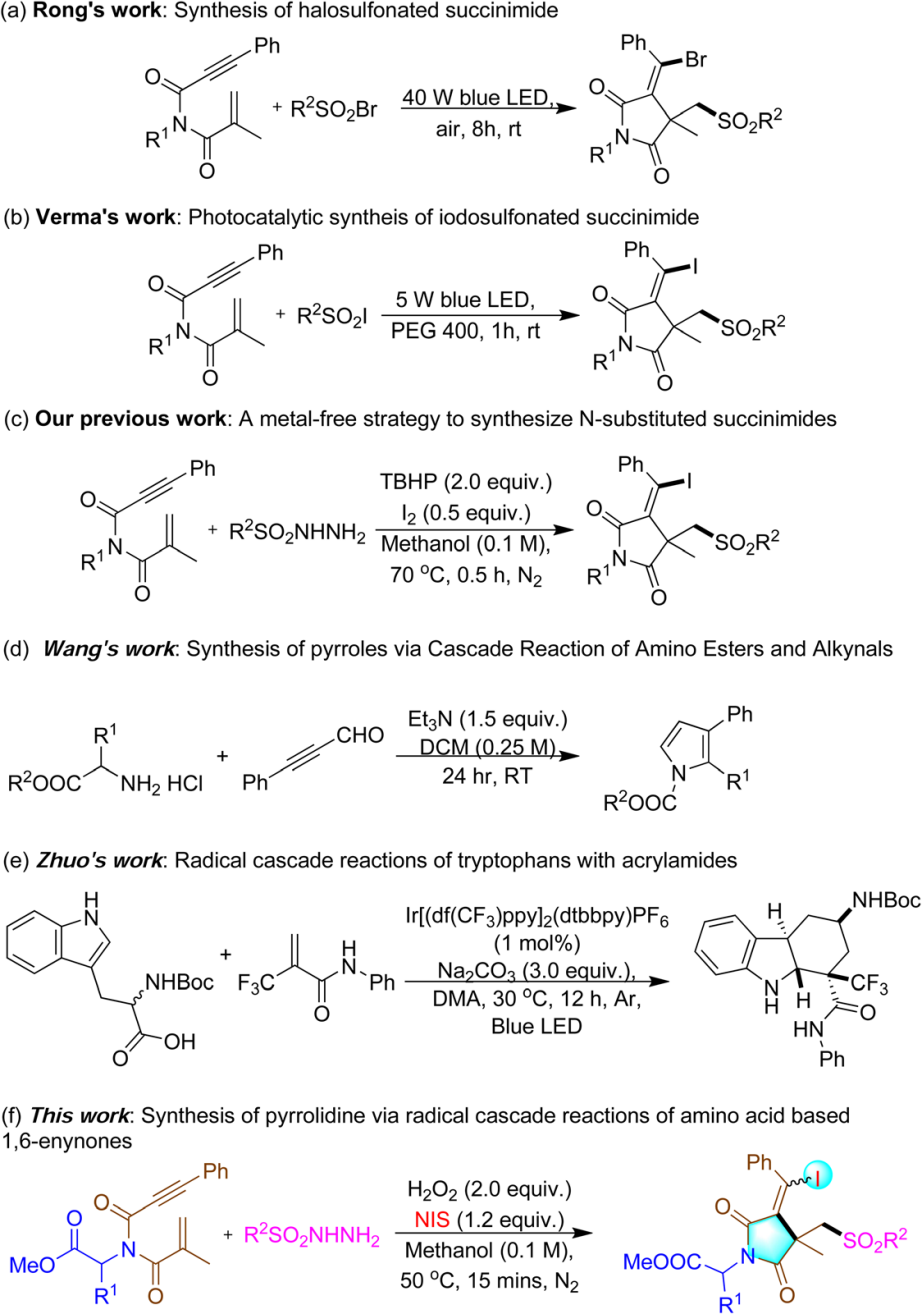
Previous and this study on synthesis of N-heterocyclic compounds.

Recently, numerous five-membered N-heterocycles have been synthesized *via* radical cascade cyclizations of aza-1,6-enynes employing diverse radical sources.^6*a*–*g*^ Among these, Rong *et al.* reported the difunctionalized succinimide derivatives in 2024 by employing sulfonyl bromides and 1,6-enynes (Scheme 1a).^6*h*^ Later, Verma *et al.* developed a photocatalytic approach in 2025 using sulfonyl iodides with 1,6-enynes to access similar succinimide frameworks (Scheme 1b).^6*i*^ Additionally, our previous work in 2022 demonstrated the synthesis of highly functionalized succinimide derivatives from aniline-based aza-1,6-enynones (Scheme 1c).^7^ So far, motivated by our prior research and other, we aimed to expand this concept to amino acid-tethered complexes, positing that these substrates could experience selective N-terminal cyclization under radical circumstances. Although the application of amino acid-tethered aza-1,6-enynones for selective N-terminal cyclization is mostly unexamined.

On the other hand, in nature fewer than twenty amino acids are used to construct the complex biomolecules found in living organisms.^8*a*^ Recently, there has been growing interest in the synthesis of unnatural amino acids due to their diverse applications in biotechnology, pharmaceuticals, biomolecules, and the total synthesis of natural products.^8*b*–*d*^ For example, they are used in medications such as antivirals and ACE inhibitors for treating renal and cardiovascular diseases.^9*a*,*b*^ Consequently, the synthesis of amino acids that are not readily available from natural sources requires the development of effective synthetic methods.^9*c*^

Furthermore, amino acid-tethered reactions often occur at the α-C(sp3)–H bond for synthetic modifications or involve utilizing both the N-terminus and α-carbon for N-heterocyclic syntheses.^10^ Additionally, in the last ten years, numerous N-heterocyclic structures have been synthesized from amino acids through cyclization reactions, encompassing pyridines,^11^ azetidinones,^12^ pyrazoles,^13^ thiazolidines,^14^ pyrrolidones,^15,16^ quinoline-fused lactones,^17^ dihydroquinolines,^18^ dihydropyridines,^19^ and proline-derived azabicycloalkanes.^20^ Moreover, N-terminal selective bioconjugation has garnered heightened interest owing to its prospective uses in chemical biology, proteomics, and peptide immobilization.^21^

For instance, Wang *et al.* (2020)^22^ synthesized pyrrole from readily available amino acid esters and propiolaldehydes using Et_3_N as a base (Scheme 1d). In 2022, Zhou *et al.*^23^ reported a stereoselective intermolecular cascade reaction to synthesize *trans*-fused hexahydrocarbazoles using tryptophan and acrylamide (Scheme 1e). However, selective N-terminal modifications have been less explored and remain an intriguing area of research.^24^ To the best of our knowledge, there are no reports instances of synthesizing iodosulfonylated succinimide derivatives *via* the radical cyclization of amino acid-tethered 1,6-enynones with sulfonyl hydrazide, without activating the α-carbon. This study introduces a unique method employing amino acid-derived 1,6-enynones for the selective N-terminal cyclization to create succinimide scaffolds. Herein, we report the synthesis of highly substituted succinimide derivatives from amino acid-tethered 1,6-enynones *via* a radical cascade cyclization reaction with H_2_O_2_ and NIS in methanol at 50 °C for 15 minutes, under a N_2_ atmosphere (Scheme 1f). This reaction proceeds through C–S, C–C, and C–I bond formation, yielding moderate to excellent results and achieving selective N-terminal cyclization.

## 2 Results and discussion

Initial studies began with methyl *N*-methacryloyl-*N*-(3-phenylpropioloyl)glycinate (3a) and 4-methylbenzene sulfonyl hydrazide (4a) as standard substrates using our previously reported reaction conditions.^7*a*^ The new stereogenic center product, 5aa, was obtained in 46% yield as a racemic mixture, and its structure was unambiguously confirmed by X-ray crystallography^7*b*^ alongside di-iodinated succinimide 6a as a by-product in 18% yield (Table 1, entry 1). Changing to other iodinating sources the yield of 5aa increased to 56% when NIS was used, while yields decreased with KI and TBAI (Table 1, entry 2–4). Varying the oxidants, revealed that H_2_O_2_ increased the yield of 5aa to 77% and reduced the by-product 6a to below 5% (Table 1, entries 5–7). Increasing the NIS equivalent to 1.2 equiv., boosted the yield of 5aa to 82%, with no significant improvement observed at higher NIS equivalents (Table 1, entry 8 and 9). At room temperature, the yield was dropped to 61% and a higher yield of 83% was achieved at 50 °C (Table 1, entry 10 and 11). No significant change was noticed in the yield of 5aa over varying reaction times, with an optimal reaction time of 15 min giving 84% yield (Table 1, entries 12 and 13). The product yield was dropped to 66% in open air and traces under O_2_ atmosphere (Table 1, entry 14 and 15). Thus, of the conditions screened, in Table 1, entry 12 (50 °C, 15 minutes, 1.2 equiv., of NIS, 2.0 equiv., of H_2_O_2_ in methanol) were selected as the standard conditions for further scope studies as presented in Table 2. Detailed optimization studies can be found in Tables S1–S5 in the ESI.† Additionally, we examined the substitution of NBS for NIS and TsCl for TsNHNH_2_ reactions. Nevertheless, the brominated succinimide derivative and compound 5aa were not acquired under these conditions.

**Table 1:** Optimization of reaction conditions^a^^,^^b^

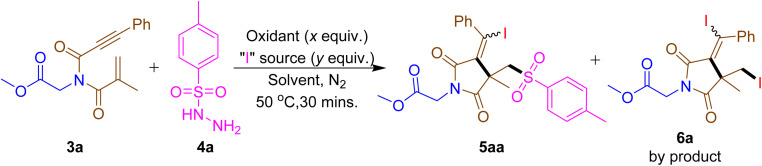						
Entry	Oxidant (*x* equiv.)	“I” source (*y* equiv.)	Solvent	Temp ^o^C	Yield^b^ (%)	
					5aa	6a
1	TBHP (2.0)	I_2_ (0.5)	MeOH	70	46	18
2	TBHP (2.0)	NIS (1.0)	MeOH	70	56	16
3	TBHP (2.0)	KI (1.0)	MeOH	70	30	17
4	TBHP (2.0)	TBAI (1.0)	MeOH	70	40	20
5	H_2_O_2_ (2.0)	NIS (1.0)	MeOH	70	77	<5
6	DTBP (2.0)	NIS (1.0)	MeOH	70	40	16
7	PIDA (2.0)	NIS (1.0)	MeOH	70	41	15
8	H_2_O_2_ (2.0)	NIS (1.2)	MeOH	70	82	<5
9	H_2_O_2_ (2.0)	NIS (1.5)	MeOH	70	78	<5
10	H_2_O_2_ (2.0)	NIS (1.2)	MeOH	RT	61	Trace
11	H_2_O_2_ (2.0)	NIS (1.2)	MeOH	50	83	Trace
12^c^	H_2_O_2_ (2.0)	NIS (1.2)	MeOH	50	84	Trace
13^d^	H_2_O_2_ (2.0)	NIS (1.2)	MeOH	50	83	Trace
14^e^	H_2_O_2_ (2.0)	NIS (1.2)	MeOH	50	56	24
15^f^	H_2_O_2_ (2.0)	NIS (1.2)	MeOH	50	Trace	38

a Reaction conditions: 3a (0.30 mmol), 4a (0.60 mmol), oxidant (*x* equiv.), iodo source (*y* equiv.) and solvent (0.1 M) at 50 °C for 15 min under N_2_ atmosphere unless otherwise noted.

b Isolated yield. H_2_O_2_ refers to 30% in an aqueous solution.

c Reaction time 15 min.

d Reaction time 1 h.

e Under air atmosphere.

f Under oxygen atmosphere.

**Table 2:** Scope of the amino acid-tethered 1,6-enynones with sulfonyl hydrazides^a^^,^^b^

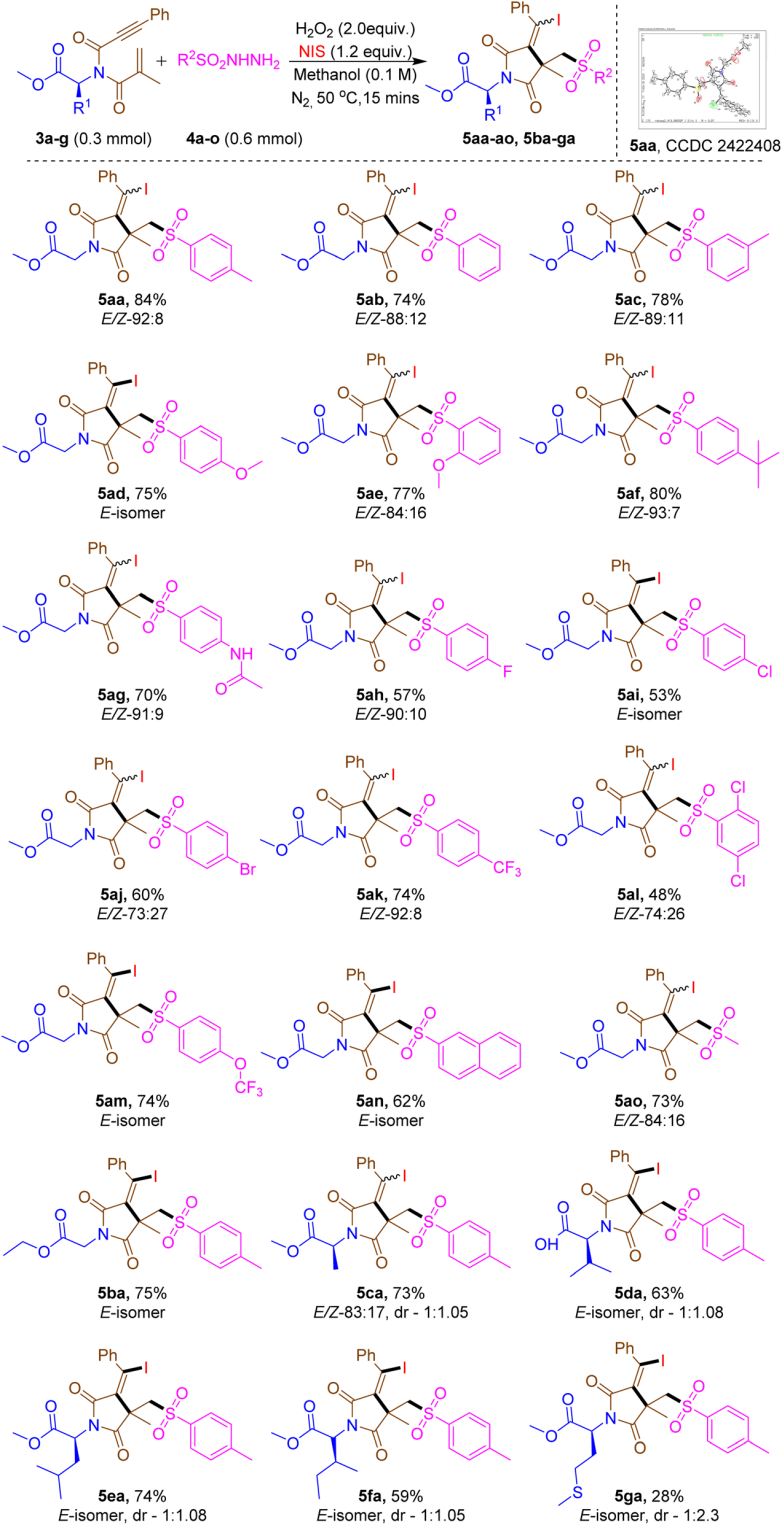

a Reaction conditions: 3a–g (0.3 mmol), 4a–o (0.6 mmol), H_2_O_2_ (30% in Aq.) (0.6 mmol), NIS (0.36 mmol) and MeOH (0.1 M) at 50 °C for 15 min under N_2_ atmosphere.

b Yield isolated. *E*/*Z* and dr ratio was calculated from ^1^H-NMR.

The reaction of methyl *N*-methacryloyl-*N*-(3-phenylpropioloyl)glycinate (3a) and sulfonyl hydrazide derivatives (4a–o) were investigated to deliver moderate to good yields of the compound 5 with excellent *E*/*Z*-ratio. The reaction was effective with benzene sulfonyl hydrazide (4b) and various electron-donating groups, including *m*-Me-Ph– (4c), *p*-MeO-Ph– (4d), *o*-MeO-Ph– (4e), *p-t*-Bu-Ph– (4f), and *p*-NH-COCH_3_-Ph (4g), producing the corresponding succinimide derivatives 5ab–ag in 70–80% yields. Furthermore, the electron-withdrawing substituents, such as *p*-F-Ph– (4h), *p*-Cl-Ph– (4i), *p*-Br-Ph– (4j), *p*-CF_3_-Ph– (4k), 2,5-di-Cl-Ph– (4l), and *p*-OCF_3_-Ph– (4m), exhibited a seamless reaction, yielding the expected products 5ah–am with yields between 48–74%. The viability of the work was assessed by the investigation of fused-ring (4n) and alkyl (4o) substituents of sulfonyl hydrazides. It is noteworthy that the reaction yielded the expected succinimide compounds 5an in 62% and 5ao in 73% yield. According to computational studies^25^ the major stereoselective *E*-isomer could originate due to the nonbonding/steric repulsion between the substituent groups on the quaternary carbon atom and bulky phenyl group attached to the double bond. Next, the scope of the amino acid was examined with various amino acid-tethered 1,6-enynones, as indicated in Table 2. The reaction worked well with the ethyl *N*-methacryloyl-*N*-(3-phenylpropioloyl)glycinate (3b) resulting in the corresponding succinimide derivative 5ba with a yield of 75%. Other aliphatic amino acid-tethered 1,6-enynones, such as alanine (3c), valine (3d), leucine (3e), and isoleucine (3f) also proceeded well and yielded the appropriate succinimide derivatives (5ca–fa) in 59–74% of the yields. In addition, methionine (3g), a sulfur containing amino acid, produced the desired product 5ga albeit in low yield.

In this study, amino acid-tethered 1,6-enynones were synthesized using l-amino acids as precursors. Afterthat, in the synthesis of the succinimide core introduced an additional chiral center, resulting in the detection of a racemic mixture of diastereomers. This may be due to the absence of a chiral catalyst or reagent in the synthesis of succinimide, therefore stereoselectivity was unregulated, which may result in the generation of diastereomeric molecules.

So far we assumed that, this mixture might be the result of the amino acid's α-carbon maintaining its (*S*)-configuration while the newly generated chiral center displays both (*S*)- and (*R*)-configurations. The diastereomeric ratio (dr) of the developed compounds 5ca–ga was determined from NMR data and is presented in Table 2.

Further, to expand the scope synthesis of di-iodinated succinimide derivatives (6) by utilizing the standard conditions Zhang *et al.*^6*c*^ was investigated as presented in Table 3. Interestingly, compound 3a reacted with I_2_ in ACN at room temperature for 30 min, resulting in 77% of the intended product 6a with a *Z*/*E* ratio of 66 : 34. Extending to various amino acid-tethered 1,6-enynones, such as ethyl glycine (3b), alanine (3c), valine (3d), and leucine (3e) also ended up in providing products 6b–e, with a yield range of 59–70%. The diastereomeric ratio (dr) of the developed compounds 6c–e was determined from NMR data and is presented in Table 3.

**Table 3:** Di-iodinated succinimide synthesis^a^^,^^b^

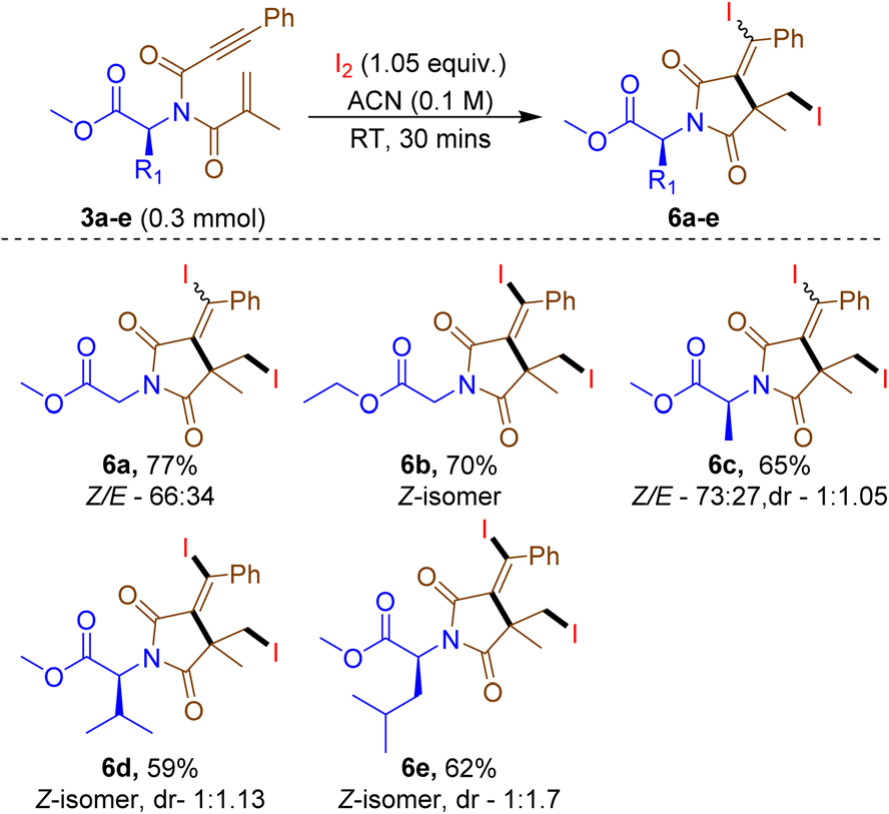

a Reaction conditions: 3a–e (0.3 mmol), I_2_ (0.315 mmol) and ACN (0.1 M) at room temperature for 30 min.

b Yield isolated. *Z*/*E* and dr ratio was calculated from ^1^H-NMR.

**Scheme 2:**
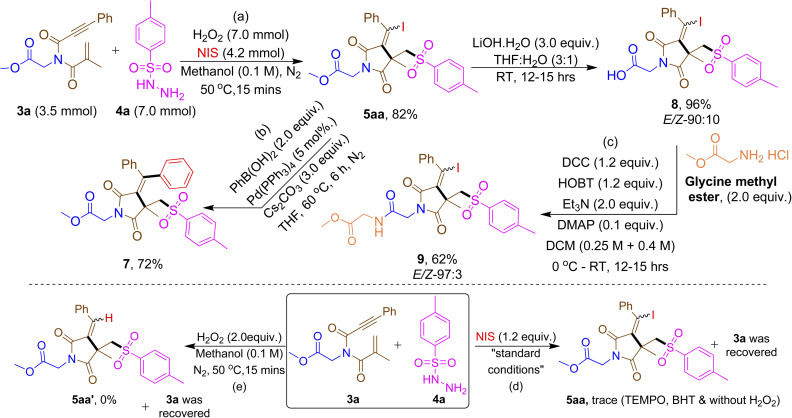
Gram-scale, synthetic application and control studies.

The scalability of the reaction was proven on a gram-scale synthesis (Scheme 2a) and the synthetic utility of the product was demonstrated using 5aa for (i) Suzuki coupling reaction with iodo-functionality (Scheme 2b); and (ii) dipeptide formation using glycine methyl ester (Scheme 2c). To elucidate the reaction mechanism, few control studies were performed. Radical scavenging studies with TEMPO and BHT failed to produce the desired product under standard conditions suggesting that the reaction may proceed *via* a radical pathway (Scheme 2d). The reaction did not proceed neither with NIS nor with H_2_O_2_ indicating both the reagents are necessary for the product formation (Scheme 2d and e).

**Scheme 3:**
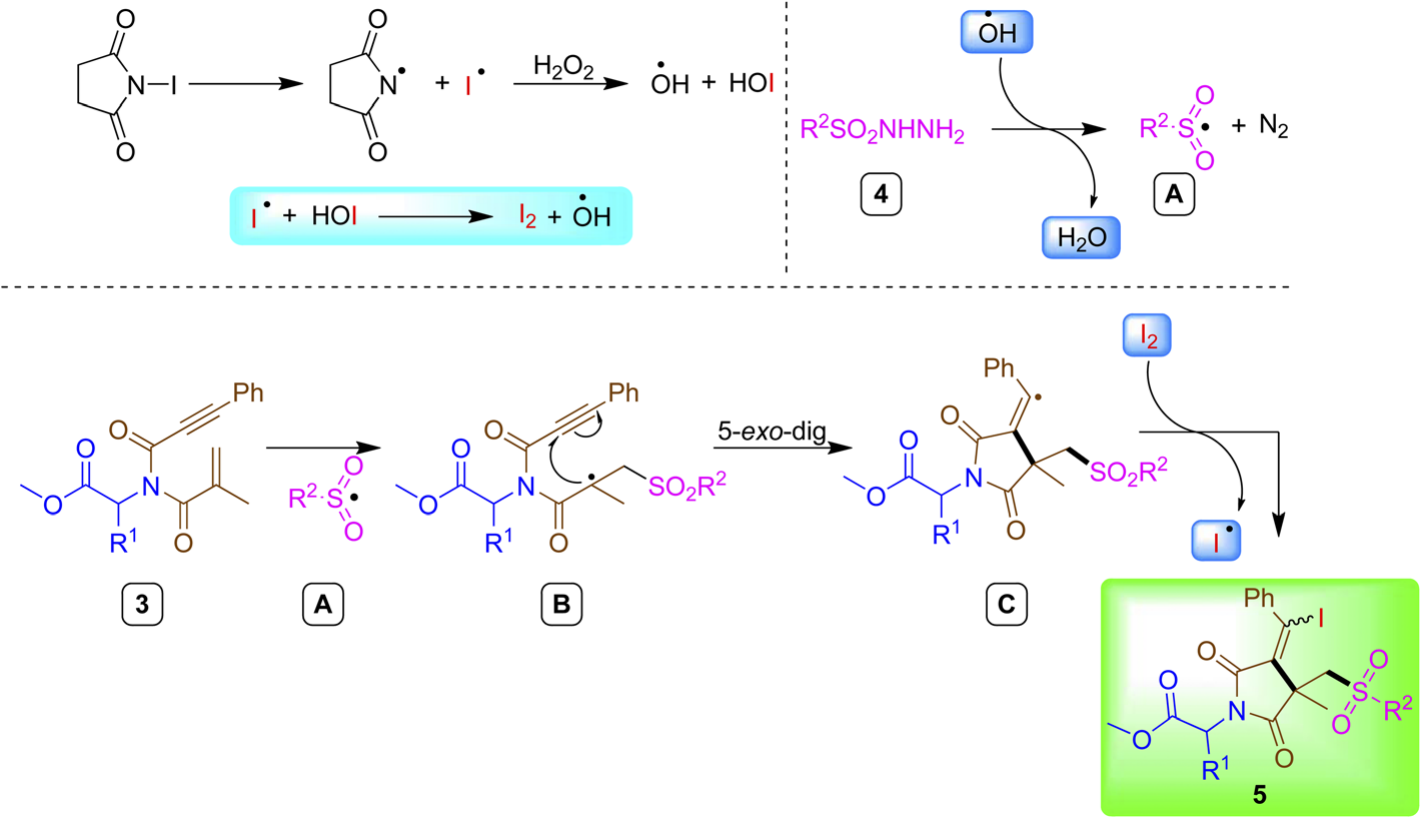
Plausible reaction mechanism.

Based on previous reports^26^ and control studies, a possible reaction mechanism was proposed for the synthesis of iodosulfonated succinimide derivatives (Scheme 3). The hydroxy radical generated from NIS/H_2_O_2_ reacted with sulfonyl hydrazides 4 to afford sulfonyl radical A. Then, the radical intermediate A was added to the amino acid-tethered 1,6-enynones 3 resulting in tertiary alkyl radical B. Next, intermediate B underwent intramolecular 5-*exo-dig* cyclization to produce *exo*-vinyl radical intermediate C. Finally, the alkenyl radical C was trapped by iodine to beget the final product 5 and the liberated iodo radical was oxidized *in situ* for the next catalytic cycle.

## 3 Conclusion

We present a simple, metal-free method for selective N-terminal cyclization of amino acid-tethered 1,6-enynones, producing highly functionalized succinimide derivatives. This process achieves moderate to excellent yields, excellent *E*/*Z* ratios, and gram-scale synthesis without the need for α-C(sp3)–H activation. Additionally, di-iodinated succinimides were synthesized with I_2_. The synthetic utility was further demonstrated through Suzuki coupling and dipeptide formation with glycine methyl ester, highlighting the method's versatility and efficiency.

## Author contributions

Mathiyazhagan Sivanantham – conceptualization, investigation, methodology, data curation, writing – review & editing; Jenis Jacob Stanley – methodology, data curation; Kesavan Muthu – data curation, formal analysis, resources; Sivan Velmathi – data curation, formal analysis, resources; Gopal Chandru Senadi – administration, supervision, data curation, writing – original draft; Mohankumar Ramasamy – conceptualization, methodology, project administration, supervision, data curation, writing – original draft.

## Conflicts of interest

There are no conflicts to declare.

## Supplementary Material

RA-015-D5RA04754D-s001

RA-015-D5RA04754D-s002

## Data Availability

The data supporting this article have been included as part of the ESI.†
