# Supplementary material for: Radical cascade cyclization of amino acid-tethered 1,6-enynones with sulfonyl hydrazides for N-terminal modification: synthesis of functionalized succinimide derivatives
Source: RSC Adv. 2025 Aug 1;15(34):27486–92. doi: 10.1039/d5ra04754d (PMC12315100; doi:10.1039/d5ra04754d)
Supplement: RA-015-D5RA04754D-s001 [file RA-015-D5RA04754D-s001.pdf]

Supplementary Information (SI) for RSC Advances.
This journal is © The Royal Society of Chemistry 2025

Supporting Information
for

Radical Cascade Cyclization of Amino Acid-Tethered 1,6-Enynones with Sulphonyl Hydrazides for N-Terminal Modification: Synthesis of Functionalized Succinimide Derivatives

Mathiyazhagan Sivanantham,[a] Jenis Jacob Stanley,[a] Kesavan Muthu,[b] Sivan Velmathi,[c] Gopal Chandru Senadi*[a] and Mohankumar Ramasamy*[a,b]

[a] Department of Chemistry, Faculty of Engineering and Technology, SRM Institute of Science and Technology, SRM Nagar, Kattankulathur - 603 203, Chengalpattu District, Tamil Nadu.

[b] Interdisciplinary Institute of Indian System of Medicine (IIISM), SRM Institute of Science and Technology, SRM Nagar, Kattankulathur - 603 203, Chengalpattu District, Tamil Nadu.

[c] Department of Chemistry, National Institute of Technology, Tiruchirappalli – 620 015, Tamil Nadu.

Email: mohankur@srmist.edu.in; chandrug@srmist.edu.in

Table of Contents

1	General Information	S3
2	Optimization Studies	S3-S7
3	Synthesis of Amino acid based 1,6-enynones (**3**)	S7-S8
4	General procedure for the synthesis of **5aa-ao** and **5ba-ga**	S8
5	General procedure for the synthesis of **6a-e**	S9
6	Control studies	S9-S10
7	Gram scale synthesis and Synthetic applications	S10-S12
8	Characterization Data of the Products **5aa-ao, 5ba-ga** and **6a-e**	S12-S22
9	References	S22
10	Scanned copies of ^{1}H and ^{13}C NMR Spectra **5aa-ao, 5ba-ga** and **6a-e**	S23-S80
11	X-Ray Crystallographic Data of **5aa**	S81-S82

(1) General Information

^{1}H NMR was recorded on a Bruker (500 MHz and 400 MHz), and ^{13}C NMR spectra were recorded on a Bruker (125 MHz/101 MHz). The chemical shift (δ) values are given in parts per million (ppm), and the coupling constants (J) are given in hertz (Hz). The spectra were recorded using $CDCl_3$ and DMSO-d_6 solvents. ^{1}H NMR chemical shifts are referenced to tetramethylsilane (TMS) (0 ppm) and ^{13}C NMR referenced to $CDCl_3$ (77.0 ppm) or DMSO-d_6 (39.51 ppm). HRMS recorded with Agilent 6540 Q-TOF. Melting point of compounds was determined on digital melting point apparatus (Model 33/0112) from a VEEGO-VMP-DS spectrometer. X-ray diffractions were recorded on a Bruker AXS GmbH, Germany (Model - Bruker D8 QUEST). The progress of the reaction was monitored by TLC using Merck pre-coated TLC sheets. Column chromatography was performed on 100–120 mesh silica gel using hexane/ethyl acetate as eluting solvents and solvents were used without further distillation, methanol is dried over 5A molecular sieves. All commercial chemicals were purchased from Merck, Avra, Carbanio, BLD and SRL. Sulphonyl hydrazides were prepared according to the previous literature methods[1] and used for the final reactions.

(2) Optimization studies

Initially, we started our research with methyl N-methacryloyl-N-(3 phenylpropioloyl)glycinate (**3a**) and 4-methyl benzene sulphonyl hydrazide (**4a**) using *tert*-butyl hydroperoxide (TBHP, 70% in Aq.) as an oxidant and iodine (I_2) as the iodo source in methanol as a solvent under N_2 atmosphere for 30 mins. Surprisingly, the new stereo genic center molecule **5aa** was found to be 46% of the yield. It was separated as a racemic mixture, and its structure was confirmed unambiguously by single-crystal X-ray diffraction data. Although we observed the di-iodinated succinimide compound **6a** as a byproduct with 18% yield (Table S1, entry 1). When screening other solvents (Table S1, entries 2-8), such as ethanol, toluene, acetonitrile (ACN), and dichloromethane (DCM), chloroform ($CHCl_3$), dichloroethane (DCE), tetrahydrofuran (THF), it showed methanol was the best solvent to afford the desired compound **5aa**.

Table S1 Screening of Solvents[a]

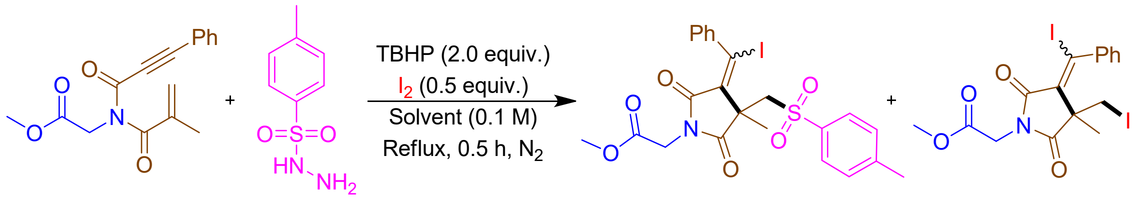

S. No	TBHP (equiv.)	I_2 (equiv.)	Solvent (0.1 M)	Temp (°C)	Yield (%)[b] 5aa	6a
1	2.0	0.5	Methanol	70	46	18
2	2.0	0.5	Ethanol	80	43	15
3	2.0	0.5	Toluene	90	42	17
4	2.0	0.5	CAN	80	40	21
5	2.0	0.5	DCM	50	42	22
6	2.0	0.5	$CHCl_3$	60	34	17
7	2.0	0.5	DCE	80	41	24
8	2.0	0.5	THF	70	39	19

Reaction conditions: [a] 0.30 mmol of **3a**, 0.6 mmol of **4a**, 2.0 equiv. of TBHP, 0.5 equiv. of I_2 and Solvent (0.1 M) at reflux for 0.5 h under N_2 atmosphere unless otherwise noted. [b] Isolated yield. TBHP refers to 70% in an aqueous solution.

By varying the iodo sources, lower in yield was observed while using KI and TBAI. However, the yield of compound **5aa** was increased up to 56% when NIS was employed as an iodo source (Table S2, entries 1-3).

Table S2 Screening of Iodo source[a]

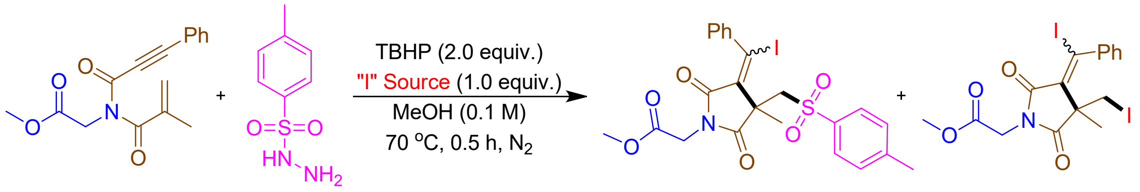

S. No	TBHP (equiv.)	"I" Source (1.0 equiv.)	Methanol (M)	Temp (°C)	Yield (%)[b]

					5aa	6a
1	2.0	NIS	0.1	70	56	16
2	2.0	KI	0.1	70	30	17
3	2.0	TBAI	0.1	70	40	20

Reaction conditions: [a] 0.30 mmol of **3a**, 0.6 mmol of **4a**, 2.0 equiv. of TBHP, 1.0 equiv. of "I" Source and MeOH (0.1 M) at 70 °C for 0.5 h under N_2 atmosphere unless otherwise noted. [b] Isolated yield. TBHP refers to 70% in an aqueous solution.

Further, when the reaction was carried out with alternative oxidant sources, such as hydrogen peroxide (H_2O_2), di-*tert*-butyl peroxide (DTBP), iodobenzene diacetate (PIDA), azoisobutyronitrile (AIBN), and cumene hydroperoxide (CHP), the yield of the compound **5aa** was enhanced up to 77% when using H_2O_2 as an oxidant source and the byproduct **6a** yield was reduced to less than 5%. Other oxidant source resulted in lower in yield (Table S3, entries 1-5).

Table S3 Screening of Oxidant[a]

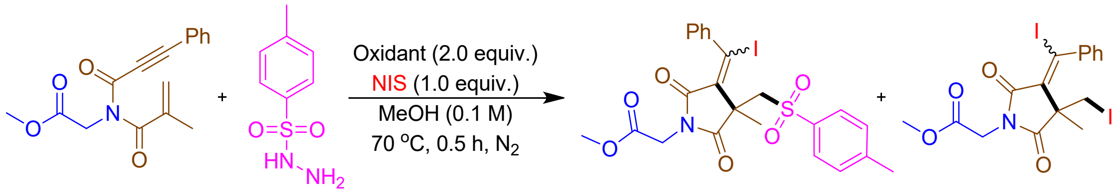

3a (0.3 mmol) **4a** (0.6 mmol) **5aa** **6a** by product

S. No	Oxidant (2.0 equiv.)	NIS (equiv.)	Methanol (M)	Temp (°C)	Yield (%)[b] 5aa	6a
1	H_2O_2	1.0	0.1	70	77	< 5
2	DTBP	1.0	0.1	70	40	16
3	PIDA	1.0	0.1	70	41	15
4	AIBN	1.0	0.1	70	44	18
5	CHP	1.0	0.1	70	46	17

Reaction conditions: [a] 0.30 mmol of **3a**, 0.6 mmol of **4a**, 2.0 equiv. of Oxidant, 1.0 equiv. of NIS and MeOH (0.1 M) at 70 °C for 0.5 h under N_2 atmosphere unless otherwise noted. H_2O_2 refers to 30% in an aqueous solution. [b] Isolated yield.

However, as shown in entries 1-2 in Table S4, changing the equivalents of H_2O_2 did not increase the product yield. On the other hand, increasing the NIS to 1.2 equiv. enhanced the yield of compound **5aa** by up to 82%, and further NIS increases did not increase the product's production (Table S4, entries 3-4). Nevertheless, altering the equivalents of **4a** did not enhance the product yield, as indicated in entries 5 and 6 in Table S4, resulting in a lower yield. Moreover, differing concentrations of methanol led to a decreased yield (Table S4, entries 7-8).

Table S4 Equivalent screening of Sulphonyl hydrazide, H_2O_2 and NIS[a]

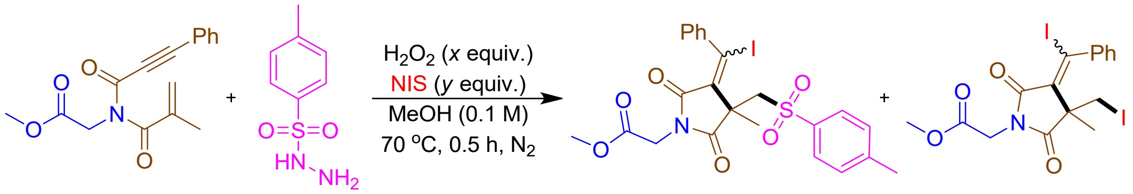

3a (0.3 mmol) **4a** (0.6 mmol) **5aa** **6a** by product

S. No	H_2O_2 (*x* equiv.)	NIS (*y* equiv.)	Methanol (M)	Temp (°C)	Yield (%)[b] 5aa	6a
1	3.0	1.0	0.1	70	69	< 5
2	1.5	1.0	0.1	70	65	< 5
3	2.0	1.2	0.1	70	82	Trace
4	2.0	1.5	0.1	70	78	Trace
5[c]	2.0	1.2	0.1	70	57	< 5
6[d]	2.0	1.2	0.1	70	60	< 5
7	2.0	1.2	0.05	70	52	< 5
8	2.0	1.2	0.2	70	66	< 5

Reaction conditions: [a] 0.30 mmol of **3a**, 0.6 mmol of **4a**, *x* equiv. of H_2O_2, *y* equiv. of NIS and MeOH (0.1 M) at 70 °C for 0.5 h under N_2 atmosphere unless otherwise noted. H_2O_2

refers to 30% in an aqueous solution. [b] Isolated yield. [c] 1.5 equiv. of sulphonyl hydrazide. [d] 3.0 equiv. of sulphonyl hydrazide.

Varying the reaction temperature gives the desired product **5aa** with 83% of the yield at 50 °C and other temperature conditions shows lower in yield (Table S5, entries 1-3). Furthermore, the yield of product **5aa** remained constant at both lower and higher reaction times, indicating that the reaction requires 15 minutes to produce the desired compound **5aa** with an 84% yield (Table S5, entries 4-5). The product's yield did not increase when the reaction was conducted in open air and argon atmosphere instead of N_2 atmosphere, resulting in a low product yield (Table S5, entries 6-7). However, under the oxygen atmosphere the product **5aa** was obtained traces only and it gives the byproduct **6a** with the yield of 38% (Table S5, entry 8). From the above optimization studies Table S5, entry 4 was chosen as the standard condition to obtain the desired product **5aa**.

Table S5 Screening of Temperature, Atmosphere, and Time[a]

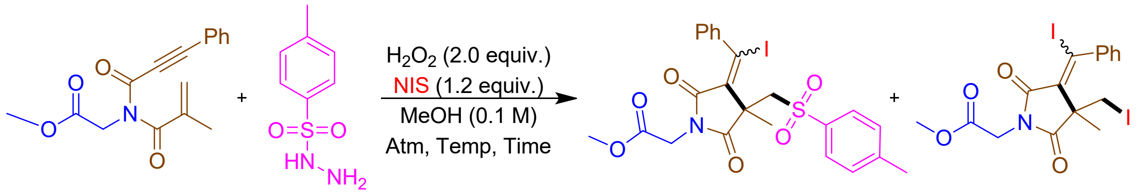

3a (0.3 mmol) **4a** (0.6 mmol) **5aa** **6a** by product

S. No	H_2O_2 (equiv.)	NIS (equiv.)	Methanol (M)	Temp (°C)	Yield (%)[b] 5aa	6a
1	2.0	1.2	0.1	RT	61	Trace
2	2.0	1.2	0.1	50	83	Trace
3	2.0	1.2	0.1	90	75	Trace
4[c]	2.0	1.2	0.1	50	84	Trace
5[d]	2.0	1.2	0.1	50	83	Trace
6[e]	2.0	1.2	0.1	50	56	24
7[f]	2.0	1.2	0.1	50	Trace	38

Reaction conditions: [a] 0.30 mmol of **3a**, 0.6 mmol of **4a**, 2.0 equiv. of H_2O_2, 1.2 equiv. of NIS and MeOH (0.1 M) at 50 °C for 15 mins under N_2 atmosphere unless otherwise noted.

H_2O_2 refers to 30% in an aqueous solution. [b] Isolated yield. [c] Reaction time 15mins. [d] Reaction time 1 hr. [e] Under open air atmosphere. [f] Under oxygen atmosphere.

(3) Synthesis of Amino acid based 1,6-enynones (3)[2]

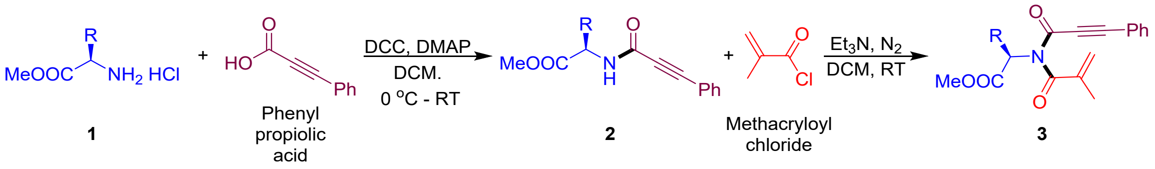

The methodology described in the literature[2] was used to prepare the derivatives of amino acid-based 1,6-enynones. To a dried round-bottom flask equipped with magnetic stir bar was charged with 2.0 mmol of phenyl propiolic acid, 0.2 mmol of DMAP and 5 mins N_2 purging. Then 0.25 M of DCM and 2.2 mmol of **1** were charged to the reaction mixture. The mixture was then cooled to 0 to 5 °C, followed by drop wise addition of 2.0 mmol of DCC dissolved in 0.4 M of DCM. Then the reaction mixture was warmed to RT and stirring for 12-15 h (monitored reactions by TLC). After the completion, the contents of the RBF was filtered through celite and washed with DCM. Then filtrate was diluted with H_2O (20 mL) and extracted with ethyl acetate (3×25 mL). The organic layers were dried with Na_2SO_4 and the solvent was then removed under reduced pressure with the aid of a rotary evaporator. The crude material was purified by silica gel column chromatography (Hex:EA = 7:3) to afford the corresponding product **2**.

To a dried round-bottom flask equipped with magnetic stir bar was charged with 1.0 mmol of **2** sealed with a septum and degassed by alternating vacuum evacuation and N_2 back filling. Then 1.5 mmol of methacryloyl chloride, 2.0 mmol of Et_3N and 0.2 M of DCM was charged under N_2 atmosphere. Then the reaction mixture was stirring for 6 h at room temperature (monitored reactions by TLC). After completion, the reaction mixture was diluted with H_2O (20 mL) and extracted with ethyl acetate (3×25 mL). The organic layers were dried with Na_2SO_4 and the solvent was then removed under reduced pressure with the aid of a rotary evaporator. The crude material was purified by silica gel column chromatography (Hex:EA = 9:1) to afford the corresponding product **3**.

(4) General procedure for the synthesis of 5aa-ao and **5ba-ga**

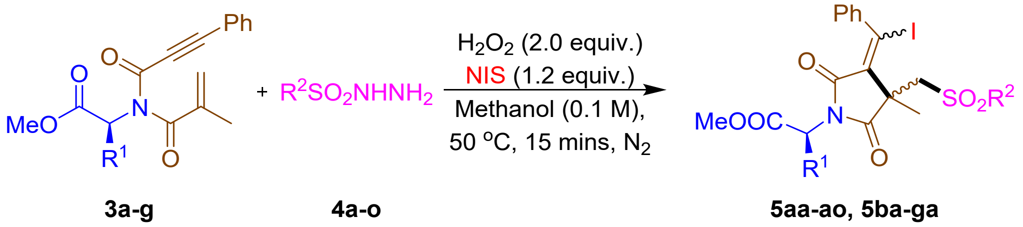

To a round-bottom flask equipped with magnetic stir bar was charged with **3a-g** (0.3 mmol) was dissolved with methanol (3 ml). Then **4a-o** (0.6 mmol), H_2O_2 (0.6 mmol) and NIS (0.36 mmol) was added to the reaction mixture, sealed with a septum, and degassed by alternating vacuum evacuation and N_2 back filling. Then the reaction mixture was heated to 50 °C with stirring for 15 mins (monitored reactions by TLC). After the completion, the mixture was quenched with saturated sodium thiosulphate solution (20 mL) and extracted with ethyl acetate (3×25 mL). The organic layers were dried with Na_2SO_4 and the solvent was then removed under reduced pressure with the aid of a rotary evaporator. The crude material was purified by silica gel column chromatography (Hex:EA = 7:3) to afford the corresponding product **5aa-ao** and **5ba-ga** as white solid.

(5) General procedure for the synthesis of 6a-e

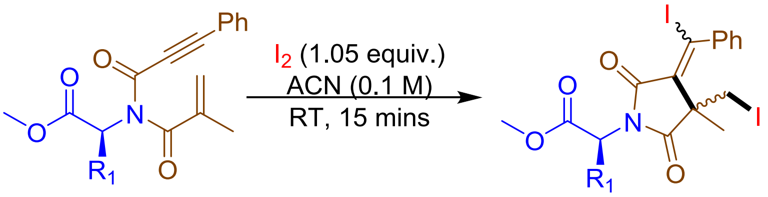

3a-e (0.3 mmol) **6a-e**

To a round-bottom flask equipped with magnetic stir bar was charged with **3a-e** (0.3 mmol) and I_2 (0.315 mmol) was dissolved into acetonitrile (0.1 M). Then the reaction mixture was stirring for 15 mins at air atmosphere under room temperature (monitored reactions by TLC). After the completion, the mixture was quenched with saturated sodium thiosulphate solution (20 mL) and extracted with ethyl acetate (3×25 mL). The organic layers were dried with Na_2SO_4 and the solvent was then removed under reduced pressure with the aid of a rotary evaporator. The crude material was purified by silica gel column chromatography (Hex:EA = 9:1) to afford the corresponding product **6a-e** as white solid.

(6) Control Studies

a) Without NIS or H_2O_2 Reaction.

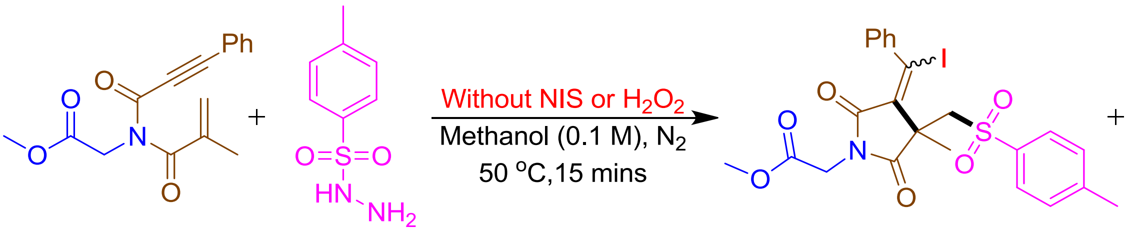

3a **4a** **5aa, 0%**

To a round-bottom flask equipped with magnetic stir bar was charged with **3a** (0.3 mmol) and **4a** (0.6 mmol) was dissolved in methanol (0.1 M). Then H_2O_2 (0.6 mmol) or NIS (0.36

mmol) was added to the reaction mixture, sealed with a septum, and degassed by alternating vacuum evacuation and N_2 back filling. Then the reaction mixture was heated to 50 °C with stirring for 15 mins (monitored reactions by TLC). **5aa** was not formed or traces observed, starting material remains not involved in the reaction and starting material was recovered.

b) TEMPO or BHT Reaction.

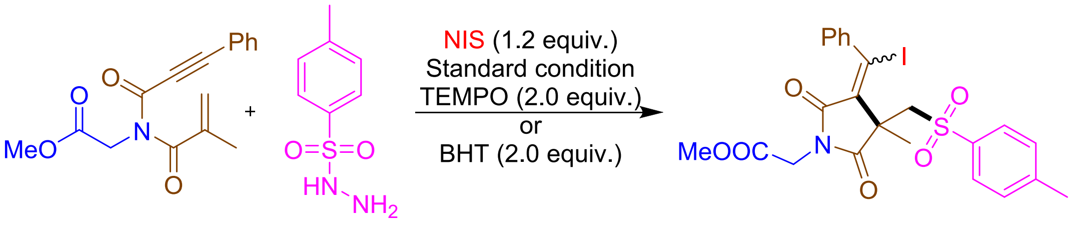

3a **4a** **5aa** (Traces)

To a round-bottom flask equipped with magnetic stir bar was charged with **3a** (0.3 mmol) was dissolved in methanol (0.1 M). Then **4a** (0.6 mmol), H_2O_2 (0.6 mmol), TEMPO or BHT (0.6 mmol) and NIS (0.36 mmol) was added to the reaction mixture, sealed with a septum, and degassed by alternating vacuum evacuation and N_2 back filling. Then the reaction mixture was heated to 50 °C with stirring for 15 mins (monitored reactions by TLC). **5aa** was not formed or traces observed, starting material remains not involved in the reaction and starting material was recovered.

(7) Gram Scale synthesis and Synthetic applications

(a) Experimental procedure for the gram scale synthesis

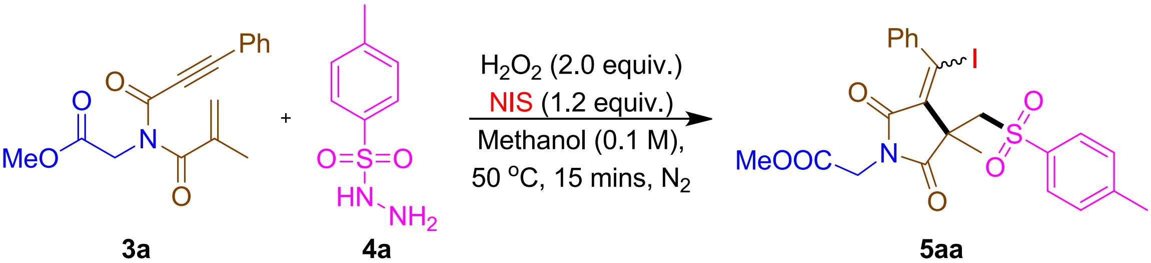

3a 1.0 g (3.5 mmol) **4a** 1.33 g (7.0 mmol) **5aa** 1.62 g (82% yield)

To a 100mL round-bottom flask equipped with magnetic stir bar was charged with **3a** (3.5 mmol) was dissolved with 3.5 ml of methanol. Then **4a** (7.0 mmol), H_2O_2 (7.0 mmol) and NIS (4.2 mmol) was added to the reaction mixture, sealed with a septum, and degassed by alternating vacuum evacuation and N_2 back filling. Then the reaction mixture was heated to 50 °C with stirring for 15 mins (monitored reactions by TLC). After the completion, the mixture was quenched with saturated sodium thiosulphate solution (100 mL) and extracted with ethyl acetate (3×50 mL). The organic layers were dried with Na_2SO_4 and the solvent was then removed under reduced pressure with the aid of a rotary evaporator. The crude material

was purified by silica gel column chromatography (Hex:EA = 7:3) to afford the corresponding product **5aa** as a white solid (1.62 g, yield = 82%).

(b) Synthesis of methyl 2-(4-(diphenylmethylene)-3-methyl-2,5-dioxo-3-(tosylmethyl) pyrrolidin-1-yl)acetate (7)

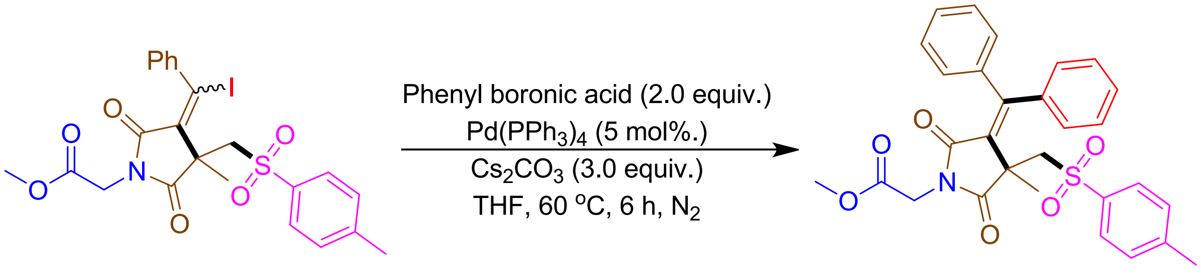

5aa **7**, 72%

To a round-bottom flask added **5aa** (0.2 mmol, 113 mg), phenyl boronic acid (0.4 mmol, 49 mg), $Pd(PPh_3)_4$ (5 mol %, 11.5 mg), Cs_2CO_3 (0.6 mmol, 212 mg) and degassed by alternating vacuum evacuation and N_2 back filling. Then, THF (2 mL) was added under N_2 atmosphere. Then the reaction mixture was heated to 60 ºC with stirring for 6 h (monitored reactions by TLC). The mixture was diluted with H_2O (20 mL) and extracted with ethyl acetate (3×25 mL). The organic layers were dried with Na_2SO_4 and the solvent was then removed under reduced pressure with the aid of a rotary evaporator. The crude material was purified by silica gel column chromatography (Hex:EA = 7:3) to afford the corresponding product **7** as white solid (74 mg, yield = 72%); mp: 188-191 ºC;^{1}H NMR (500 MHz, $CDCl_3$) δ 7.70 (d, *J* = 8.3 Hz, 2/04H), 7.48 (d, *J* = 1.5 Hz, 1.03H), 7.46 (s, 1.03H), 7.45 – 7.31 (m, 10.27H), 4.32 (dd, *J* = 40.4, 17.1 Hz, 2.09H), 3.72 (s, 3.06H), 3.48 (d, *J* = 14.2 Hz, 1.06H), 3.10 (d, *J* = 14.2 Hz, 1.02H), 2.43 (s, 3H), 1.42 (s, 2.92H); ^{13}C NMR (126 MHz, $CDCl_3$) δ 176.8, 167.2 , 166.9, 156.5, 144.9, 140.8, 139.1, 137.4, 129.7, 128.5, 128.3, 128.2, 128.1, 127.9, 127.5, 126.4, 59.9, 52.4, 45.9, 39.4, 26.8, 21.5; HRMS (ESI): Calc'd for $[M+H]^+$ $C_{29}H_{28}NO_6S$: 518.1637; found 518.1658.

(c) Synthesis of 2-(4-(iodo(phenyl)methylene)-3-methyl-2,5-dioxo-3-(tosylmethyl) pyrrolidin-1-yl)acetic acid (8)

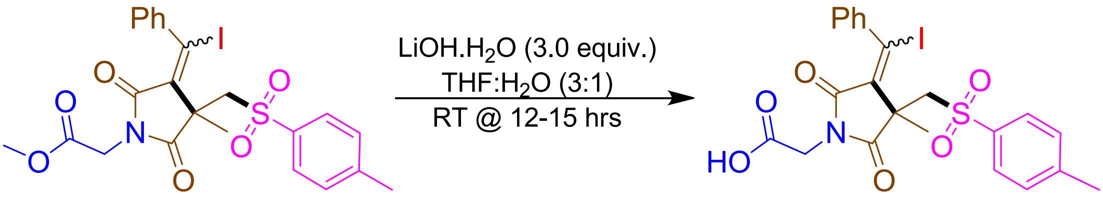

5aa **8**, 96%
E/*Z*-90:10

To a round-bottom flask added compound **5aa** (113 mg, 0.2 mmol,), LiOH.H_2O (25 mg, 0.6 mmol) and THF/H_2O (0.05 M - 3:1) were added and the reaction mixture stirring for 12-15 h at room temperature (monitored reactions by TLC). The mixture was diluted with H_2O (20 mL) and extracted with ethyl acetate (3×15 mL). The aqueous layer was then acidified with dilute HCl to get pH 1 to 2. Then the aqueous layer was extracted with ethyl acetate (3×25 mL). The organic layers were dried with Na_2SO_4 and the solvent was then removed under reduced pressure with the aid of a rotary evaporator to afford the corresponding product **8** as white solid (106 mg, yield = 96%, *E*/*Z* ratio 90:10); mp: 256-258.6 ºC; ^{1}H NMR (500 MHz, DMSO-d_6) δ 7.74 (d, *J* = 7.9 Hz, 1.92H), 7.66 (d, *J* = 7.8 Hz, 0.18H), 7.50 (d, *J* = 7.9Hz, 2.22H), 7.48 – 7.43 (m, 0.32H), 7.37 (t, *J* = 7.5 Hz, 2.11H), 7.29 (t, *J* = 7.3 Hz, 1.13H), 7.15 (d, *J* = 7.1 Hz, 1.86H), 4.60 (d, *J* = 15.1 Hz, 1H), 4.06 (q, *J* = 20.5 Hz, 2.19H), 3.85 (d, *J* = 15.1 Hz, 1.02H), 3.69 (d, *J* = 14.8 Hz, 0.13H), 2.95 (d, *J* = 15.0 Hz, 0.11H), 2.45 (s, 2.85H), 2.43 (s, 0.34H), 2.00 (s, 0.17H), 1.61 (s, 2.84H); ^{13}C NMR (126 MHz, DMSO-d_6) δ 176.2, 167.8, 163.4, 145.08, 145.04, 137.0, 133.6, 130.1, 128.5, 127.8, 127.5, 126.5, 118.9, 57.1, 47.4, 22.0, 21.1; HRMS (ESI): Calc'd for $[M+H]^+$ $C_{22}H_{21}INO_6S$: 554.0134; found 554.0146.

(d) Synthesis of methyl (2-(4-(iodo(phenyl)methylene)-3-methyl-2,5-dioxo-3-(tosyl methyl)pyrrolidin-1-yl)acetyl)glycinate (9)

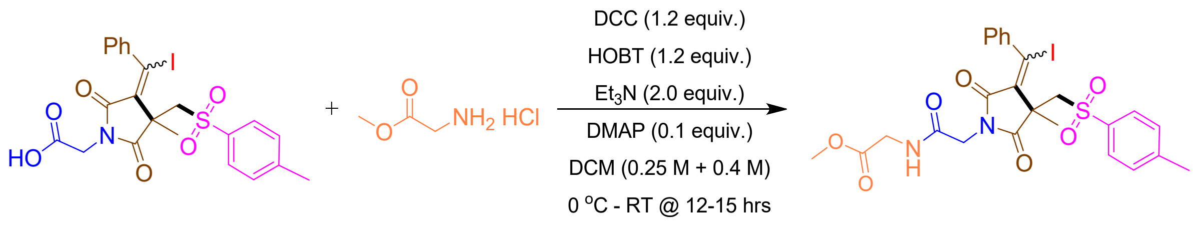

8 Glycine methyl ester **9**, 62% *E*/*Z*-97:3

To a round-bottom flask added **8** (0.2 mmol, 111 mg), Et_3N (0.4 mmol, 41 mg), DMAP (0.02 mmol, 2.5 mg) dissolve in DCM (0.25 M) and cold to 0-5 ºC. The added to the reaction mixture glycine methyl ester hydrochloride (0.4mmol, 50mg), DCC (0.24 mmol, 50mg) dissolved in 0.4 M of DCM, followed by HOBT (0.24 mmol, 32 mg). Then the reaction mixture was stirring for 12-15 hrs at room temperature (monitored reactions by TLC). The mixture was diluted with H_2O (20 mL) and extracted with ethyl acetate (3×25 mL). The organic layers were dried with Na_2SO_4 and the solvent was then removed under reduced pressure with the aid of a rotary evaporator. The crude material was purified by silica gel column chromatography (Hex:EA = 5:5) to afford the corresponding product **9** as white solid (78 mg, yield = 62%, *E*/*Z* ratio 97:3); mp: 194.8-197.2 ºC; ^{1}H NMR (500 MHz, $CDCl_3$) δ 7.84 (d, *J* = 8.2 Hz, 2.10H), 7.73 (d, *J* = 8.3 Hz, 0.07H), 7.45 – 7.39 (m, 4.37H), 7.39 – 7.33

(m, 3.12H), 7.16 (s, 0.98H), 4.63 (d, J = 14.4 Hz, 1.02H), 4.30 (q, J = 19.4 Hz, 2.16H), 4.12 – 3.88 (m, 2.22H), 3.76 (d, J = 14.3 Hz, 1.19H), 3.72 (s, 3.11H), 2.50 (s, 3H), 2.47 (s, 0.10H), 2.07 (s, 0.14H), 1.74 (s, 3.09H); ^{13}C NMR (126 MHz, $CDCl_3$) δ 176.9, 169.4, 165.8, 163.4, 145.4, 144.3, 136.7, 133.6, 130.0, 129.2, 127.9, 127.7, 126.6, 119.7, 57.6, 52.0, 48.1, 41.6, 40.9, 22.1, 21.6; HRMS (ESI): Calc'd for $[M+H]^+$ $C_{25}H_{26}IN_2O_7S$: 625.0505; found 625.0510.

(8) Characterization Data of the Products 4aa-al and 4ba-ma

Methyl 2-(4-(iodo(phenyl)methylene)-3-methyl-2,5-dioxo-3-(tosylmethyl)pyrrolidin-1-yl)acetate (5aa). The title compound was prepared according to the general procedure and purified by silica gel column chromatography (Hex:EA = 7:3) to afforded the desired product **5aa** as a white solid (144 mg, yield = 84%, *E/Z* ratio 92:8); mp:180-182.6 °C; ^{1}H NMR (500 MHz, $CDCl_3$) δ 7.79 (d, J = 8.3 Hz, 1.98H), 7.68 (d, J = 8.3 Hz, 0.17H), 7.41 – 7.31 (m, 7.48H), 4.59 (d, J = 14.3 Hz, 1H), 4.33 (d, J = 17.1 Hz, 0.17H), 4.27 (s, 1.96H), 3.79 (s, 0.23H), 3.71 (s, 2.99H), 3.68 (d, J = 14.4 Hz, 1.03H), 3.45 (d, J = 14.4 Hz, 0.09H), 3.10 (d, J = 14.5 Hz, 0.09H), 2.47 (s, 3H), 2.45 (s, 0.24H), 1.73 (s, 2.87H), 1.23 (s, 0.20H); ^{13}C NMR (101 MHz, $CDCl_3$) δ 176.8, 166.9, 163.4, 145.1, 144.4, 137.2, 133.9, 129.9, 129.1, 128.0, 127.8, 126.8, 118.9, 60.8, 57.4, 52.6, 48.1, 40.1, 39.4, 25.1, 22.1, 21.6; HRMS (ESI): Calc'd for $[M+H]^+$ $C_{23}H_{23}INO_6S$: 568.0291; found 568.0284.

Methyl 2-(4-(iodo(phenyl)methylene)-3-methyl-2,5-dioxo-3-((phenylsulfonyl)methyl) pyrrolidin-1-yl)acetate (5ab). The title compound was prepared according to the general procedure and purified by silica gel column chromatography (Hex:EA = 7:3) to afforded the desired product **5ab** as a white solid (124 mg, yield = 74%, *E/Z* ratio 88:12); mp:177-179.5 °C; ^{1}H NMR (500 MHz, $CDCl_3$) δ 7.92 (d, J = 7.2 Hz, 1.98H), 7.81 (d, J = 7.2 Hz, 0.27H), 7.71 – 7.66 (m, 1.12H), 7.59 (t, J = 7.8 Hz, 2.25H), 7.41 – 7.31 (m, 5.47H), 4.61 (d, J = 14.3 Hz, 1H), 4.28 (s, 1.90H), 3.79 (s, 0.42H), 3.75 – 3.69 (m, 4.07H), 3.48 (d, J = 14.5 Hz, 0.14H), 3.12 (d, J = 14.5 Hz, 0.13H), 1.74 (s, 2.91H), 1.25 (s, 0.39H); ^{13}C NMR (101 MHz, $CDCl_3$) δ 176.7, 175.0, 166.9, 163.4, 144.4, 140.1, 134.0, 133.9, 129.4, 129.2, 128.0, 127.8, 126.8, 119.0, 60.6, 57.4, 52.7, 52.6, 48.1, 47.2, 40.1, 39.4, 25.2, 22.2; HRMS (ESI): Calc'd for $[M+H]^+$ $C_{22}H_{21}INO_6S$: 554.0134; found 554.0141.

Methyl 2-(4-(iodo(phenyl)methylene)-3-methyl-2,5-dioxo-3-((*m*-tolylsulfonyl) methyl) pyrrolidin-1-yl)acetate (5ac). The title compound was prepared according to the general procedure and purified by silica gel column chromatography (Hex:EA = 7:3) to afforded the desired product **5ac** as a white solid (134 mg, yield = 78%, *E/Z* ratio 89:11); mp:175.5-178.9 °C; ^{1}H NMR (500 MHz, $CDCl_3$) δ 7.71 (s, 1.88H), 7.61 (s, 0.28H), 7.51 – 7.44 (m, 2.15H), 7.42 – 7.31 (m, 5.29H), 4.60 (d, J = 14.4 Hz, 1H), 4.28 (s, 1.88H), 3.79 (s, 0.43H), 3.73 –

3.67 (m, 3.99H), 3.47 (d, J = 14.5 Hz, 0.12H), 3.11 (d, J = 14.5 Hz, 0.13H), 2.46 (s, 0.38H), 2.45 (s, 2.92H), 1.74 (s, 2.84H), 1.24 (s, 0.46H); ^{13}C NMR (101 MHz, $CDCl_3$) δ 176.7 , 175.0, 167.0, 166.9, 163.4, 144.4, 139.9, 139.7, 134.8, 133.9, 129.27, 129.20, 128.0, 127.9, 126.7, 124.9, 119.0, 60.7, 57.3, 52.6, 48.0, 47.1, 40.1, 39.4, 25.1, 22.1, 21.2; HRMS (ESI): Calc'd for $[M+H]^+$ $C_{23}H_{23}INO_6S$: 568.0291; found 568.0299.

Methyl (*E*)-2-(4-(iodo(phenyl)methylene)-3-(((4-methoxyphenyl)sulfonyl)methyl)-3-methyl-2,5-dioxopyrrolidin-1-yl)acetate (5ad). The title compound was prepared according to the general procedure and purified by silica gel column chromatography (Hex:EA = 7:3) to afforded the desired product **5ad** as a white solid (132 mg, yield = 75%, *E*-isomer); mp:214-216.2 ^{o}C; ^{1}H NMR (500 MHz, $CDCl_3$) δ 7.83 (d, J = 8.9 Hz, 1.94H), 7.41 – 7.29 (m, 5.30H), 7.02 (d, J = 8.9 Hz, 2H), 4.58 (d, J = 14.4 Hz, 1H), 4.27 (s, 1.89H), 3.89 (s, 3.01H), 3.71 (s, 3.02H), 3.68 (d, J = 14.4 Hz, 1.14H), 1.73 (s, 2.84H); ^{13}C NMR (101 MHz, $CDCl_3$) δ 176.8, 166.9, 163.9, 163.5, 144.4, 133.9, 131.6, 130.0, 129.1, 127.9, 126.8, 118.8, 114.5, 57.6, 55.7, 52.5, 48.1, 39.4, 22.1; HRMS (ESI): Calc'd for $[M+H]^+$ $C_{23}H_{23}INO_7S$: 584.0240; found 584.0232.

Methyl 2-(4-(iodo(phenyl)methylene)-3-(((2-methoxyphenyl)sulfonyl)methyl)-3-methyl-2,5-dioxopyrrolidin-1-yl)acetate (5ae). The title compound was prepared according to the general procedure and purified by silica gel column chromatography (Hex:EA = 7:3) to afforded the desired product **5ae** as a white solid (136 mg, yield = 77%, *E/Z* ratio 84:16); mp:226-229 ^{o}C; ^{1}H NMR (500 MHz, $CDCl_3$) δ 7.85 (d, J = 7.8 Hz, 0.98H), 7.79 (d, J = 7.8 Hz, 0.20H), 7.63 (t, J = 7.9 Hz, 1.16H), 7.42 (t, J = 7.5 Hz, 0.52H), 7.36 (t, J = 7.4 Hz, 2.13H), 7.31 (d, J = 7.2 Hz, 0.99H), 7.29 (s, 0.25H), 7.25 (s, 1.99H), 7.13 – 7.04 (m, 2.30H), 7.01 (d, J = 8.4 Hz, 0.22H), 4.80 (d, J = 14.6 Hz, 1H), 4.16 (q, J = 18.0 Hz, 2.12H), 4.05 (s, 2.87H), 3.97 (d, J = 14.7 Hz, 1.07H), 3.95 (s, 0.53H), 3.83 (d, J = 14.7 Hz, 0.26H), 3.75 (s, 0.56H), 3.69 (s, 2.97H), 3.28 (d, J = 14.7 Hz, 0.20H), 1.77 (s, 3.01H), 1.23 (s, 0.57H); ^{13}C NMR (101 MHz, $CDCl_3$) δ 176.5, 166.9, 163.4, 157.7, 157.5, 144.5, 143.7, 136.0, 134.2, 130.0, 129.8, 129.0, 128.1, 127.9, 127.7, 126.8, 126.7, 120.6, 118.3, 112.5, 107.9, 58.9, 56.4, 56.3, 55.7, 52.6, 52.5, 47.8, 47.0, 39.9, 39.3, 24.9, 22.2; HRMS (ESI): Calc'd for $[M+H]^+$ $C_{23}H_{23}INO_7S$: 584.0240; found 584.0250.

Methyl 2-(3-(((4-*(tert*-butyl)phenyl)sulfonyl)methyl)-4-(iodo(phenyl)methylene)-3-methyl -2,5-dioxopyrrolidin-1-yl)acetate (5af). The title compound was prepared according to the general procedure and purified by silica gel column chromatography (Hex:EA = 7:3) to afforded the desired product **5af** as a white solid (147 mg, yield = 80%, *E/Z* ratio 93:7); mp:156.2-159 ^{o}C; ^{1}H NMR (500 MHz, $CDCl_3$) δ 7.83 (d, J = 8.6 Hz, 1.98H), 7.73 (d, J = 8.7

Hz, 0.20H), 7.58 (d, J = 8.3 Hz, 2.14H), 7.41 – 7.30 (m, 5.26H), 4.59 (d, J = 14.4 Hz, 1H), 4.27 (s, 1.89H), 3.79 (s, 0.25H), 3.72 – 3.68 (m, 3.97H), 3.47 (d, J = 14.5 Hz, 0.08H), 3.10 (d, J = 14.5 Hz, 0.08H), 1.73 (s, 2.93H), 1.36 (s, 9.05H), 1.35 (s, 0.88H), 1.23 (s, 0.29H); ^{13}C NMR (101 MHz, $CDCl_3$) δ 176.8, 166.9, 163.5, 158.0, 144.4, 137.0, 133.9, 129.1, 128.0, 127.7, 126.7, 126.4, 118.9, 57.4, 52.6, 48.0, 39.4, 35.2, 31.0, 22.1; HRMS (ESI): Calc'd for $[M+H]^+$ $C_{26}H_{29}INO_6S$: 610.0760; found 610.0771.

Methyl 2-(3-(((4-acetamidophenyl)sulfonyl)methyl)-4-(iodo(phenyl)methylene)-3-methyl-2,5-dioxopyrrolidin-1-yl)acetate (5ag). The title compound was prepared according to the general procedure and purified by silica gel column chromatography (Hex:EA = 7:3) to afforded the desired product **5ag** as a white solid (129 mg, yield = 70%, *E/Z* ratio 91:9); mp:211.8-214.4 °C; ^{1}H NMR (500 MHz, $CDCl_3$) δ 7.84 (d, J = 8.7 Hz, 2.01H), 7.71 (d, J = 8.4 Hz, 2.32H), 7.56 (s, 1.01H), 7.41 – 7.31 (m, 5.10H), 4.59 (d, J = 14.4 Hz, 1H), 4.28 (s, 1.94H), 3.79 (s, 0.30H), 3.71 (s, 3.01H), 3.68 (d, J = 14.4 Hz, 1.04H), 3.44 (d, J = 14.5 Hz, 0.10H), 3.10 (d, J = 14.4 Hz, 0.10H), 2.22 (s, 2.96H), 2.20 (s, 0.25H), 1.74 (s, 2.99H), 1.24 (s, 0.25H); ^{13}C NMR (101 MHz, $CDCl_3$) δ 176.8, 168.8, 167.0, 163.5, 144.4, 143.3, 134.4, 133.9, 129.2, 129.1, 128.2, 128.0, 126.8, 119.4, 119.1, 57.6, 52.6, 48.1, 39.5, 29.6, 24.7, 22.2; HRMS (ESI): Calc'd for $[M+H]^+$ $C_{24}H_{24}IN_2O_7S$: 611.0349; found 611.0367.

Methyl 2-(3-(((4-fluorophenyl)sulfonyl)methyl)-4-(iodo(phenyl)methylene)-3-methyl-2,5-dioxopyrrolidin-1-yl)acetate (5ah). The title compound was prepared according to the general procedure and purified by silica gel column chromatography (Hex:EA = 7:3) to afforded the desired product **5ah** as a white solid (98 mg, yield = 57%, *E/Z* ratio 90:10); mp:197.5-200.7 °C; ^{1}H NMR (500 MHz, $CDCl_3$) δ 7.97 – 7.91 (m, 2.01H), 7.86 – 7.80 (m, 0.22H), 7.42 – 7.31 (m, 5.49H), 7.29 – 7.26 (m, 1.19H), 7.26 – 7.22 (m, 1.34H), 4.62 (d, J = 14.4 Hz, 1H), 4.29 (s, 1.96H), 3.80 (s, 0.35H), 3.73 – 3.68 (m, 4.01H), 3.46 (d, J = 14.5 Hz, 0.11H), 3.11 (d, J = 14.5 Hz, 0.11H), 1.74 (s, 2.99H), 1.26 (s, 0.32H); ^{13}C NMR (101 MHz, $CDCl_3$) δ 176.8, 166.9, 166.0 (d, J = 257.2 Hz), 163.4, 144.3, 136.2, 133.9, 130.8 (d, J = 9.5 Hz), 129.3, 128.0, 126.8, 119.0, 116.7 (d, J = 22.7 Hz), 60.7, 57.6, 52.6, 48.2, 40.1, 39.4, 25.3, 22.2; HRMS (ESI): Calc'd for $[M+H]^+$ $C_{22}H_{20}FINO_6S$: 572.0040; found 572.0081.

Methyl (*E*)-2-(3-(((4-chlorophenyl)sulfonyl)methyl)-4-(iodo(phenyl)methylene)-3-methyl -2,5-dioxopyrrolidin-1-yl)acetate (5ai). The title compound was prepared according to the general procedure and purified by silica gel column chromatography (Hex:EA = 7:3) to afforded the desired product **5ai** as a white solid (94 mg, yield = 53%, *E*-isomer); mp:188-191 °C; ^{1}H NMR (500 MHz, $CDCl_3$) δ 7.86 (d, J = 8.6 Hz, 1.91H), 7.56 (d, J = 8.6 Hz, 1.95H), 7.42 – 7.36 (m, 2.08H), 7.37 – 7.31 (m, 3.08H), 4.62 (d, J = 14.4 Hz, 1H), 4.29 (s,

1.89H), 3.72 (s, 2.93H), 3.70 (d, *J* = 14.6 Hz, 0.98H), 1.74 (s, 2.90H); ^{13}C NMR (101 MHz, $CDCl_3$) δ 176.7, 166.9, 163.3, 144.3, 140.9, 138.5, 133.8, 129.7, 129.37, 129.32, 128.0, 126.8, 119.1, 57.5, 52.6, 48.1, 39.4, 22.1; HRMS (ESI): Calc'd for $[M+H]^+$ $C_{22}H_{20}ClINO_6S$: 587.9745; found 587.9751.

Methyl 2-(3-(((4-bromophenyl)sulfonyl)methyl)-4-(iodo(phenyl)methylene)-3-methyl-2,5-dioxopyrrolidin-1-yl)acetate (5aj). The title compound was prepared according to the general procedure and purified by silica gel column chromatography (Hex:EA = 7:3) to afforded the desired product **5aj** as a white solid (114 mg, yield = 60%, *E/Z* ratio 73:27); mp:128-131 °C; ^1H NMR (500 MHz, $CDCl_3$) δ 7.82 – 7.76 (m, 2.20H), 7.76 – 7.64 (m, 3.60H), 7.46 – 7.37 (m, 3.07H), 7.37 – 7.30 (m, 3.35H), 4.62 (d, *J* = 14.3 Hz, 1H), 4.29 (s, 1.91H), 3.80 (s, 1.13H), 3.72 (s, 2.93H), 3.69 (d, *J* = 14.4 Hz, 0.86H), 3.45 (d, *J* = 14.5 Hz, 0.38H), 3.10 (d, *J* = 14.4 Hz, 0.37H), 1.74 (s, 3.11H), 1.26 (s, 1.27H); ^{13}C NMR (101 MHz, $CDCl_3$) δ 176.7, 175.0, 166.99, 166.90, 165.4, 163.3, 144.3, 143.4, 139.1, 133.9, 132.76, 132.70, 129.5, 129.4, 129.3, 128.2, 128.0, 126.8, 126.7, 119.1, 108.6, 92.2, 60.7, 57.6, 52.7, 52.6, 48.1, 47.3, 40.1, 39.5, 25.3, 22.2; HRMS (ESI): Calc'd for $[M+H]^+$ $C_{22}H_{20}BrINO_6S$: 631.9239; found 631.9244.

Methyl 2-(4-(iodo(phenyl)methylene)-3-methyl-2,5-dioxo-3-(((4-(trifluoromethyl)phenyl)sulfonyl)methyl)pyrrolidin-1-yl)acetate (5ak). The title compound was prepared according to the general procedure and purified by silica gel column chromatography (Hex:EA = 7:3) to afforded the desired product **5ak** as a white solid (138 mg, yield = 74%, *E/Z* ratio 92:8); mp:176.5-178.6 °C; ^1H NMR (500 MHz, $CDCl_3$) δ 8.07 (d, *J* = 8.3 Hz, 1.99H), 7.95 (d, *J* = 8.3 Hz, 0.15H), 7.86 (d, *J* = 8.4 Hz, 2.05H), 7.83 (d, *J* = 8.5 Hz, 0.23H), 7.42 – 7.37 (m, 2.03H), 7.37 – 7.31 (m, 3.19H), 4.66 (d, *J* = 14.4 Hz, 1H), 4.30 (s, 1.95H), 3.81 (s, 0.22H), 3.76 – 3.70 (m, 4.02H), 3.47 (d, *J* = 14.4 Hz, 0.09H), 3.13 (d, *J* = 14.5 Hz, 0.09H), 1.75 (s, 2.99H), 1.29 (s, 0.21H); ^{13}C NMR (101 MHz, CDCl3 δ 176.6, 166.8, 163.3, 144.2, 143.5, 135.7 (d, *J* = 33.6 Hz), 133.8, 129.3, 128.3 (d, *J* = 48.1 Hz), 126.7, 126.6, 123.0 (d, *J* = 273.1 Hz), 119.2, 57.4, 52.6, 48.2, 40.2, 39.4, 25.4, 22.2; HRMS (ESI): Calc'd for $[M+H]^+$ $C_{23}H_{20}F_3INO_6S$: 622.0008; found 622.0025.

Methyl 2-(3-(((2,5-dichlorophenyl)sulfonyl)methyl)-4-(iodo(phenyl)methylene)-3-methyl-2,5-dioxopyrrolidin-1-yl)acetate (5al). The title compound was prepared according to the general procedure and purified by silica gel column chromatography (Hex:EA = 7:3) to afforded the desired product **5al** as a white solid (90 mg, yield = 48%, *E/Z* ratio 74:26); mp:159-162.8 °C; ^1H NMR (500 MHz, $CDCl_3$) δ 8.01 (d, *J* = 2.4 Hz, 0.92H), 7.92 (d, *J* = 2.5 Hz, 0.28H), 7.60 – 7.50 (m, 2.55H), 7.49 – 7.41 (m, 1.66H), 7.41 – 7.36 (m, 2.35H), 7.33 (d,

J = 7.2 Hz, 1.31H), 7.28 (d, J = 7.2 Hz, 2.89H), 5.00 (d, J = 14.8 Hz, 1H), 4.49 (d, J = 17.0 Hz, 0.34H), 4.28 (s, 2.04H), 3.93 (d, J = 14.7 Hz, 0.92H), 3.79 (s, 1.11H), 3.77 – 3.68 (m, 3.39H), 3.38 (d, J = 14.7 Hz, 0.32H), 1.77 (s, 2.98H), 1.29 (s, 1.18H); ^{13}C NMR (101 MHz, $CDCl_3$) δ 176.3, 174.5, 166.8, 165.3, 163.3, 144.1, 143.4, 139.1, 135.0, 134.9, 134.0, 133.9, 133.8, 133.1, 133.0, 131.3, 130.8, 130.6, 129.4, 129.3, 128.3, 128.0, 126.8, 118.8, 108.5, 58.7, 55.9, 52.7, 52.6, 48.1, 47.2, 40.0, 39.4, 25.4, 22.3; HRMS (ESI): Calc'd for $[M+H]^+$ $C_{22}H_{19}Cl_2INO_6S$: 621.9355; found 621.9365.

Methyl (*E*)-2-(4-(iodo(phenyl)methylene)-3-methyl-2,5-dioxo-3-(((4-(trifluoromethoxy) phenyl)sulfonyl)methyl)pyrrolidin-1-yl)acetate (5am). The title compound was prepared according to the general procedure and purified by silica gel column chromatography (Hex:EA = 7:3) to afforded the desired product **5am** as a white solid (142 mg, yield = 74%, *E*- isomer); mp:162-164.8 ^{o}C; ^{1}H NMR (500 MHz, $CDCl_3$) δ 7.98 (d, J = 8.8 Hz, 1.95H), 7.40 (dd, J = 11.4, 7.8 Hz, 4.04H), 7.34 (d, J = 7.0 Hz, 3.26H), 4.64 (d, J = 14.4 Hz, 1H), 4.29 (s, 1.92H), 3.76 – 3.68 (m, 4.10H), 1.75 (s, 3H); ^{13}C NMR (101 MHz, $CDCl_3$) δ 176.7, 166.9, 163.4, 153.2, 144.3, 138.3, 133.8, 130.2, 129.3, 128.0, 126.7, 121.1, 120.1 (d, J = 259.9 Hz), 119.2, 57.5, 52.6, 48.2, 39.4, 22.1; HRMS (ESI): Calc'd for $[M+H]^+$ $C_{23}H_{20}F_3INO_7S$: 637.9957; found 637.9942.

Methyl (*E*)-2-(4-(iodo(phenyl)methylene)-3-methyl-3-((naphthalen-2-ylsulfonyl) meth - yl)-2,5-dioxopyrrolidin-1-yl)acetate (5an). The title compound was prepared according to the general procedure and purified by silica gel column chromatography (Hex:EA = 7:3) to afforded the desired product **5an** as a white solid (112 mg, yield = 62%, *E*- isomer); mp:206-209 ^{o}C; ^{1}H NMR (500 MHz, $CDCl_3$) δ 8.48 (s, 0.93H), 8.03 (d, J = 8.6 Hz, 1.02H), 7.99 (d, J = 8.2 Hz, 1.08H), 7.96 (d, J = 8.2 Hz, 1.06H), 7.88 (dd, J = 8.7, 1.7 Hz, 1.02H), 7.71 (t, J = 7.6 Hz, 1.08H), 7.65 (t, J = 7.6 Hz, 1.05H), 7.38 – 7.34 (m, 2.02H), 7.32 (d, J = 7.0 Hz, 1.04H), 7.30 (d, J = 1.4 Hz, 1.28H), 7.28 (s, 0.86H), 4.68 (d, J = 14.4 Hz, 1H), 4.25 (s, 1.94H), 3.79 (d, J = 14.4 Hz, 1.22H), 3.71 (s, 2.92H), 1.75 (s, 3.09H); ^{13}C NMR (101 MHz, $CDCl_3$) δ 176.7, 166.9, 163.4, 144.4, 136.7, 135.4, 133.9, 132.0, 129.85, 129.81, 129.5, 129.2, 127.9, 127.8, 126.7, 122.3, 119.0, 57.4, 52.6, 48.1, 39.4, 22.2; HRMS (ESI): Calc'd for $[M+H]^+$ $C_{26}H_{23}INO_6S$: 604.0291; found 604.0303.

Methyl 2-(4-(iodo(phenyl)methylene)-3-methyl-3-((methylsulfonyl)methyl)-2,5-dioxo pyrrolidin-1-yl)acetate (5ao). The title compound was prepared according to the general procedure and purified by silica gel column chromatography (Hex:EA = 7:3) to afforded the desired product **5ao** as a white solid (108 mg, yield = 73%, *E/Z* ration 84:16); mp:195-197 ^{o}C; ^{1}H NMR (500 MHz, $CDCl_3$) δ 7.48 – 7.40 (m, 0.63H), 7.39 (d, J = 1.8 Hz, 0.35H), 7.37

(d, *J* = 6.6 Hz, 1.78H), 7.34 – 7.30 (m, 3.10H), 4.60 (d, *J* = 14.5 Hz, 1H), 4.44 (q, *J* = 18.9 Hz, 0.45H), 4.27 (q, *J* = 13.5 Hz, 2.16H), 3.79 (s, 0.48H), 3.70 (s, 2.91H), 3.68 (d, *J* = 14.6 Hz, 1.14H), 3.40 (d, *J* = 14.8 Hz, 0.18H), 3.02 – 2.98 (m, 3.08H), 2.85 (s, 0.57H), 1.77 (s, 2.99H), 1.34 (s, 0.55H); ^{13}C NMR (101 MHz, $CDCl_3$) δ 177.1, 167.2, 167.1, 163.5 144.5, 143.6, 134.4, 133.4, 129.6, 128.8, 128.3, 127.1, 118.4, 58.9, 56.3, 53.0, 52.8, 48.3, 47.9, 44.2, 44.0, 40.3, 39.7, 25.8, 22.5; HRMS (ESI): Calc'd for $[M+H]^+$ $C_{17}H_{19}INO_6S$: 491.9978; found 491.9995.

Ethyl (*E*)-2-(4-(iodo(phenyl)methylene)-3-methyl-2,5-dioxo-3-(tosylmethyl)pyrrolidin-1-yl)acetate (5ba). The title compound was prepared according to the general procedure and purified by silica gel column chromatography (Hex:EA = 7:3) to afforded the desired product **5ab** as a white solid (131 mg, yield = 75%, *E*-isomer); mp:175.5-178 °C; ^{1}H NMR (500 MHz, $CDCl_3$) δ 7.79 (d, *J* = 8.3 Hz, 1.93H), 7.43 – 7.29 (m, 7.05H), 4.59 (d, *J* = 14.4 Hz, 0.97H), 4.25 (s, 1.85H), 4.20 – 4.11 (m, 1.98H), 3.69 (d, *J* = 14.4 Hz, 1H), 2.47 (s, 2.90H), 1.73 (s, 2.93H), 1.24 (t, *J* = 7.2 Hz, 3.10H); ^{13}C NMR (101 MHz, $CDCl_3$) δ 176.8, 166.4, 163.4, 145.1, 144.4, 137.2, 134.0, 129.9, 129.1, 127.9, 127.8, 126.8, 118.7, 61.8, 57.4, 48.0, 39.5, 22.2, 21.6, 13.9; HRMS (ESI): Calc'd for $[M+H]^+$ $C_{24}H_{25}INO_6S$: 582.0447; found 582.0422.

(*S*)-methyl 2-(4-(iodo(phenyl)methylene)-3-methyl-2,5-dioxo-3-(tosylmethyl)pyrrolidin-1-yl)propanoate (5ca). The title compound was prepared according to the general procedure and purified by silica gel column chromatography (Hex:EA = 7:3) to afforded the desired product **5ca** as a white solid (128 mg, yield = 73%, *E/Z* ratio 83:17, dr ratio 1:1.05); mp:155-158 °C; ^{1}H NMR (500 MHz, $CDCl_3$) δ 7.81 (d, *J* = 3.3 Hz, 1.76H), 7.79 (d, *J* = 3.2 Hz, 1.90H), 7.70 (d, *J* = 2.1 Hz, 0.33H), 7.69 (d, *J* = 2.1 Hz, 0.36H), 7.43 – 7.28 (m, 16.32H), 5.06 (q, *J* = 7.0 Hz, 0.28H), 5.01 (q, *J* = 7.0 Hz, 0.20H), 4.92 (q, *J* = 7.3 Hz, 0.98H), 4.79 (q, *J* = 7.3 Hz, 0.99H), 4.58 (d, *J* = 14.3 Hz, 1H), 4.56 (d, *J* = 14.2 Hz, 0.91H), 3.81 (s, 0.41H), 3.77 (s, 0.64H), 3.72 (s, 2.84H), 3.70 – 3.66 (m, 3.71), 3.64 (d, *J* = 14.2 Hz, 0.98H), 3.44 (d, *J* = 14.2 Hz, 0.20H), 3.41 (d, *J* = 14.4 Hz, 0.21H), 3.08 (d, *J* = 14.3 Hz, 0.21H), 3.05 (d, *J* = 14.4 Hz, 0.18H), 2.47 (s, 2.90H), 2.46 (s, 2.75H), 2.44 (s, 0.54H), 2.44 (s, 0.55H), 1.77 (d, *J* = 7.3 Hz, 0.57H), 1.73 (d, *J* = 7.3 Hz, 0.79H), 1.71 (s, 2.77H), 1.68 (s, 2.71H), 1.65 (d, *J* = 7.3 Hz, 2.93H), 1.61 (d, *J* = 7.3 Hz, 2.84H), 1.22 (s, 0.36H), 1.19 (s, 0.54H); ^{13}C NMR (101 MHz, $CDCl_3$) δ 176.7, 176.6, 169.6, 169.4, 163.3, 163.2, 145.0, 144.6, 144.4, 137.4, 134.2, 134.0, 130.0, 129.2, 129.1, 128.0, 127.9, 127.7, 127.0, 126.7, 118.5, 118.4, 60.8, 57.69, 57.65, 52.8, 52.6, 48.9, 48.2, 48.0, 47.6, 25.2, 25.0, 22.1, 21.8, 21.6, 13.7, 13.3; HRMS (ESI): Calc'd for $[M+H]^+$ $C_{24}H_{25}INO_6S$: 582.0447; found 582.0441.

(*S,E*)-methyl 2-(4-(iodo(phenyl)methylene)-3-methyl-2,5-dioxo-3-(tosylmethyl) pyrrolidin-1-yl)-3-methyl butanoate (5da). The title compound was prepared according to the general procedure and purified by silica gel column chromatography (Hex:EA = 7:3) to afforded the desired product **5da** as a white solid (115 mg, yield = 63%, *E*-isomer, dr ratio 1:1.08); mp:175-177.3 °C; ^{1}H NMR (500 MHz, $CDCl_3$) δ 7.82 (d, *J* = 3.9 Hz, 2.10H), 7.80 (d, *J* = 3.8 Hz, 2.04H), 7.39 – 7.36 (m, 11.34H), 7.35 – 7.31 (m, 2.73H), 4.59 (d, *J* = 14.3 Hz, 0.92H), 4.56 (d, *J* = 14.4 Hz, 0.98H), 4.47 (d, *J* = 7.8 Hz, 0.96H), 4.44 (d, *J* = 8.8 Hz, 1.01H), 3.71 (d, *J* = 14.2 Hz, 1H),, 3.69 (s, 5.65H), 3.68 (d, *J* = 14.3 Hz, 1.10H), 2.76 – 2.69 (m, 0.98H), 2.69 – 2.62 (m, 1.09H), 2.46 (s, 6.15H), 1.67 (s, 2.97H), 1.65 (s, 3.04H), 1.12 (d, *J* = 3.2 Hz, 2.86H), 1.11 (d, *J* = 3.3 Hz, 2.82H), 1.04 (d, *J* = 6.9 Hz, 2.97H), 0.92 (d, *J* = 6.8 Hz, 2.99H); ^{13}C NMR (101 MHz, $CDCl_3$) δ 177.1, 177.0, 168.6, 168.4, 164.1, 163.8, 144.9, 144.5, 144.4, 137.6, 137.5, 134.17, 134.10, 130.0, 129.2, 128.0, 127.7, 127.2, 127.0, 118.5, 118.3, 58.6, 58.4, 57.3, 57.2, 52.3, 52.1, 48.2, 48.0, 28.8, 27.6, 22.7, 22.4, 21.6, 20.9, 20.4, 19.3, 19.2; HRMS (ESI): Calc'd for $[M+H]^+$ $C_{26}H_{29}INO_6S$: 610.0760; found 610.0770.

(*S,E*)-methyl 2-(4-(iodo(phenyl)methylene)-3-methyl-2,5-dioxo-3-(tosyl methyl) pyrrolidin-1-yl)-4-methylpentanoate (5ea). The title compound was prepared according to the general procedure and purified by silica gel column chromatography (Hex:EA = 7:3) to afforded the desired product **5ea** as a white solid (138 mg, yield = 74%, *E*-isomer, dr ratio 1:1.08); mp:163-165.7 °C; ^{1}H NMR (500 MHz, $CDCl_3$) δ 7.80 (d, *J* = 7.9 Hz, 4.18H), 7.40 – 7.31 (m, 14.71H), 4.88 (dd, *J* = 10.3, 5.1 Hz, 1H), 4.77 (dd, *J* = 10.4, 4.4 Hz, 1.07H), 4.59 (d, *J* = 14.4 Hz, 1.05H), 4.56 (d, *J* = 14.3 Hz, 1.02H), 3.70 (s, 3.60H), 3.68 (s, 3.52H), 3.66 (d, *J* = 14.4 Hz, 1.38H), 2.46 (s, 6.03H), 2.27 – 2.20 (m, 1.10H), 2.20 – 2.13 (m, 1.11H), 1.96 – 1.90 (m, 1.15H), 1.90 – 1.85 (m, 1.06H), 1.80 – 1.71 (m, 2.28H), 1.68 (s, 2.97H), 1.67 (s, 3.17H), 0.96 (d, *J* = 6.5 Hz, 3.13H), 0.93 (d, *J* = 3.6 Hz, 3.12H), 0.92 (d, *J* = 3.7 Hz, 3.21H), 0.90 (d, *J* = 6.5 Hz, 2.83H); ^{13}C NMR (101 MHz, $CDCl_3$) δ 177.1, 177.0, 169.7, 169.5, 163.7, 144.9, 144.5, 144.4, 137.7, 137.6, 134.28, 134.20, 129.9, 129.25, 129.21, 128.0, 127.7, 127.2, 126.9, 118.3, 118.0, 57.4, 52.6, 52.5, 51.4, 48.3, 47.8, 37.0, 36.4, 24.5, 23.0, 22.5, 22.2, 21.6, 21.3, 21.2; HRMS (ESI): Calc'd for $[M+H]^+$ $C_{27}H_{31}INO_6S$: 624.0917; found 624.0903.

(2*S*,3*R*)-methyl 2-((*S,E*)-4-(iodo(phenyl)methylene)-3-methyl-2,5-dioxo-3-(tosylmethyl) pyrrolidin-1-yl)-3-methylpentanoate (5fa). The title compound was prepared according to the general procedure and purified by silica gel column chromatography (Hex:EA = 7:3) to afforded the desired product **5fa** as a white solid (110 mg, yield = 59%, *E*-isomer, dr ratio 1:1.05); mp:140.6-143.3 °C; ^{1}H NMR (500 MHz, $CDCl_3$) δ 7.88 – 7.74 (m, 3.98H), 7.37 (s, 11.89H), 7.35 – 7.32 (m, 1.91H), 4.68 – 4.45 (m, 4H), 3.85 – 3.61 (m, 8.16H), 2.56 – 2.38

(m, 7.81H), 1.74 – 1.62 (m, 6.96H), 1.54 – 1.44 (m, 1.13H), 1.28 – 1.19 (m, 1.15H), 1.18 – 1.00 (m, 6.77H), 0.97 – 0.79 (m, 5.81H); ^{13}C NMR (101 MHz, $CDCl_3$) δ 177.1, 177.0, 168.7, 168.4, 164.1, 163.8, 144.9, 144.5, 144.4, 137.77, 137.70, 134.2, 134.1, 130.0, 129.2, 128.0, 127.7, 127.2, 127.0, 118.4, 118.3, 58.0, 57.6, 57.4, 57.3, 52.2, 52.1, 48.2, 48.0, 35.0, 33.5, 25.7, 25.3, 22.6, 22.3, 21.6, 16.5, 16.2, 11.2, 10.8; HRMS (ESI): Calc'd for $[M+H]^+$ $C_{27}H_{31}INO_6S$: 624.0917; found 624.0903.

(*S,E*)-methyl 2-(4-(iodo(phenyl)methylene)-3-methyl-2,5-dioxo-3-(tosylmethyl) pyrrolidin-1-yl)-4-(methylthio)butanoate (5ga). The title compound was prepared according to the general procedure and purified by silica gel column chromatography (Hex:EA = 7:3) to afforded the desired product **5ga** as a white solid (54 mg, yield = 28%, *E*-isomer, dr ratio 1:2.3); mp:168-171 ^{o}C; ^{1}H NMR (500 MHz, $CDCl_3$) δ 7.81 – 7.75 (m, 2.85H), 7.42 – 7.29 (m, 10.80H), 5.13 (dd, *J* = 8.7, 5.8 Hz, 0.43H), 4.90 (dd, *J* = 9.4, 4.6 Hz, 1H), 4.59 (d, *J* = 14.3 Hz, 0.73H), 4.56 (d, *J* = 14.3 Hz, 0.76H), 3.72 (s, 2.91H), 3.71 – 3.67 (m, 1.86H), 3.65 (d, *J* = 14.3 Hz, 1.08H), 2.78 – 2.68 (m, 1.53H), 2.68 – 2.55 (m, 1.46H), 2.55 – 2.49 (m, 1.79H), 2.46 (s, 4.56H), 2.46 – 2.39 (m, 1.76H), 2.09 (s, 1.30H), 2.07 (s, 2.87H), 1.70 (s, 1.25H), 1.68 (s, 2.88H); ^{13}C NMR (101 MHz, $CDCl_3$) δ 177.0, 168.9, 163.7, 163.6, 145.0, 144.5, 144.4, 137.5, 137.4, 134.09, 134.01, 130.0, 129.2, 128.0, 127.7, 127.0, 126.8, 118.8, 118.4, 57.6, 57.4, 52.7, 51.9, 51.4, 48.3, 47.8, 30.3, 28.0, 27.0, 22.3, 22.0, 21.6, 15.1, 15.0; HRMS (ESI): Calc'd for $[M+H]^+$ $C_{26}H_{29}INO_6S_2$: 642.0481; found 642.0458.

Methyl 2-(4-(iodo(phenyl)methylene)-3-(iodomethyl)-3-methyl-2,5-dioxopyrrolidin-1-yl)acetate (6a). The title compound was prepared according to the general procedure and purified by silica gel column chromatography (Hex:EA = 9:1) to afforded the desired product **6a** as a white solid (125 mg, yield = 77%, *Z/E* ratio 66:34,); mp:118-121.2 ^{o}C; ^{1}H NMR (500 MHz, $CDCl_3$) δ 7.57 (s, 0.77H), 7.45 (s, 2.25H), 7.40 (t, *J* = 7.8 Hz, 2.01H), 7.35 (t, *J* = 7.5 Hz, 1.73H), 7.22 (s, 0.74H), 4.45 (q, *J* = 14.5 Hz, 2.12H), 4.32 – 4.26 (m, 1.47H), 3.82 (s, 3.03H), 3.73 (s, 1.53H), 3.59 (d, *J* = 10.2 Hz, 0.52H), 3.36 (d, *J* = 10.1 Hz, 1H), 2.61 (d, *J* = 10.1 Hz, 1.04H), 1.88 (s, 1.48H), 1.45 (s, 3.13H); ^{13}C NMR (126 MHz, $CDCl_3$) δ 176.9, 175.3, 166.6, 166.4, 165.3, 163.2, 144.5, 143.1, 136.6, 135.2, 129.3, 129.1, 128.0, 126.5, 117.3, 107.2, 52.6, 52.5, 51.7, 51.4, 39.9, 39.2, 23.5, 20.4, 8.6, 6.3; HRMS (ESI): Calc'd for $[M+H]^+$ $C_{16}H_{16}I_2NO_4$: 539.9169; found 539.9168.

Ethyl (*Z*)-2-(4-(iodo(phenyl)methylene)-3-(iodomethyl)-3-methyl-2,5-dioxopyrrolidin-1-yl)acetate (6b). The title compound was prepared according to the general procedure and purified by silica gel column chromatography (Hex:EA = 9:1) to afforded the desired product **6b** as a white solid (116 mg, yield = 70%, *Z*-isomer); mp:157-160.2 ^{o}C; ^{1}H NMR (500 MHz,

$CDCl_3$) δ 7.57 (s, 0.94H), 7.45 (s, 2.05H), 7.42 – 7.35 (m, 1.05H), 7.22 (s, 1.03H), 4.43 (q, *J* = 17.6 Hz, 2.07H), 4.34 – 4.21 (m, 2.16H), 3.36 (d, *J* = 10.1 Hz, 1H), 2.60 (d, *J* = 10.1 Hz, 1.01H), 1.45 (s, 3.05H), 1.33 (t, *J* = 7.2 Hz, 3.03H); ^{13}C NMR (126 MHz, $CDCl_3$) δ 175.3, 166.1, 165.3, 143.1, 135.2, 129.3, 128.2, 125.8, 125.3, 107.1, 61.9, 51.7, 40.0, 23.6, 14.0, 8.5; HRMS (ESI): Calc'd for $[M+H]^+$ $C_{17}H_{18}I_2NO_4$: 553.9325; found 553.9319.

(*S*)-methyl 2-(4-(iodo(phenyl)methylene)-3-(iodo methyl)-3-methyl-2,5-dioxopyrrolidin-1-yl)propanoate (6c). The title compound was prepared according to the general procedure and purified by silica gel column chromatography (Hex:EA = 9:1) to afforded the desired product **6c** as a white solid (108 mg, yield = 65%, *Z/E* ratio73:27, dr ratio 1:1.05); mp:117-120.7 °C; ^1H NMR (500 MHz, $CDCl_3$) δ 7.55 (s, 1.65H), 7.45 (s, 4.79H), 7.42 – 7.32 (m, 5.38), 7.21 (s, 1.65H), 5.07 – 4.95 (m, 1.93H), 4.87 – 4.75 (m, 0.80H), 4.28 (d, *J* = 10.1 Hz, 0.67H), 3.80 (s, 3H), 3.78 (s, 2.86H), 3.72 (s, 1.11H), 3.70 (s, 0.84H), 3.60 – 3.53 (m, 0.72H), 3.36 (d, *J* = 9.9 Hz, 1.05H), 3.34 (d, *J* = 9.6 Hz, 0.80H), 2.64 (d, *J* = 10.1 Hz, 1.01H), 2.62 (d, *J* = 9.9 Hz, 0.89H), 1.85 (d, *J* = 2.9 Hz, 1.94H), 1.70 (s, 2.99H), 1.68 (s, 3.02H), 1.60 (d, *J* = 7.2 Hz, 1.97H), 1.41 (s, 2.86H), 1.39 (s, 2.79H); ^{13}C NMR (126 MHz, $CDCl_3$) δ 175.3, 175.1, 169.29, 169.25, 165.29, 165.25, 143.4, 135.4, 135.2, 129.4, 129.27, 129.23, 128.1, 126.7, 126.5, 117.2, 107.3, 107.0, 52.8, 52.7, 52.6, 51.3, 51.1, 51.0, 50.8, 48.7, 48.6, 48.2, 48.0, 23.4, 23.2, 20.4, 20.1, 14.4, 14.3, 14.2, 9.3, 9.2, 6.9, 6.7; HRMS (ESI): Calc'd for $[M+H]^+$ $C_{17}H_{18}I_2NO_4$: 553.9325; found 553.9327.

(*S,Z*)-methyl 2-(4-(iodo(phenyl)methylene)-3-(iodomethyl)-3-methyl-2,5-dioxopyrrolidin-1-yl)-3-methylbutanoate (6d). The title compound was prepared according to the general procedure and purified by silica gel column chromatography (Hex:EA = 9:1) to afforded the desired product **6d** as a white solid (103 mg, yield = 59%, *Z*-isomer, dr ratio 1:1.13); mp:130.5-133.5 °C; ^1H NMR (500 MHz, $CDCl_3$) δ 7.62 (s, 1.64H), 7.45 (s, 3.91H), 7.38 (t, *J* = 7.3 Hz, 2.41H), 7.21 (s, 1.77H), 4.63 (d, *J* = 8.0 Hz, 0.94H), 4.57 (d, *J* = 8.4 Hz, 0.91H), 3.77 (s, 2.72H), 3.74 (s, 3H), 3.38 (d, *J* = 10.1 Hz, 0.90H), 3.36 (d, *J* = 10.1 Hz, 1H), 2.84 – 2.71 (m, 1.95H), 2.54 (d, *J* = 10.1 Hz, 1.04H), 2.51 (d, *J* = 10.1 Hz, 0.97H), 1.44 (s, 2.73H), 1.41 (s, 3.03H), 1.21 (d, *J* = 2.4 Hz, 2.60H), 1.20 (d, *J* = 2.4 Hz, 2.92H), 1.03 (d, *J* = 6.9 Hz, 2.82H), 0.97 (d, *J* = 6.9 Hz, 2.87H); ^{13}C NMR (126 MHz, $CDCl_3$) δ 175.6, 175.5, 168.29, 168.27, 165.7, 165.6, 143.2, 135.1, 134.9, 129.3, 128.3, 128.0, 125.7, 107.1, 107.0, 58.6, 58.4, 52.34, 52.31, 51.58, 51.54, 28.4, 27.7, 24.3, 24.0, 21.0, 20.8, 19.6, 8.6, 8.1; HRMS (ESI): Calc'd for $[M+H]^+$ $C_{19}H_{22}I_2NO_4$: 581.9638; found 581.9663.

(*S,Z*)-methyl 2-(4-(iodo(phenyl)methylene)-3-(iodomethyl)-3-methyl-2,5-dioxopyrrolidin -1-yl)-4-methylpentanoate (6e).

The title compound was prepared according to the general procedure and purified by silica gel column chromatography (Hex:EA = 9:1) to afforded the desired product **6e** as a white solid (110 mg, yield = 62%, *Z*-isomer, dr ratio 1:1.7); mp:138.5-142 ^{o}C; ^{1}H NMR (500 MHz, $CDCl_3$) δ 7.50 (s, 1.50H), 7.36 (s, 3.26H), 7.32 – 7.26 (m, 2.14H), 7.13 (s, 1.44H), 4.90 (dd, *J* = 11.3, 4.2 Hz, 1.07H), 4.86 (dd, *J* = 11.2, 4.6 Hz, 0.54H), 3.69 (s, 1.77H), 3.67 (s, 3H), 3.27 (d, *J* = 10.1 Hz, 0.76H), 3.26 (d, *J* = 10.1 Hz, 0.83H), 2.49 (d, *J* = 10.1 Hz, 1.05H), 2.45 (d, *J* = 10.1 Hz, 0.59H), 2.25 – 2.16 (m, 1.62H), 1.92 – 1.85 (m, 1.60H), 1.63 – 1.54(m, 1.62H), 1.34 (s, 1.64H), 1.32 (s, 3.07H), 0.94 – 0.87 (m, 9.66H); ^{13}C NMR (126 MHz, $CDCl_3$) δ 175.6, 175.4, 169.26, 169.24, 165.5, 143.3, 143.2, 135.2, 135.0, 129.3, 128.3, 128.2, 128.07, 128.03, 125.9, 125.8, 125.6, 107.1, 106.7, 52.71, 52.70, 51.8, 51.7, 51.6, 51.3, 36.7, 36.3, 24.97, 24.91, 24.0, 23.6, 23.18, 23.10, 21.0, 20.9, 8.9, 8.4; HRMS (ESI): Calc'd for $[M+H]^+$ $C_{20}H_{24}I_2NO_4$: 595.9795; found 595.9814.

(9) References:

1. R. Singh, B. K. Allam, N. Singh, K. Kumari, S. K. Singh, and K. N. Singh, *Org. Lett.,* 2015, **17**, 2656-2659.
2. Y. Gu, L. Dai, K. Mao, J. Zhang, C. Wang, L. Zhao and L Rong, *Org. Lett.*, 2020, **22**, 2956-2960.

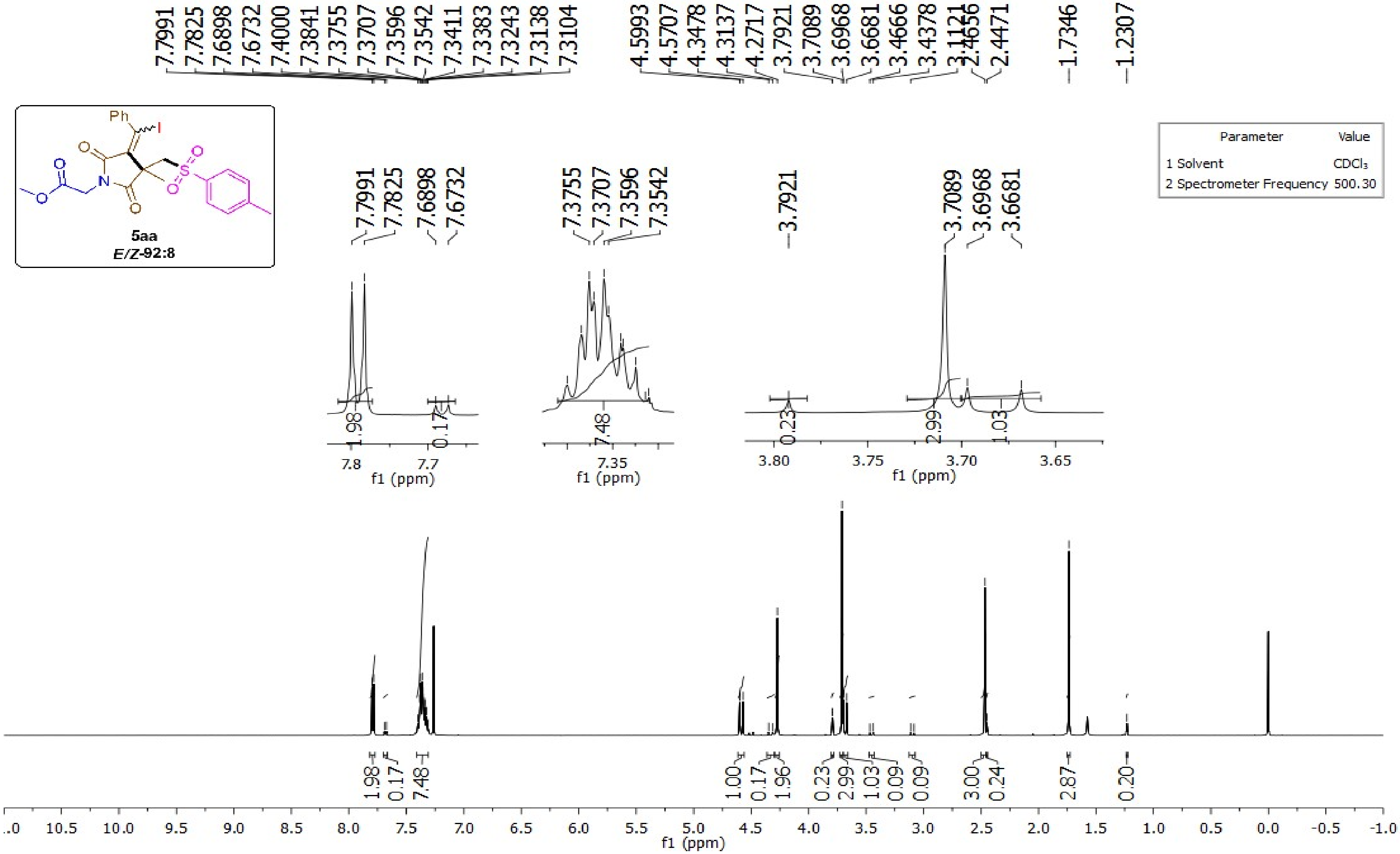

Figure S1. ^{1}H NMR spectra of Methyl 2-(4-(iodo(phenyl)methylene)-3-methyl-2,5-dioxo-3-(tosylmethyl)pyrrolidin-1-yl)acetate (**5aa)**

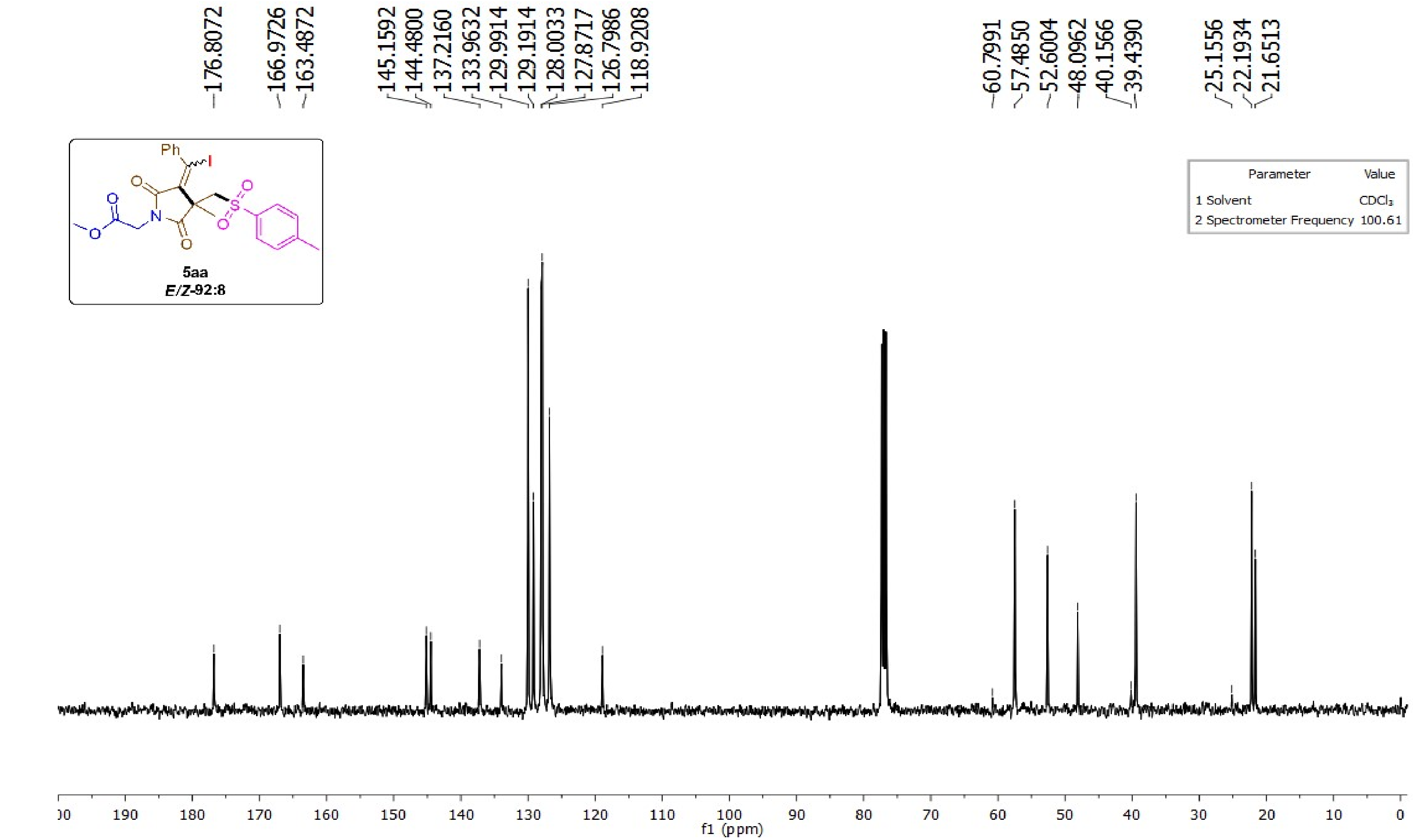

Figure S2. ^{13}C NMR spectra of Methyl 2-(4-(iodo(phenyl)methylene)-3-methyl-2,5-dioxo-3-(tosylmethyl)pyrrolidin-1-yl)acetate (**5aa**)

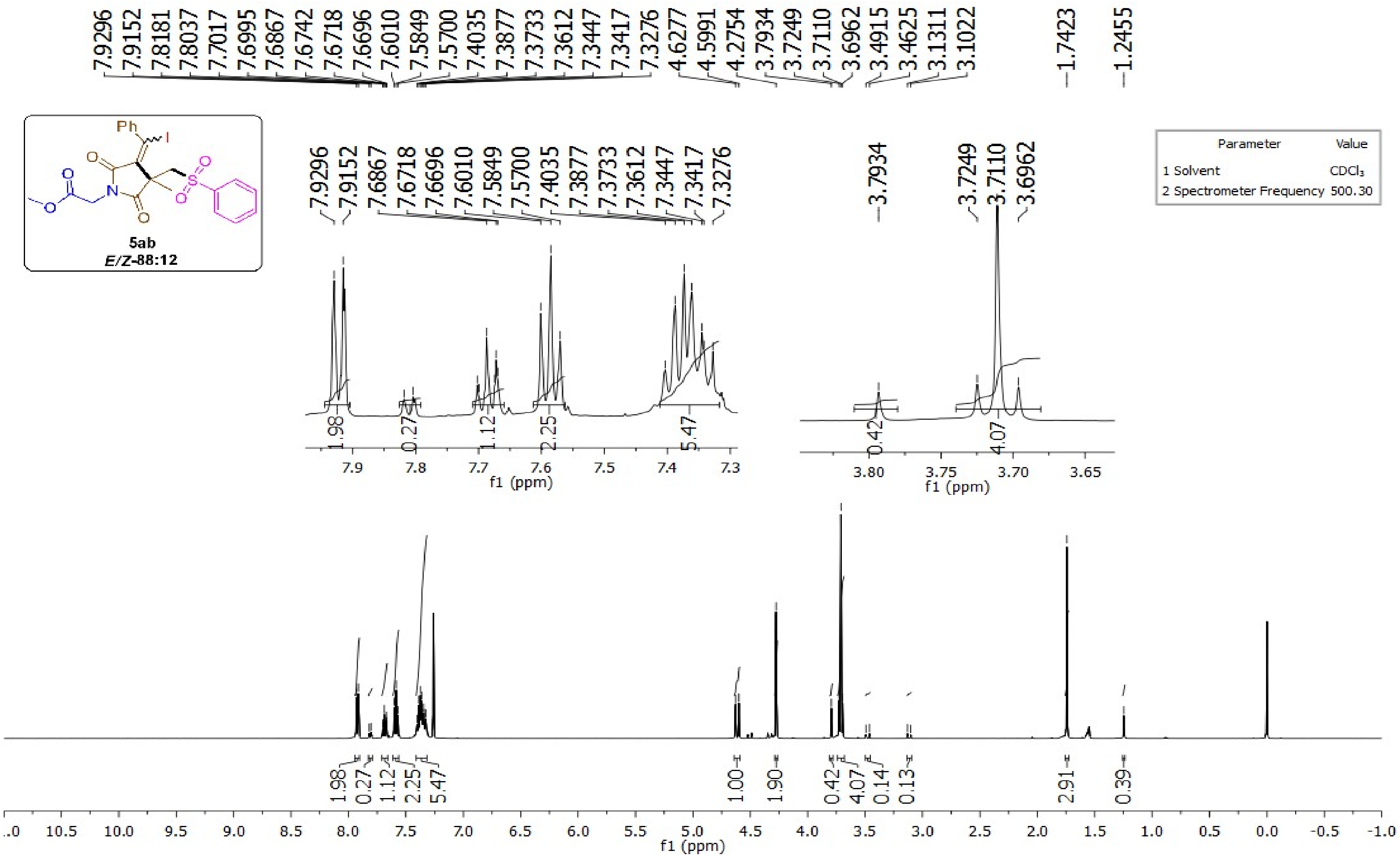

Figure S3. ^{1}H NMR spectra of Methyl 2-(4-(iodo(phenyl)methylene)-3-methyl-2,5-dioxo-3-((phenylsulphonyl)methyl)pyrrolidin-1-yl)acetate (**5ab**)

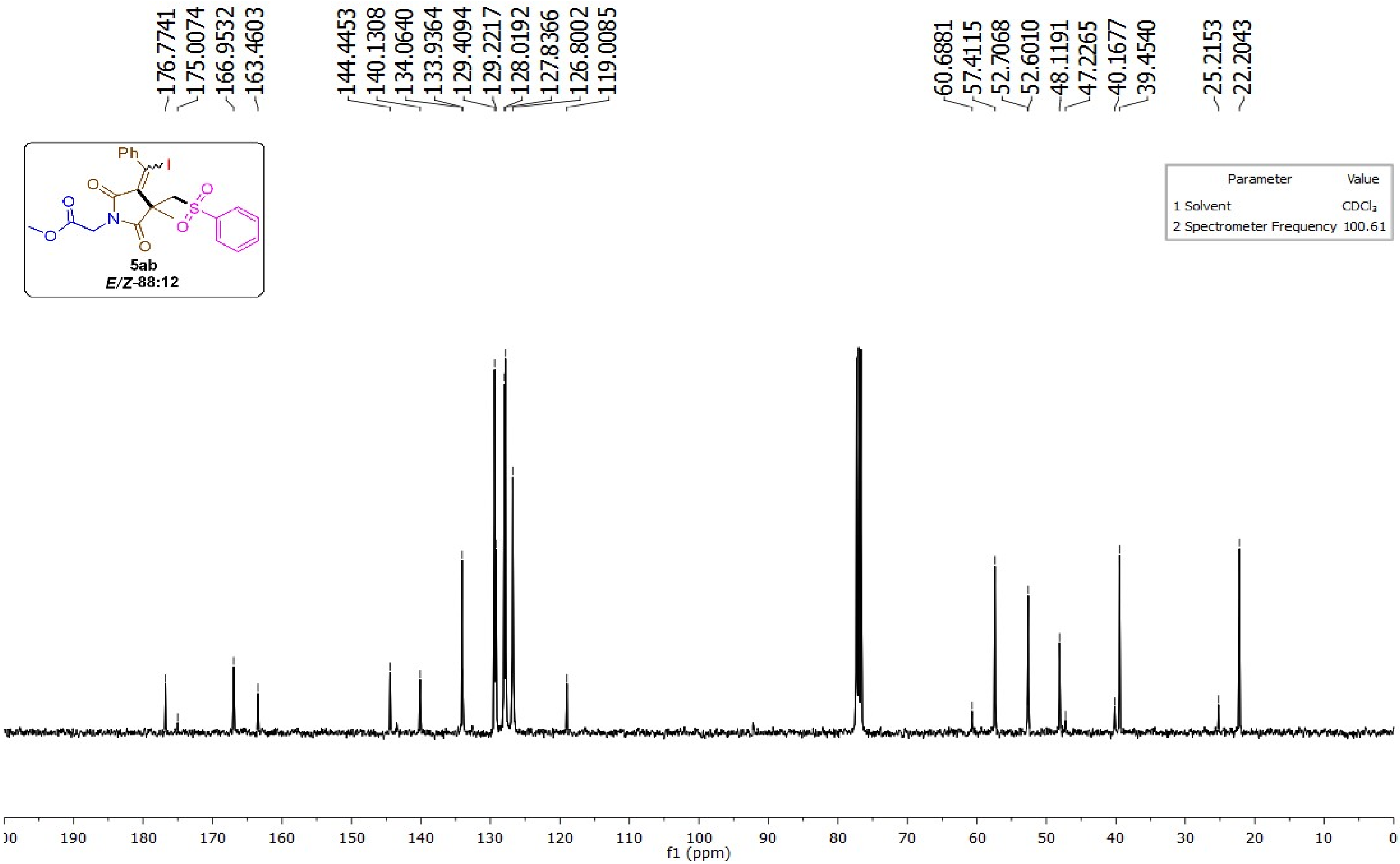

Figure S4. ^{13}C NMR spectra of Methyl 2-(4-(iodo(phenyl)methylene)-3-methyl-2,5-dioxo-3-((phenylsulphonyl)methyl)pyrrolidin-1-yl)acetate (**5ab**)

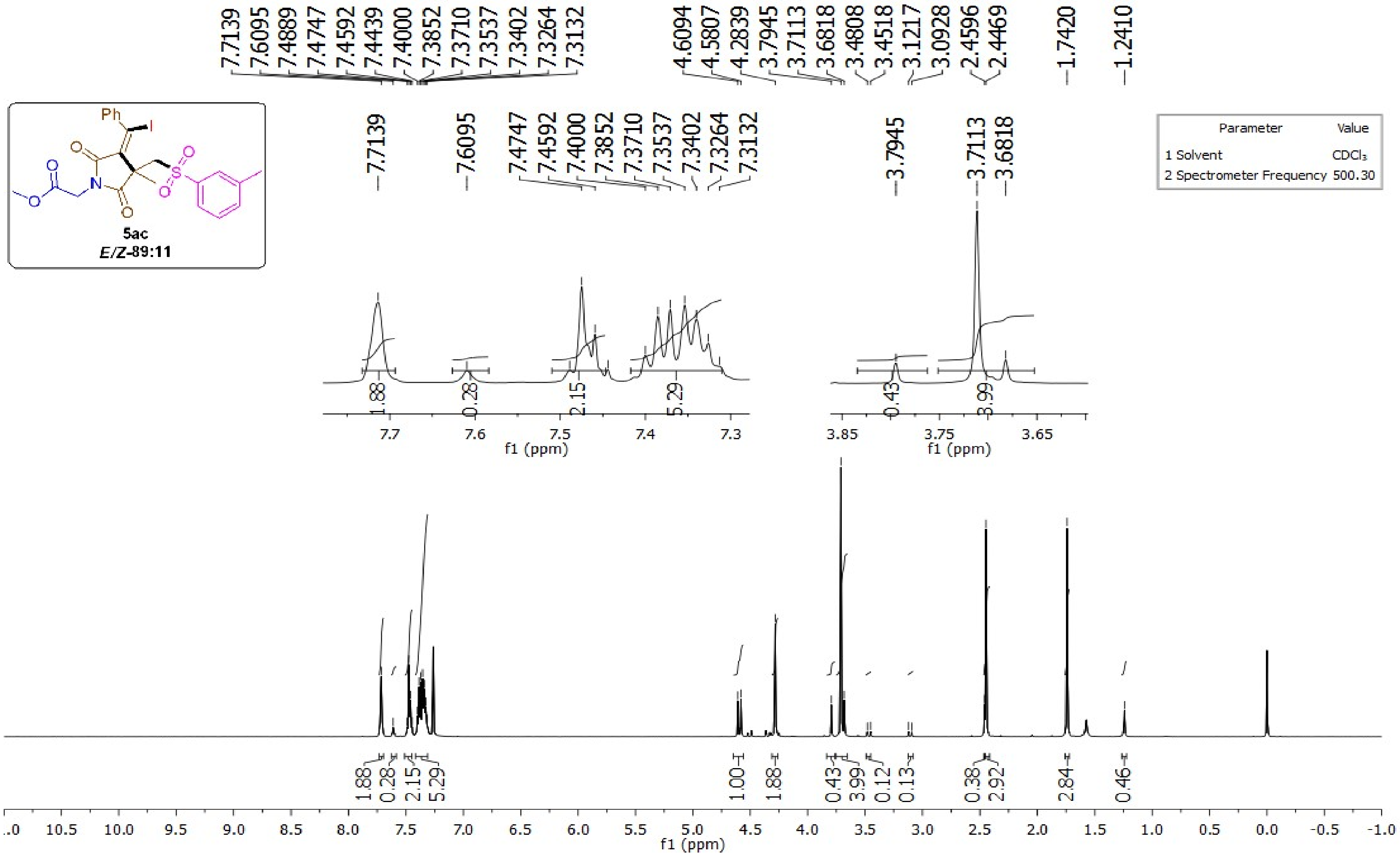

Figure S5. ^{1}H NMR spectra of Methyl 2-(4-(iodo(phenyl)methylene)-3-methyl-2,5-dioxo-3-((*m*-tolylsulphonyl) methyl) pyrrolidin-1-yl)acetate (**5ac**)

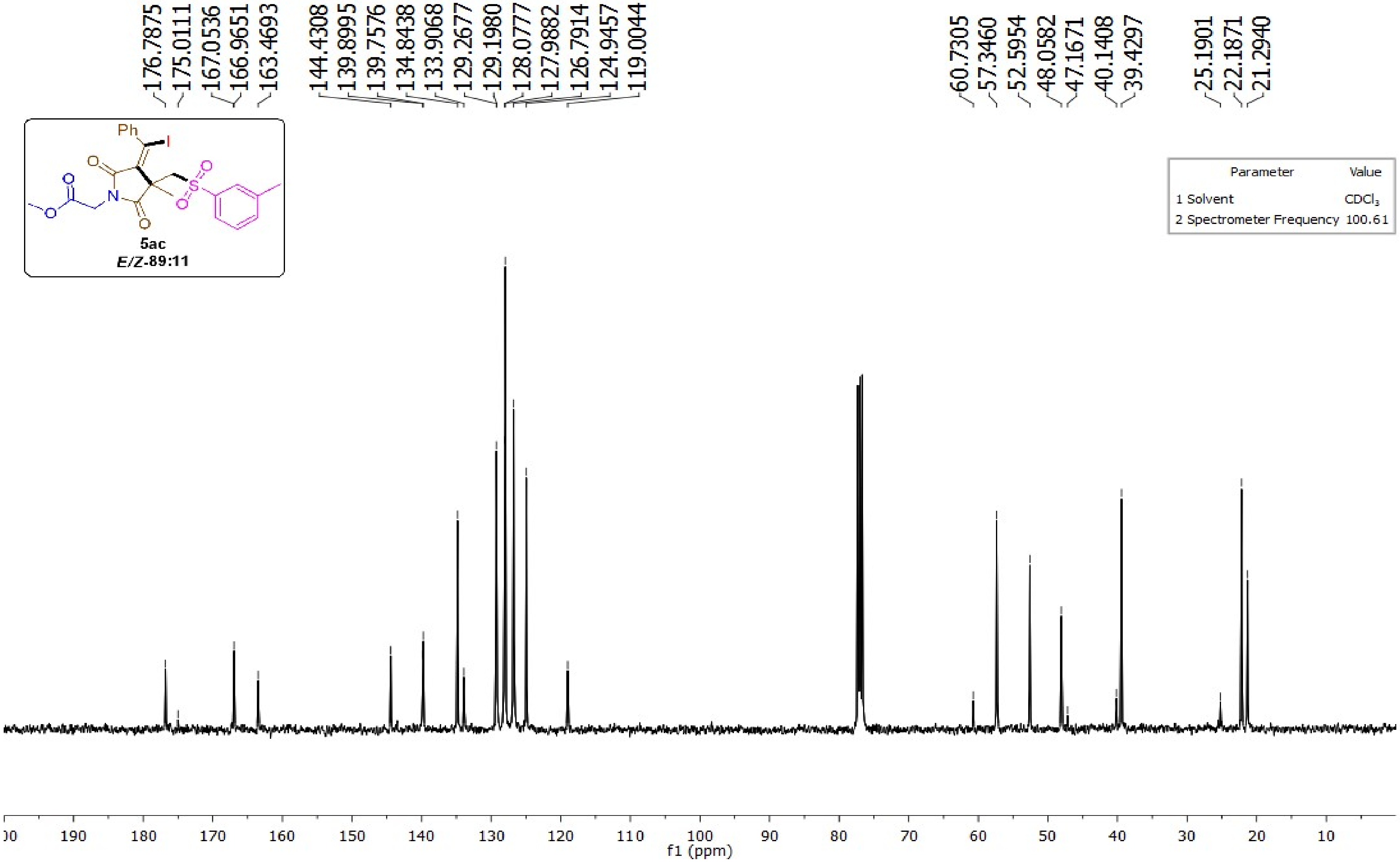

Figure S6. ^{13}C NMR spectra of Methyl 2-(4-(iodo(phenyl)methylene)-3-methyl-2,5-dioxo-3-((*m*-tolylsulphonyl) methyl) pyrrolidin-1-yl)acetate (**5ac**)

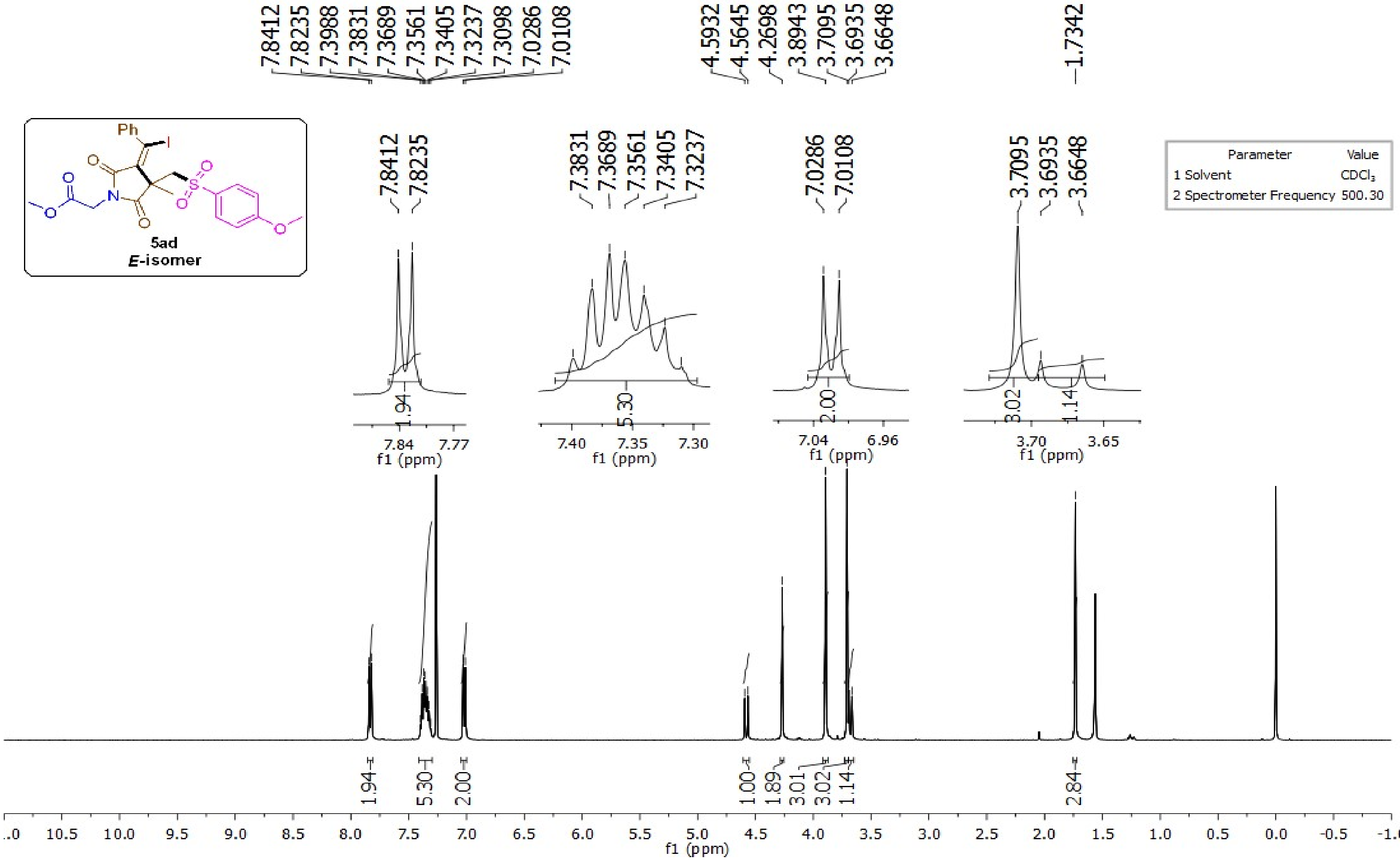

Figure S7. ^{1}H NMR spectra of Methyl (*E*)-2-(4-(iodo(phenyl)methylene)-3-(((4-methoxyphenyl)sulphonyl)methyl)-3-methyl-2,5-dioxopyrrolidin-1-yl)acetate (**5ad**)

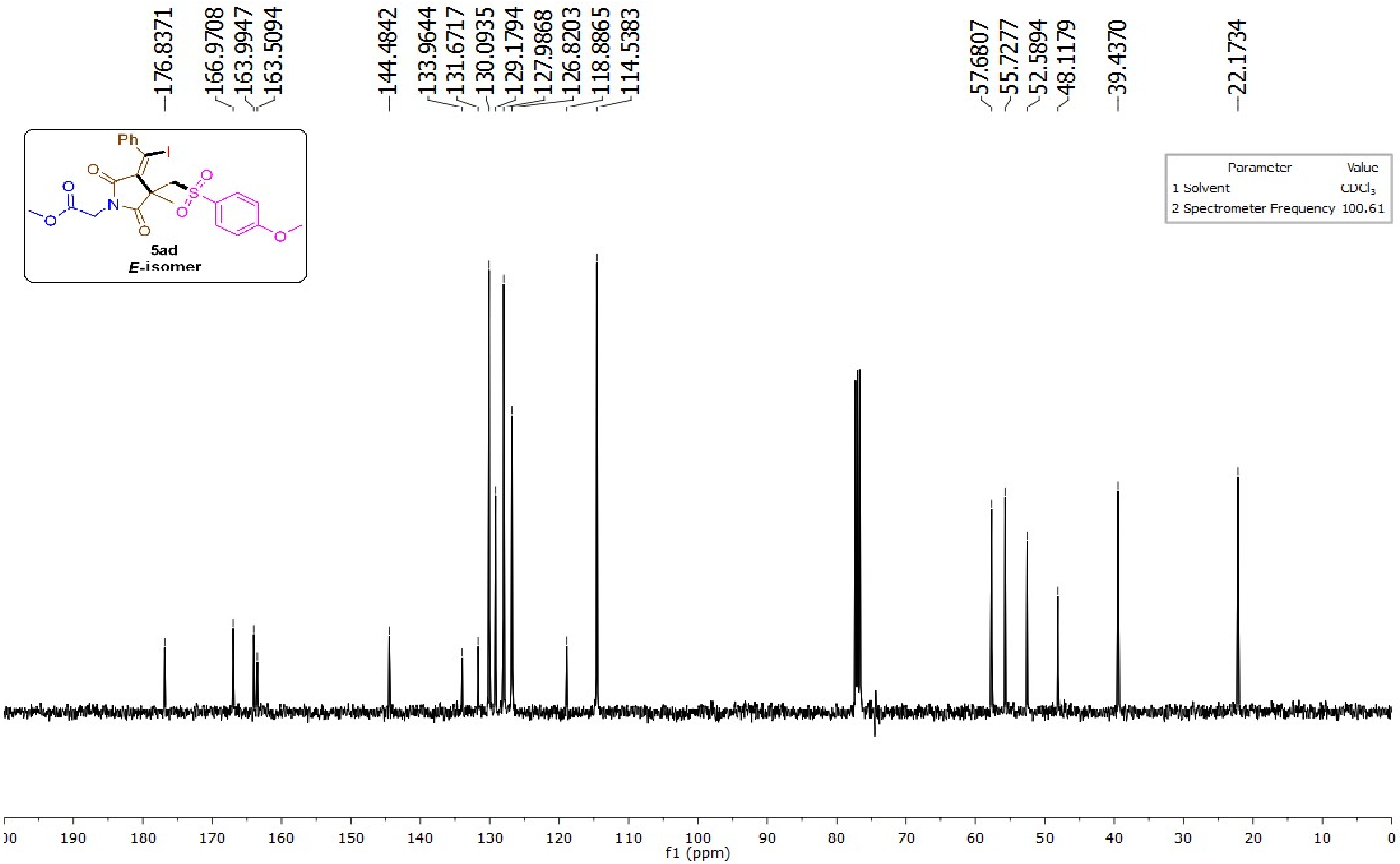

Figure S8. ^{13}C NMR spectra of Methyl (*E*)-2-(4-(iodo(phenyl)methylene)-3-(((4-methoxyphenyl)sulphonyl)methyl)-3-methyl-2,5-dioxopyrrolidin-1-yl)acetate (**5ad**)

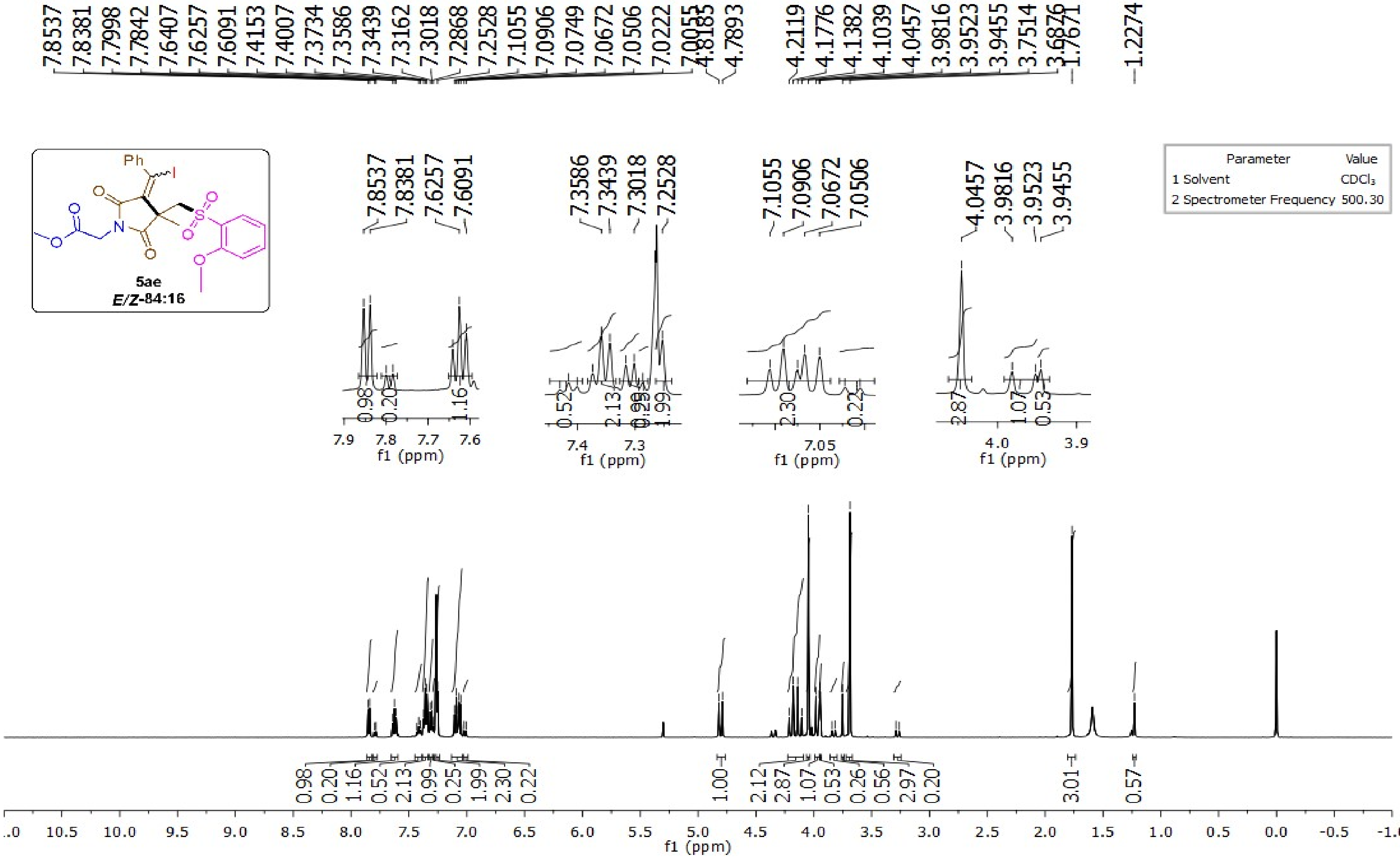

Figure S9. ^{1}H NMR spectra of Methyl 2-(4-(iodo(phenyl)methylene)-3-(((2-methoxyphenyl)sulphonyl)methyl)-3-methyl-2,5-dioxopyrrolidin-1-yl)acetate (**5ae**)

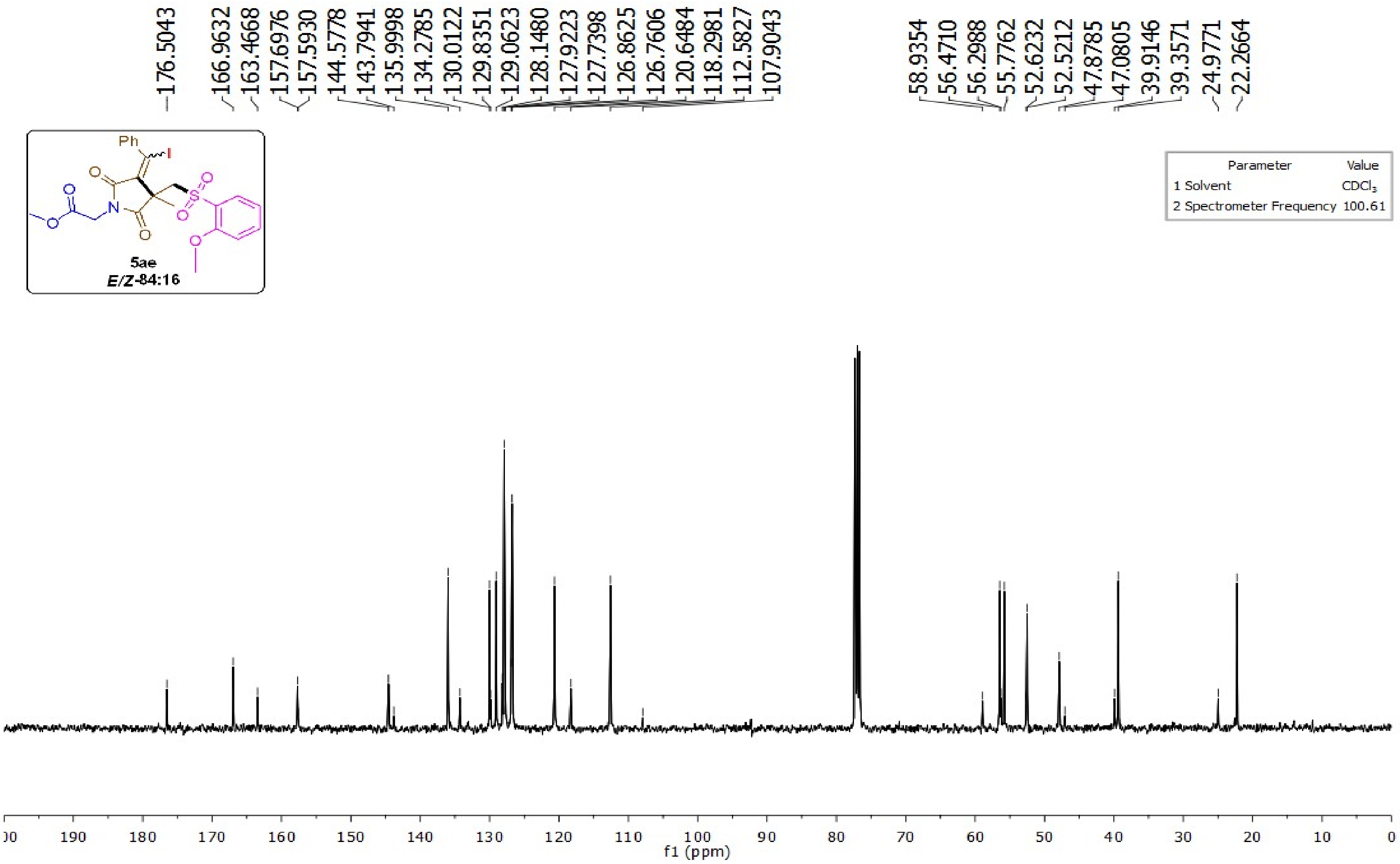

Figure S10. ^{13}C NMR spectra of Methyl 2-(4-(iodo(phenyl)methylene)-3-(((2-methoxyphenyl)sulphonyl)methyl)-3-methyl-2,5-dioxopyrrolidin-1-yl)acetate (**5ae**)

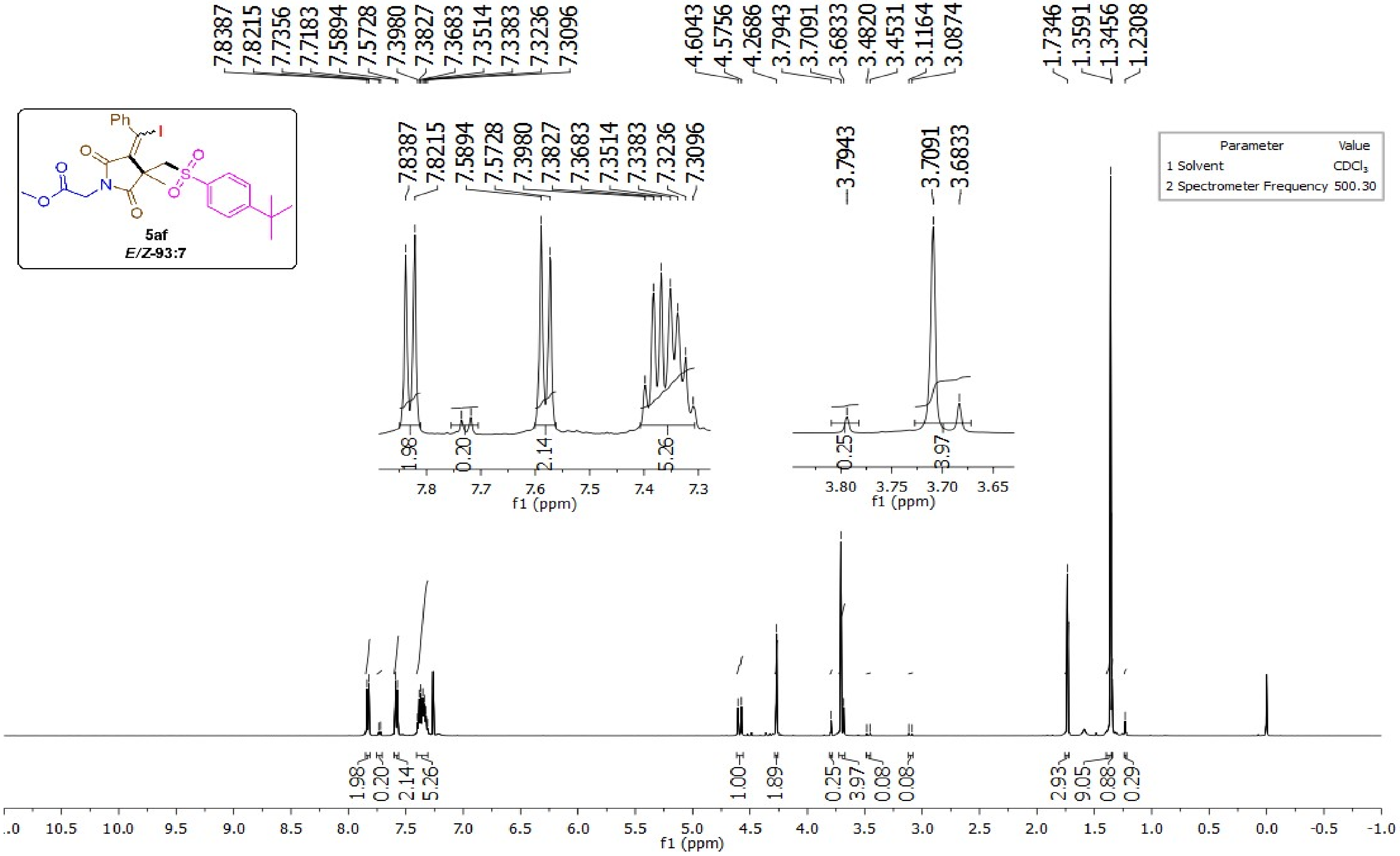

Figure S11. ^{1}H NMR spectra of Methyl 2-(3-(((4-(tert-butyl)phenyl)sulphonyl)methyl)-4-(iodo(phenyl)methylene)-3-methyl -2,5-dioxopyrrolidin-1-yl)acetate (**5af**)

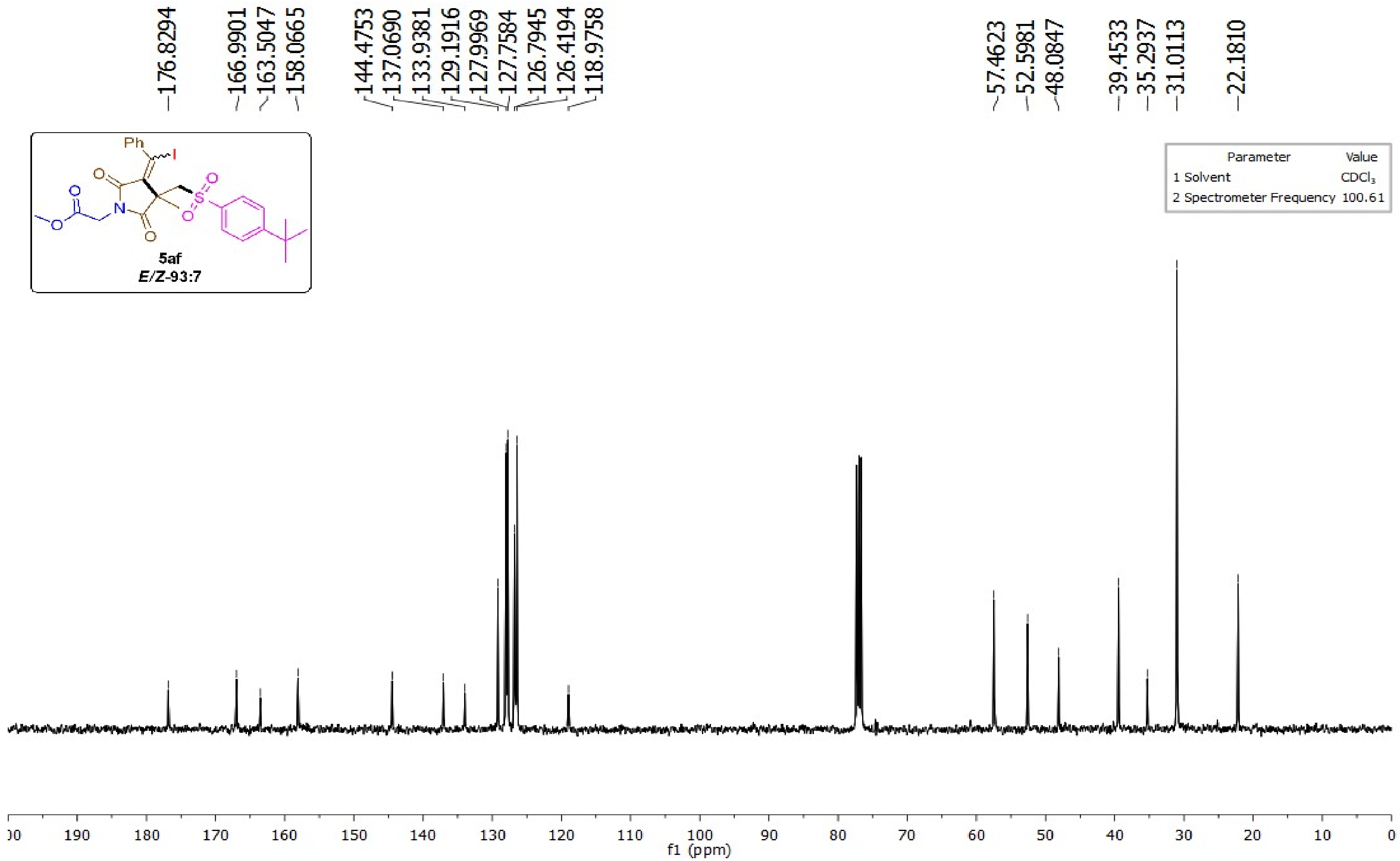

Figure S12. ^{13}C NMR spectra of Methyl 2-(3-(((4-(tert-butyl)phenyl)sulphonyl)methyl)-4-(iodo(phenyl)methylene)-3-methyl -2,5-dioxopyrrolidin-1-yl)acetate (**5af**)

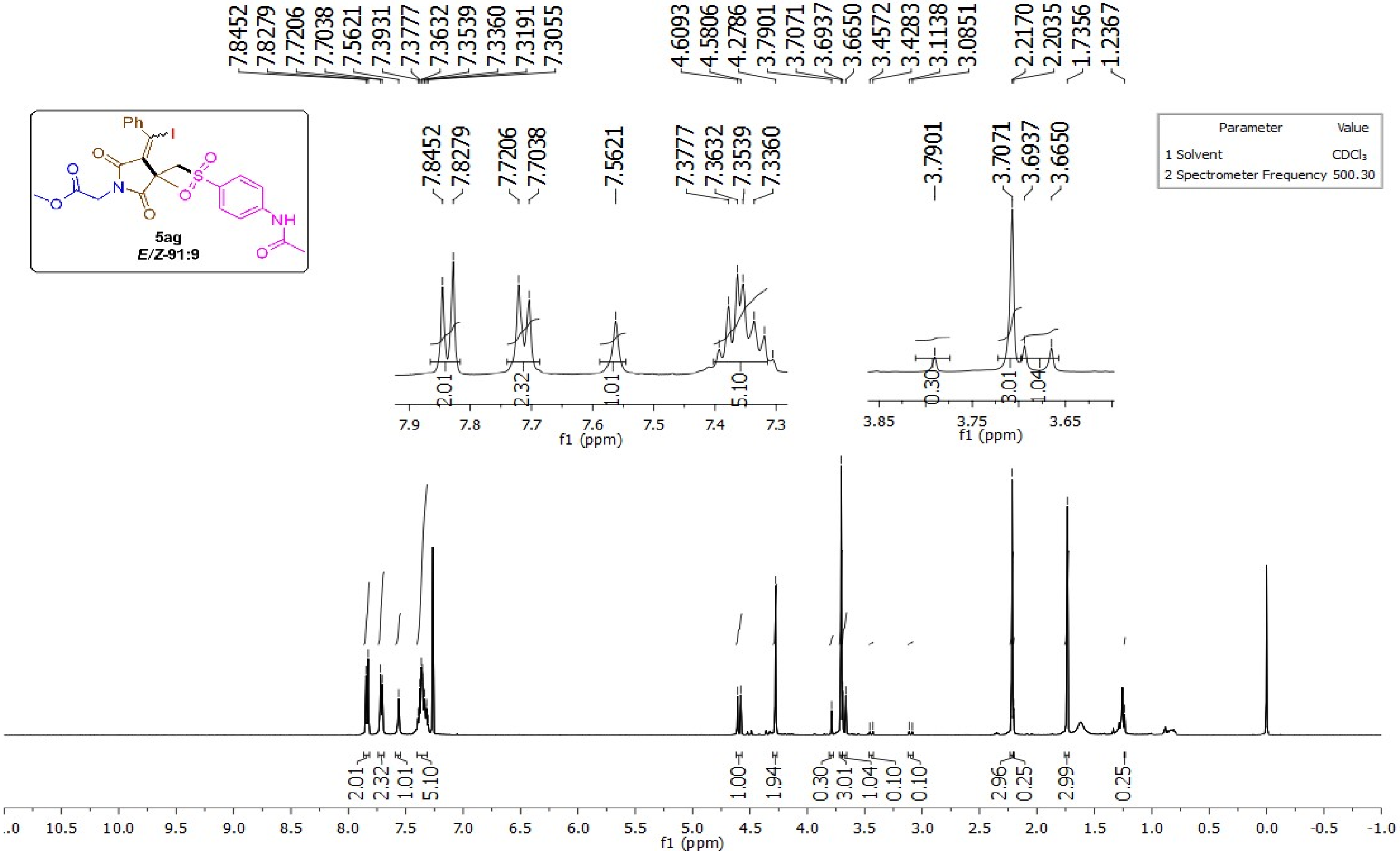

Figure S13. ^{1}H NMR spectra of Methyl 2-(3-(((4-acetamidophenyl)sulphonyl)methyl)-4-(iodo(phenyl)methylene)-3-methyl-2,5-dioxopyrrolidin-1-yl)acetate (**5ag**)

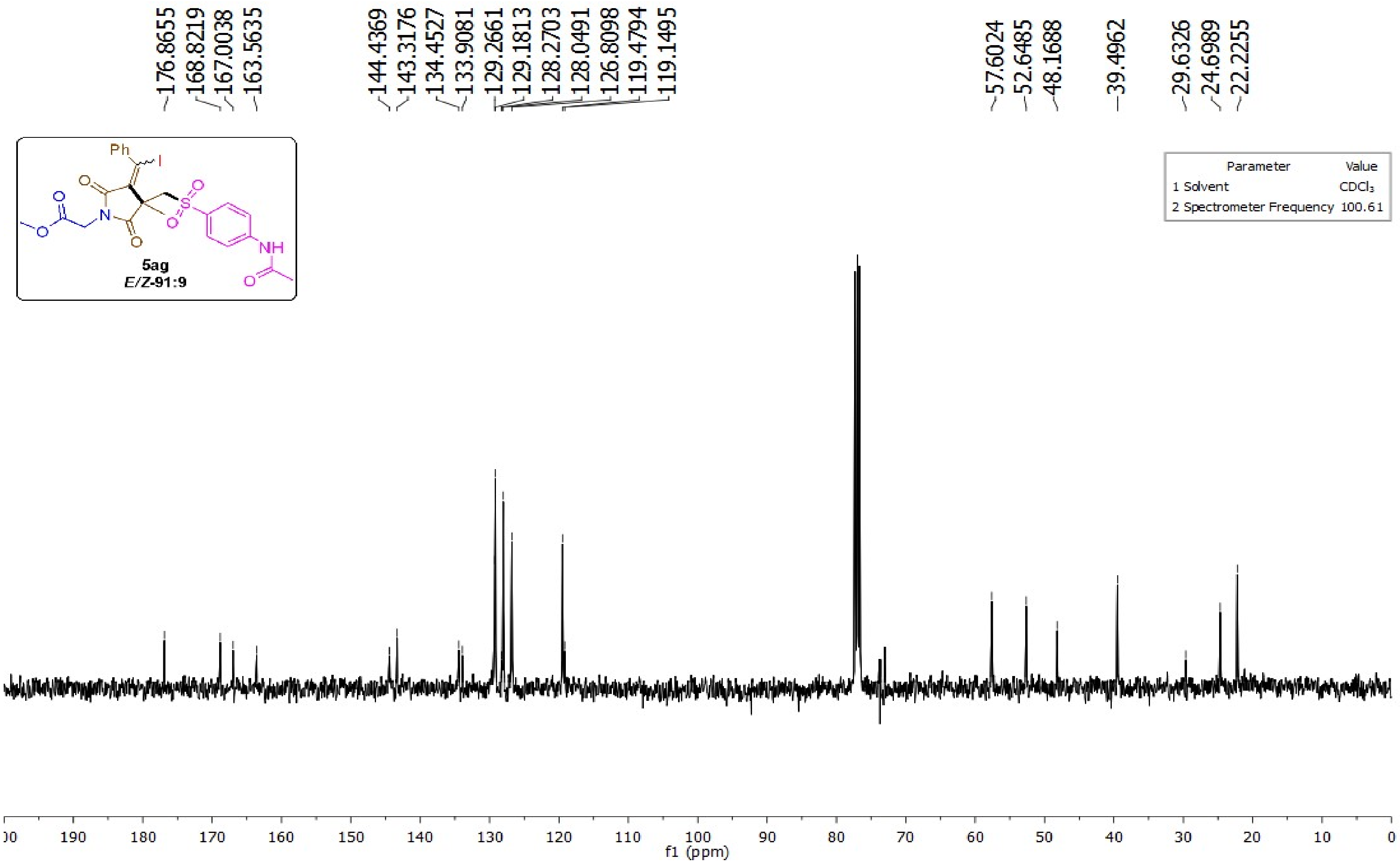

Figure S14. ^{13}C NMR spectra of Methyl 2-(3-(((4-acetamidophenyl)sulphonyl)methyl)-4-(iodo(phenyl)methylene)-3-methyl-2,5-dioxopyrrolidin-1-yl)acetate (**5ag**)

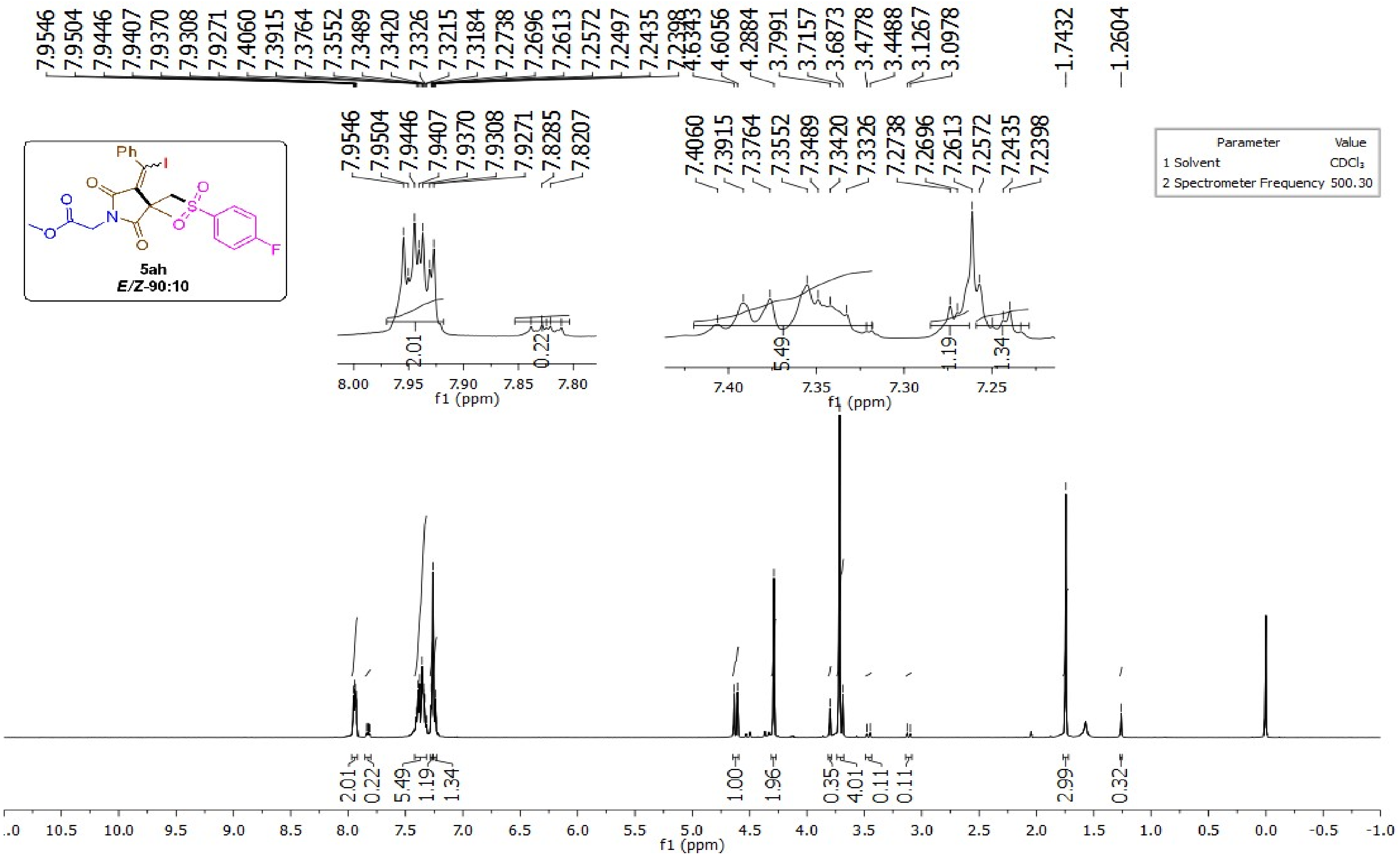

Figure S15. ^{1}H NMR spectra of Methyl 2-(3-(((4-fluorophenyl)sulphonyl)methyl)-4-(iodo(phenyl)methylene)-3-methyl-2,5-dioxopyrrolidin-1-yl)acetate (**5ah**)

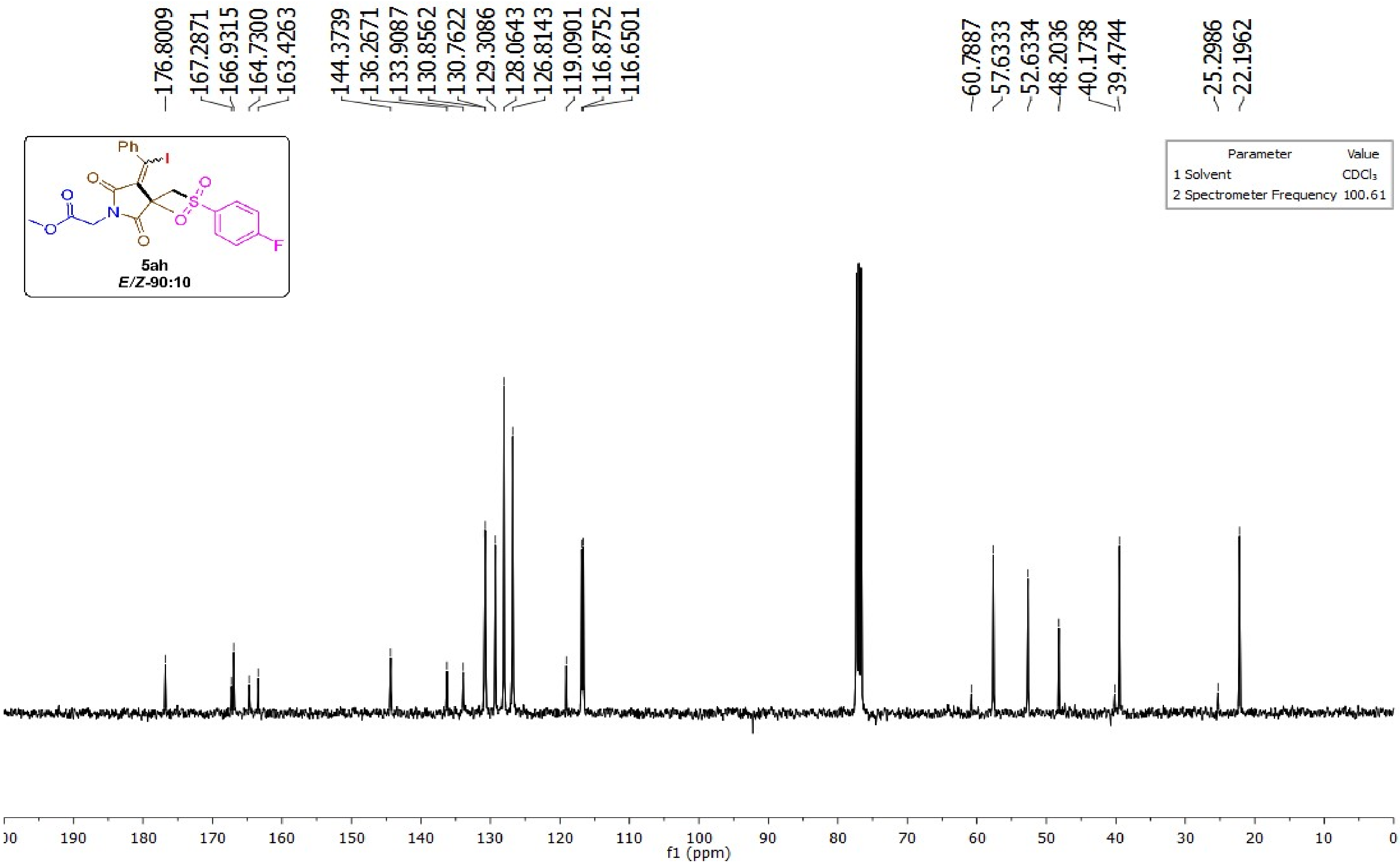

Figure S16. ^{13}C NMR spectra of Methyl 2-(3-(((4-fluorophenyl)sulphonyl)methyl)-4-(iodo(phenyl)methylene)-3-methyl-2,5-dioxopyrrolidin-1-yl)acetate (**5ah**)

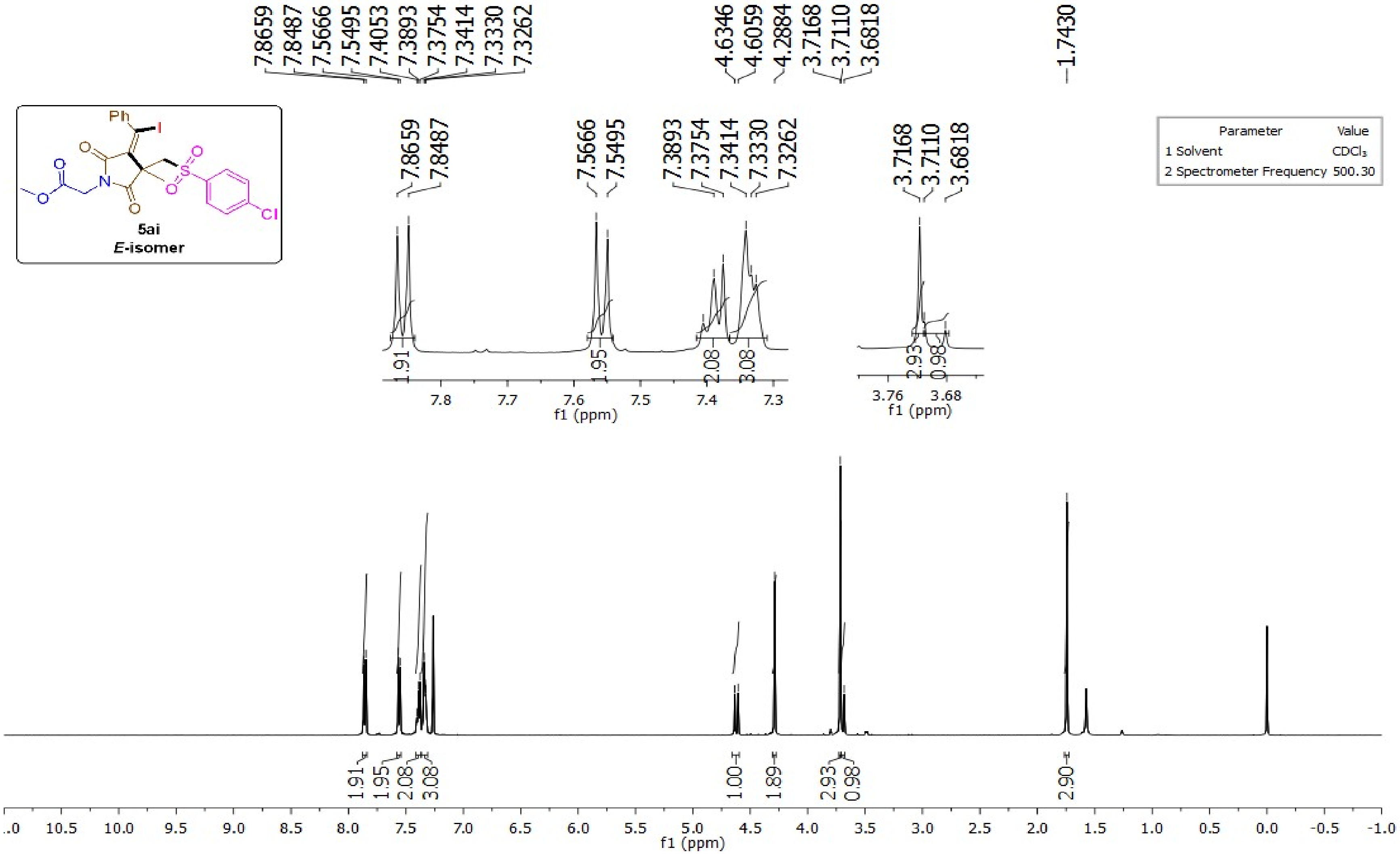

Figure S17. ^{1}H NMR spectra of Methyl (*E*)-2-(3-(((4-chlorophenyl)sulphonyl)methyl)-4-(iodo(phenyl)methylene)-3-methyl-2,5-dioxopyrrolidin-1-yl)acetate (**5ai**)

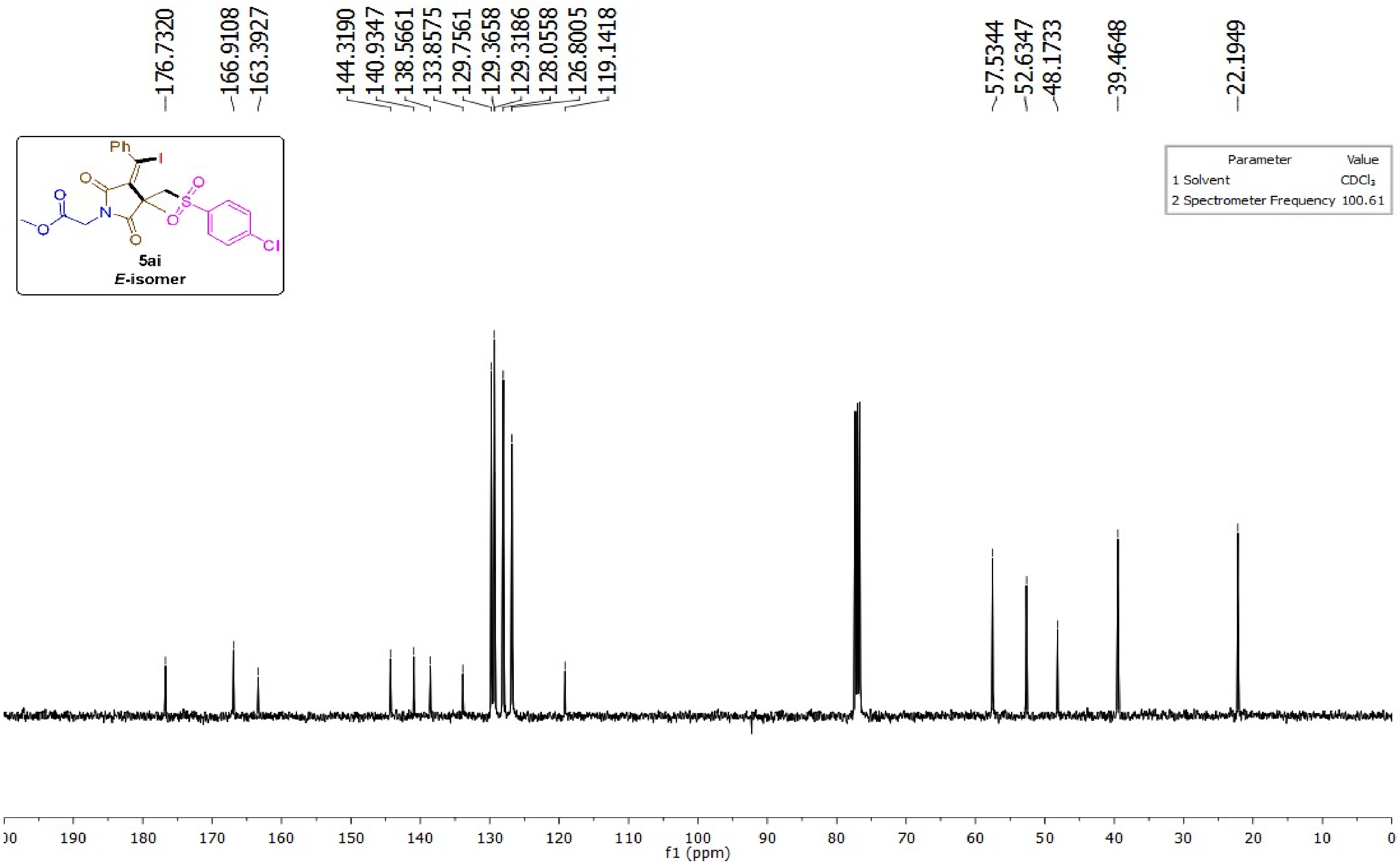

Figure S18. ^{13}C NMR spectra of Methyl (*E*)-2-(3-(((4-chlorophenyl)sulphonyl)methyl)-4-(iodo(phenyl)methylene)-3-methyl -2,5-dioxopyrrolidin-1-yl)acetate (**5ai**)

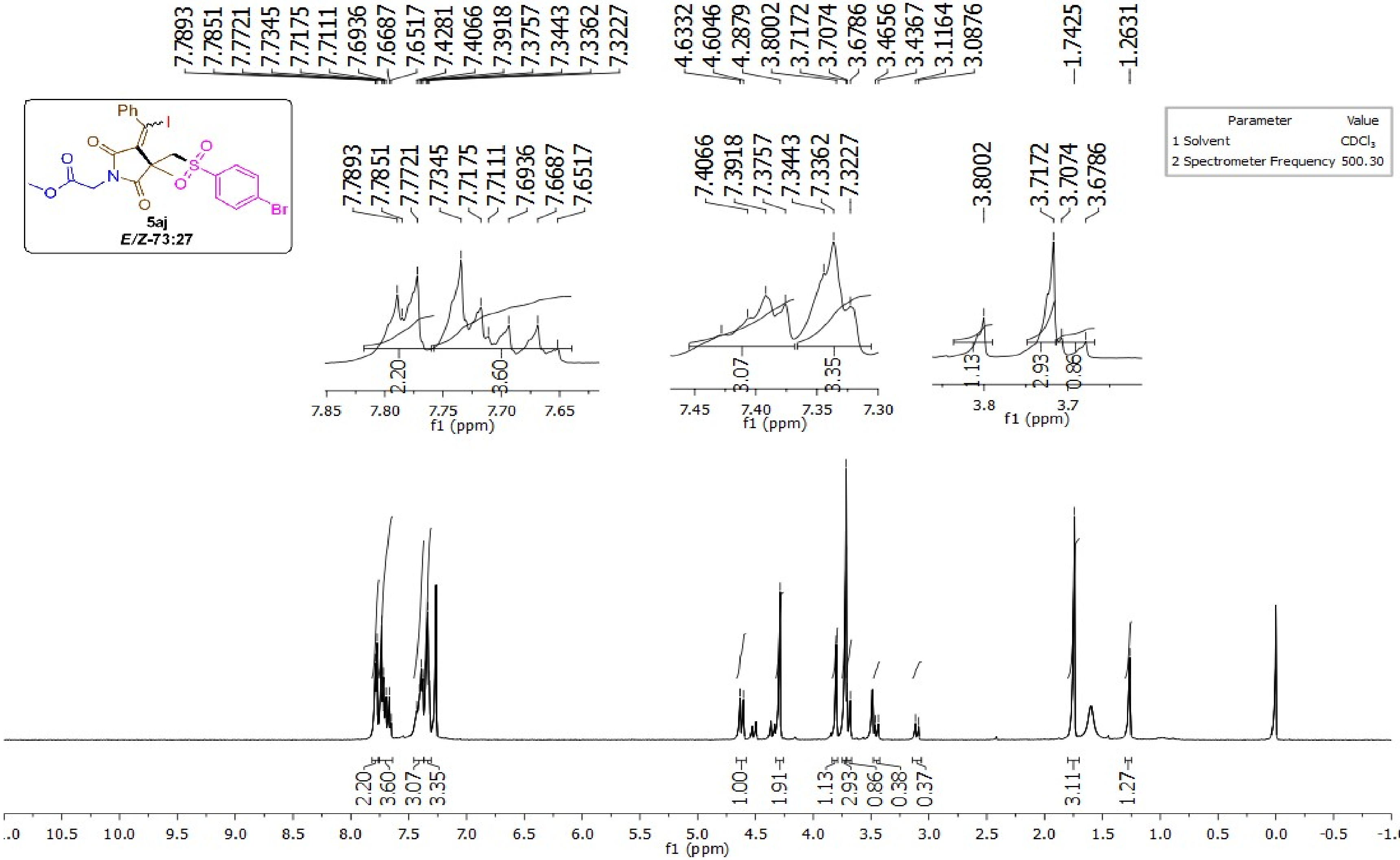

Figure S19. ^{1}H NMR spectra of Methyl 2-(3-(((4-bromophenyl)sulphonyl)methyl)-4-(iodo(phenyl)methylene)-3-methyl-2,5 -dioxopyrrolidin-1-yl)acetate (**5aj**)

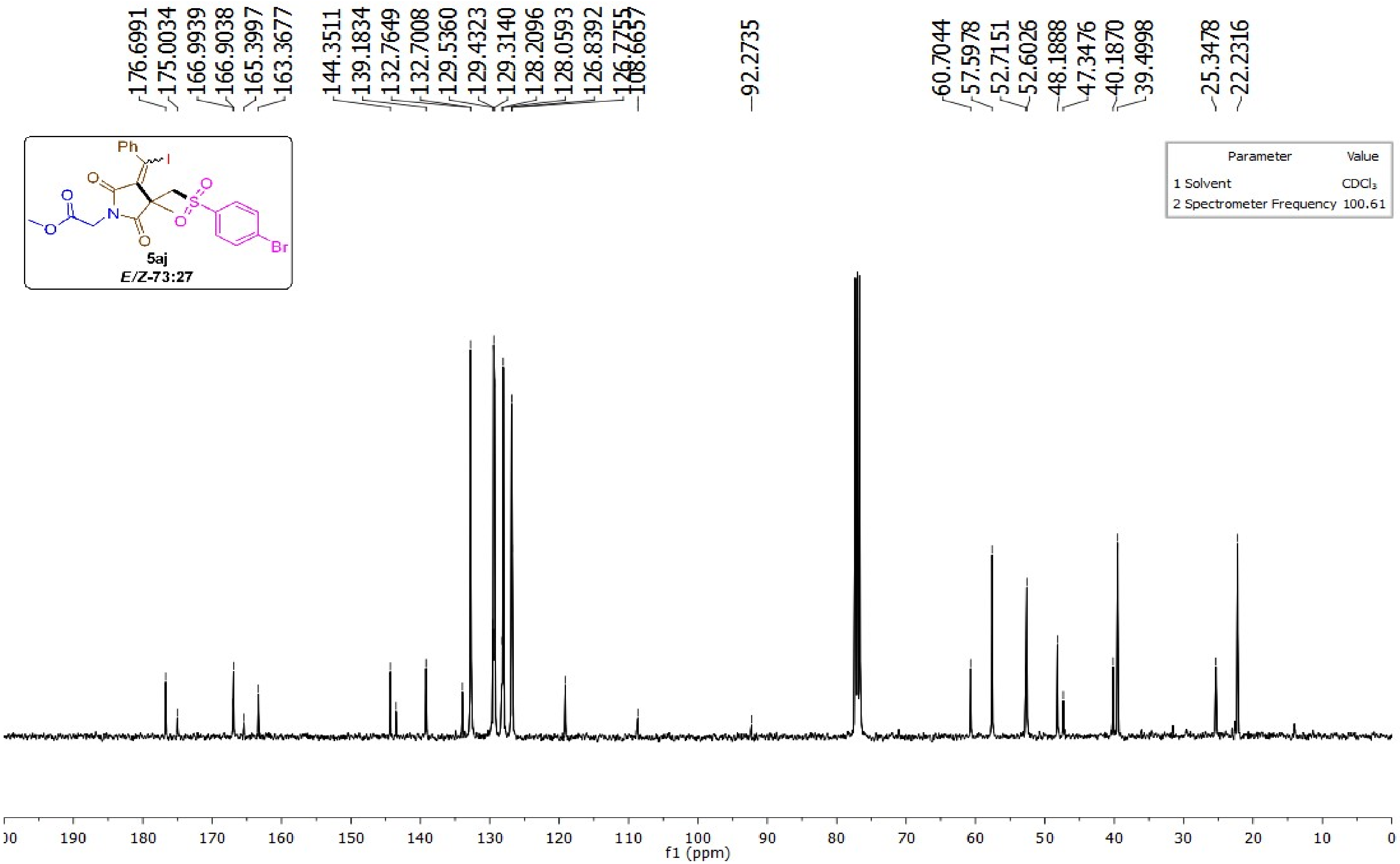

Figure S20. ^{13}C NMR spectra of Methyl 2-(3-(((4-bromophenyl)sulphonyl)methyl)-4-(iodo(phenyl)methylene)-3-methyl-2,5 -dioxopyrrolidin-1-yl)acetate (**5aj**)

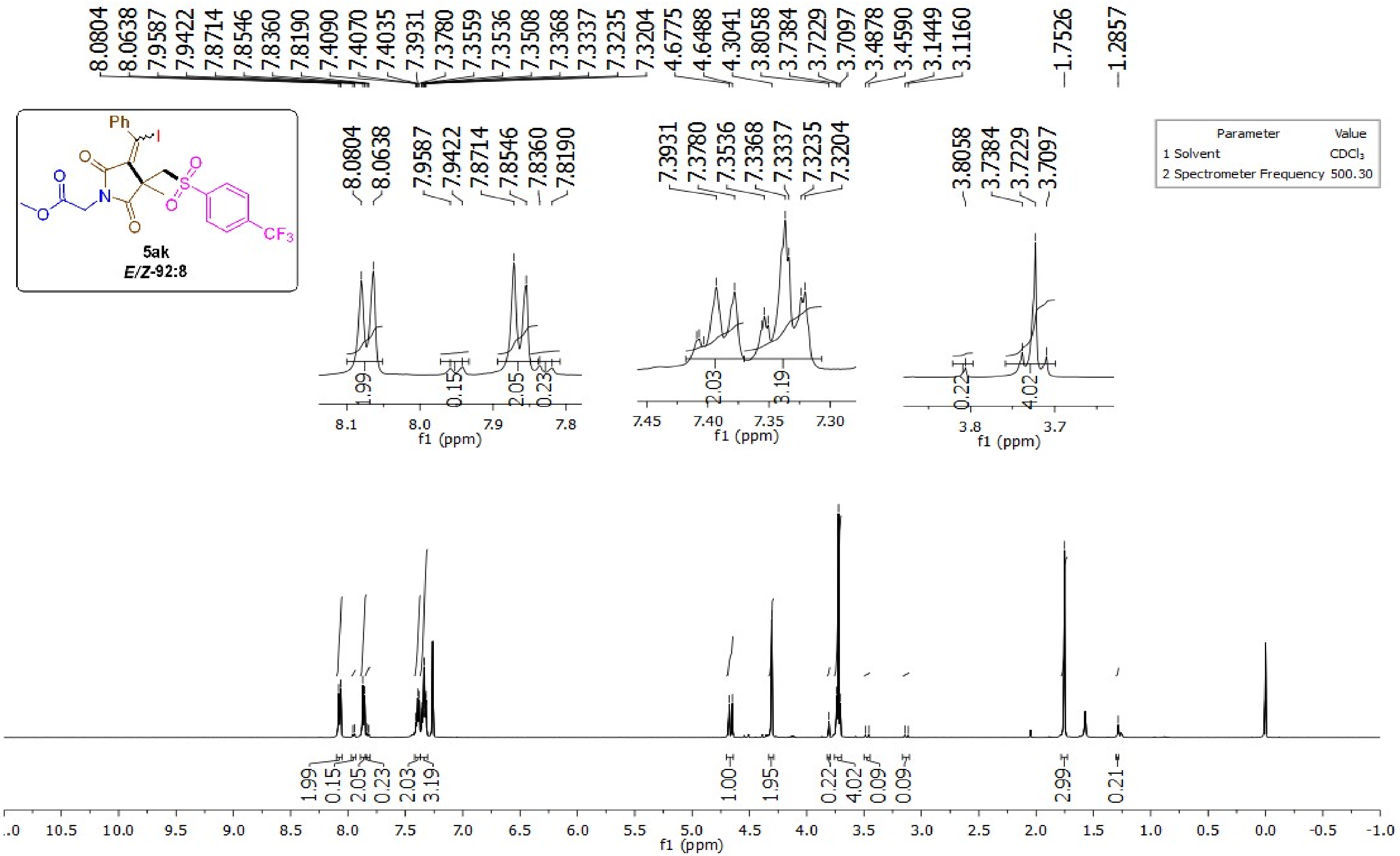

Figure S21. ^{1}H NMR spectra of Methyl 2-(4-(iodo(phenyl)methylene)-3-methyl-2,5-dioxo-3-(((4-(trifluoromethyl)phenyl) sulphonyl)methyl)pyrrolidin-1-yl)acetate (**5ak**)

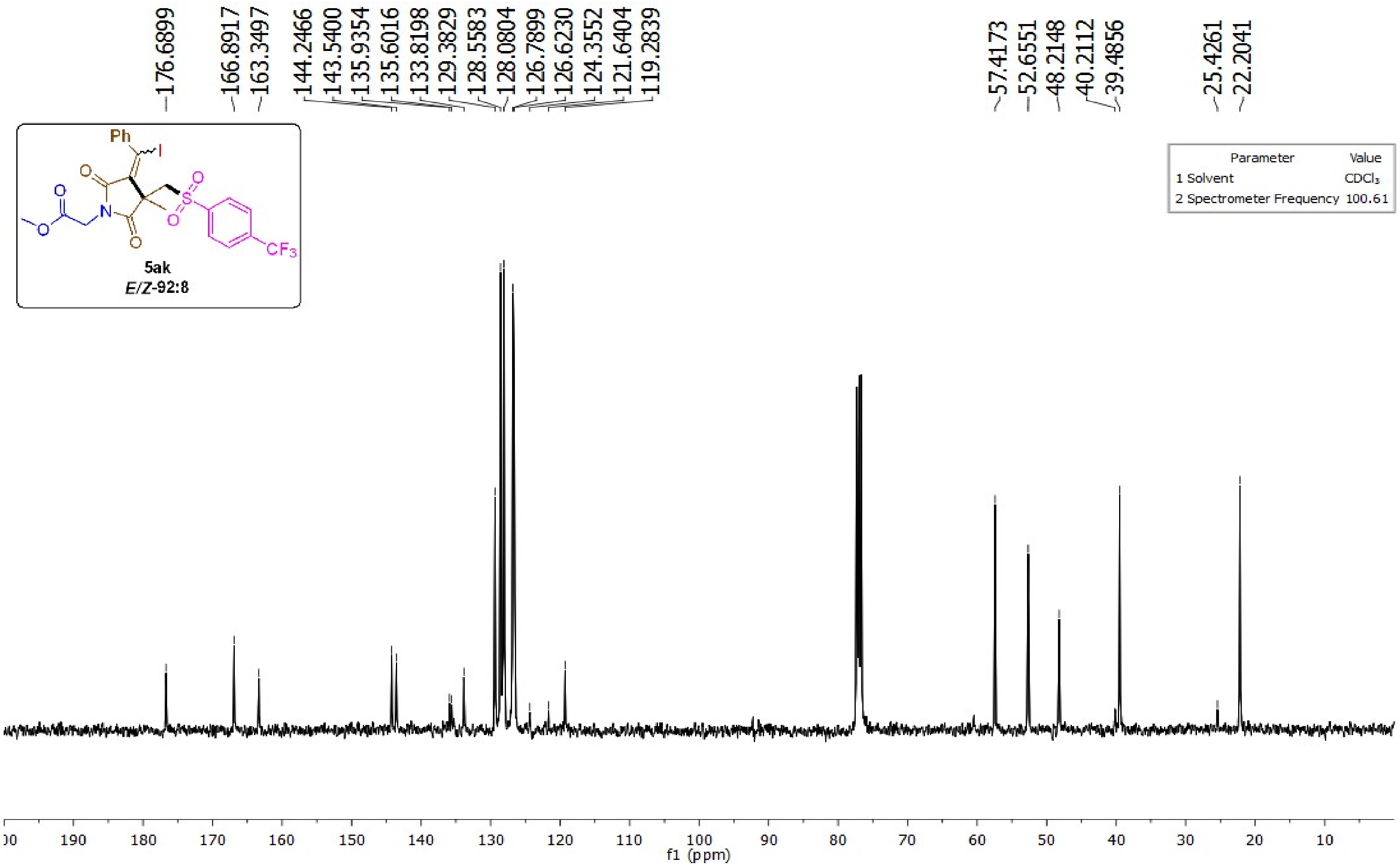

Figure S22. ^{13}C NMR spectra of Methyl 2-(4-(iodo(phenyl)methylene)-3-methyl-2,5-dioxo-3-(((4-(trifluoromethyl)phenyl) sulphonyl)methyl)pyrrolidin-1-yl)acetate (**5ak**)

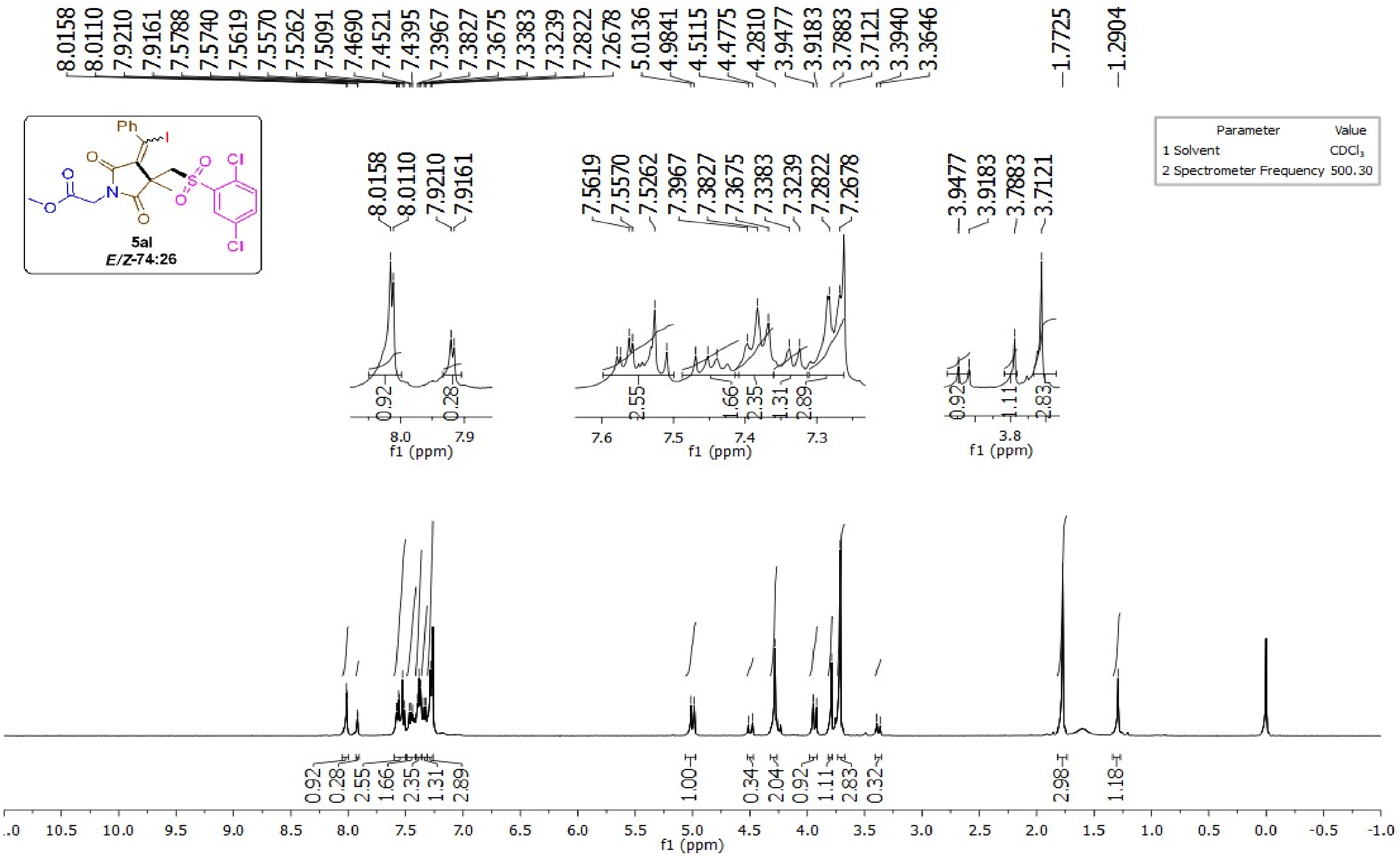

Figure S23. ^{1}H NMR spectra of Methyl 2-(3-(((2,5-dichlorophenyl)sulphonyl)methyl)-4-(iodo(phenyl)methylene)-3-methyl-2,5-dioxopyrrolidin-1-yl)acetate (**5al**)

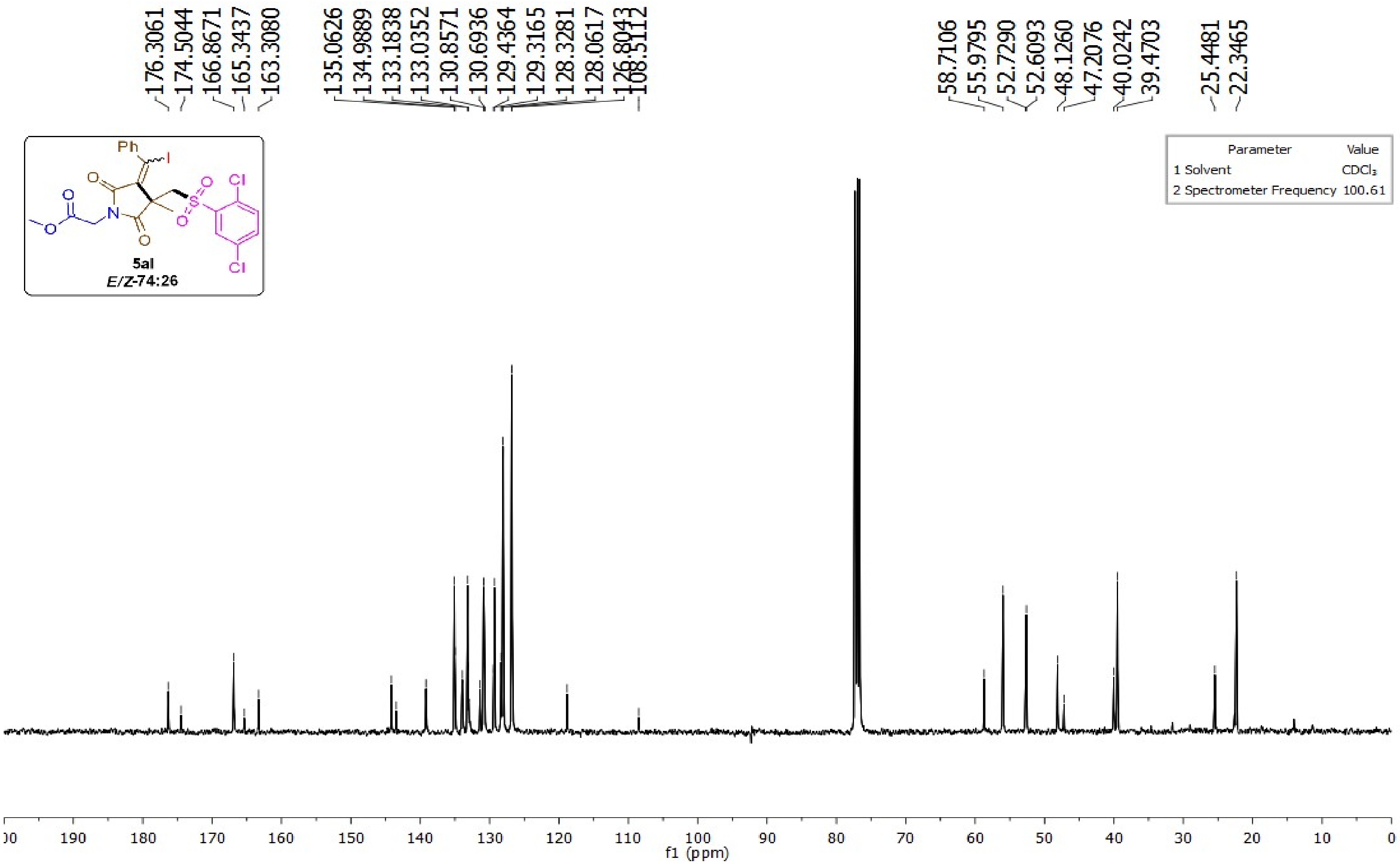

Figure S24. ^{13}C NMR spectra ofMethyl 2-(3-(((2,5-dichlorophenyl)sulphonyl)methyl)-4-(iodo(phenyl)methylene)-3-methyl-2,5-dioxopyrrolidin-1-yl)acetate (**5al**)

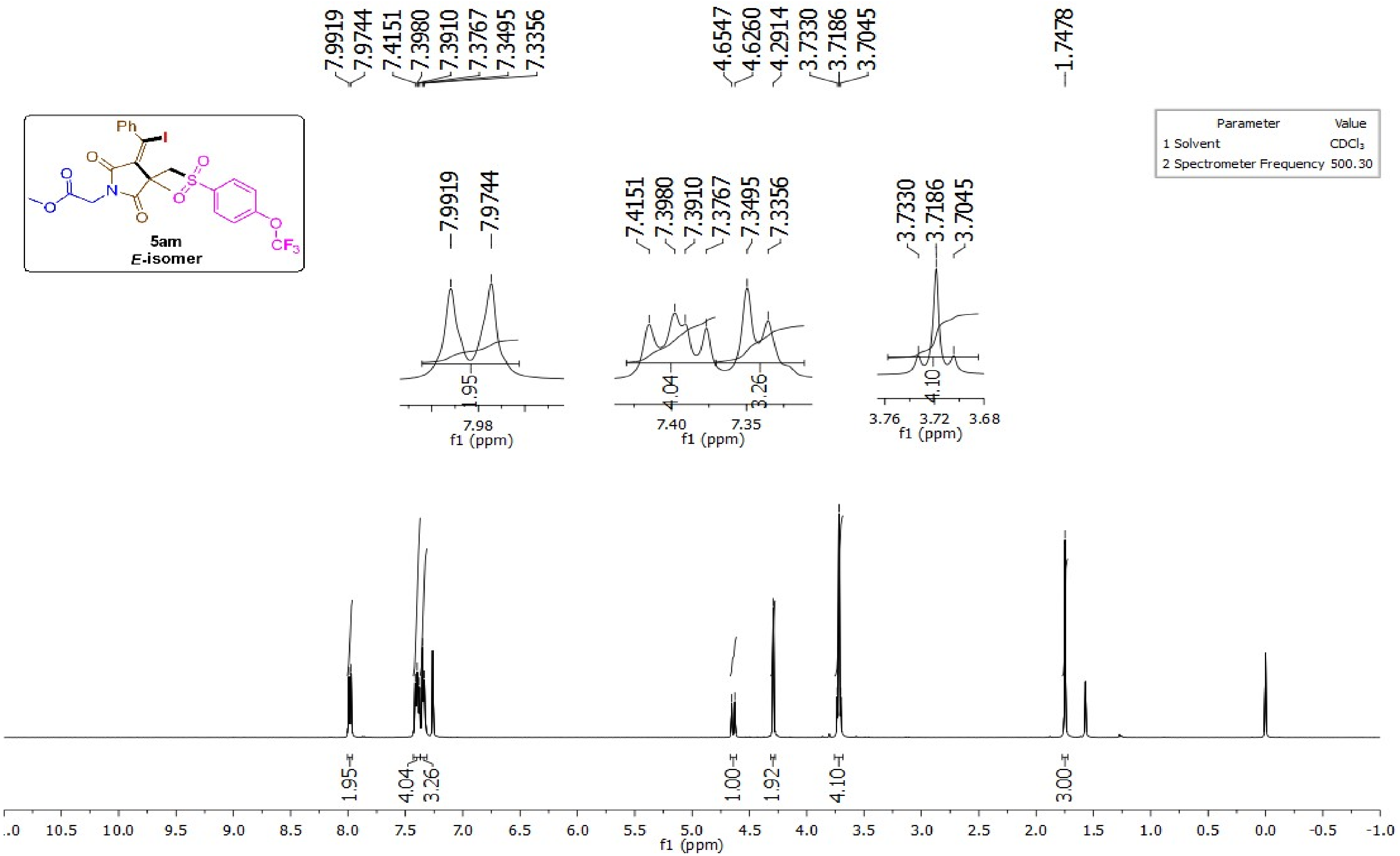

Figure S25. ^{1}H NMR spectra of Methyl (*E*)-2-(4-(iodo(phenyl)methylene)-3-methyl-2,5-dioxo-3-(((4-(trifluoromethoxy) phenyl)sulphonyl)methyl)pyrrolidin-1-yl)acetate (**5am**)

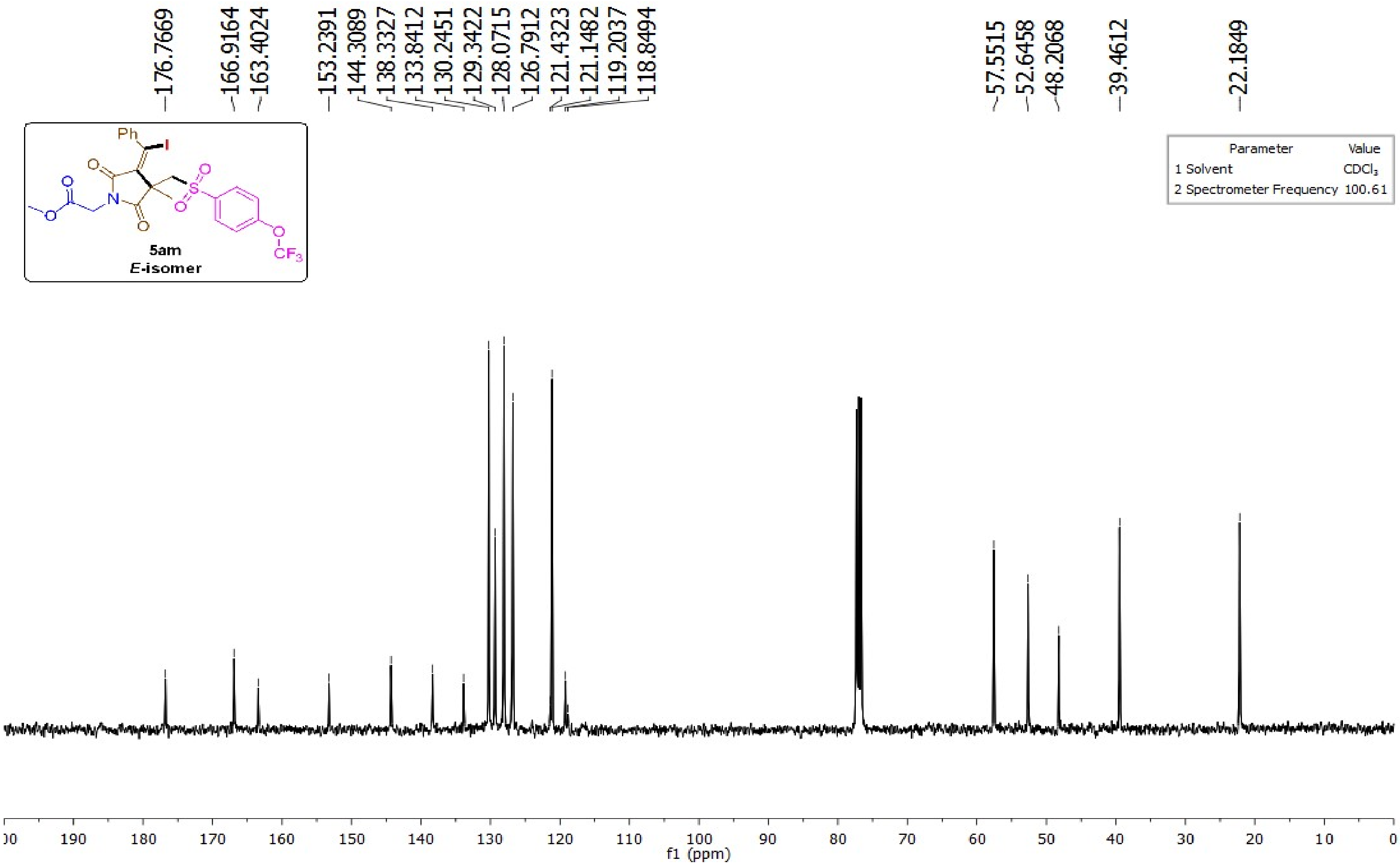

Figure S26. ^{13}C NMR spectra of Methyl (*E*)-2-(4-(iodo(phenyl)methylene)-3-methyl-2,5-dioxo-3-(((4-(trifluoromethoxy) phenyl)sulphonyl)methyl)pyrrolidin-1-yl)acetate (**5am**)

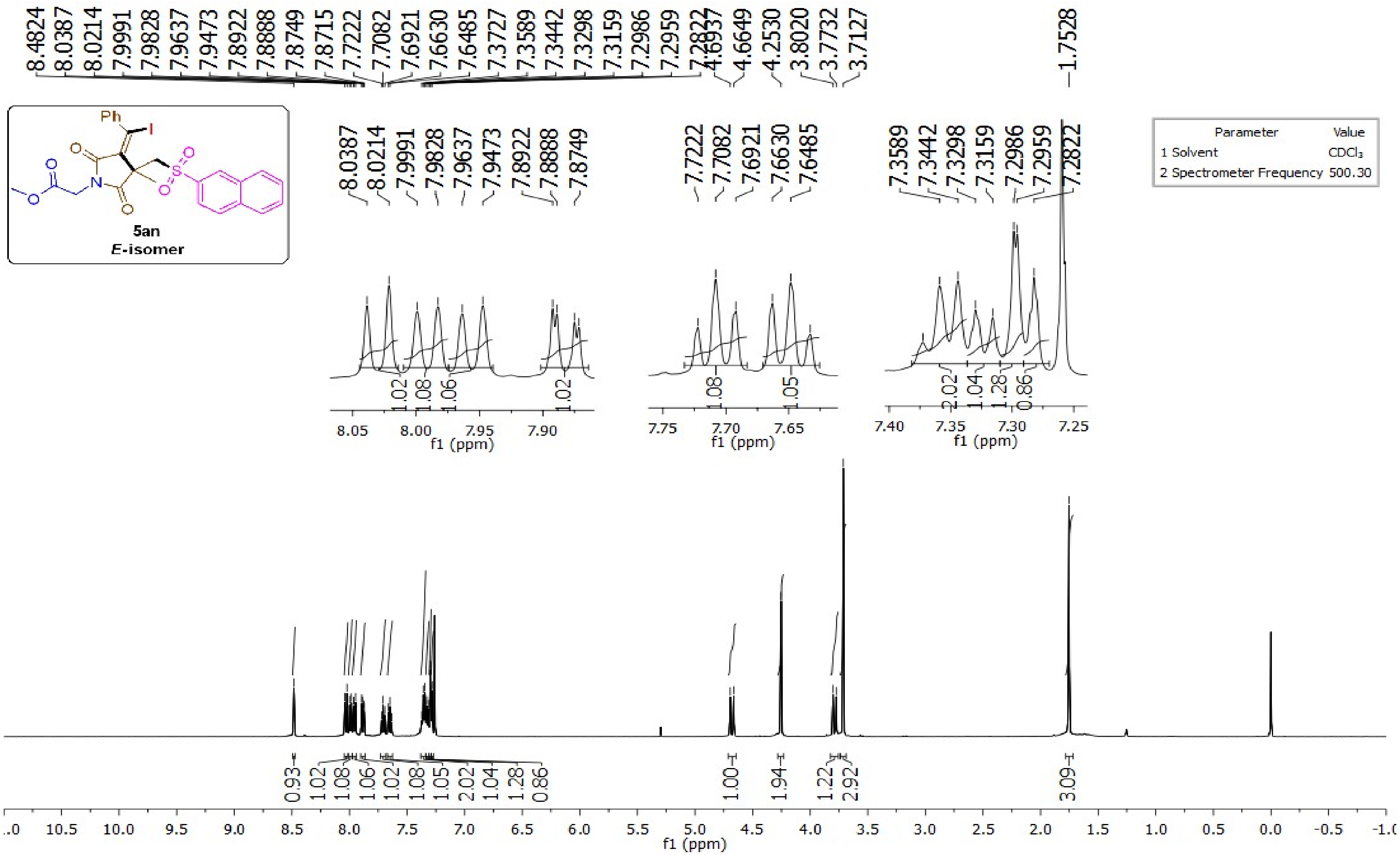

Figure S27. ^{1}H NMR spectra of Methyl (*E*)-2-(4-(iodo(phenyl)methylene)-3-methyl-3-((naphthalen-2-ylsulphonyl)methyl)-2,5-dioxopyrrolidin-1-yl)acetate (**5an**)

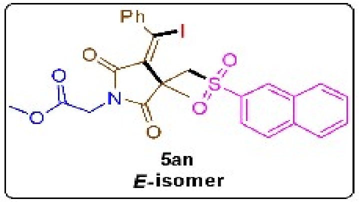

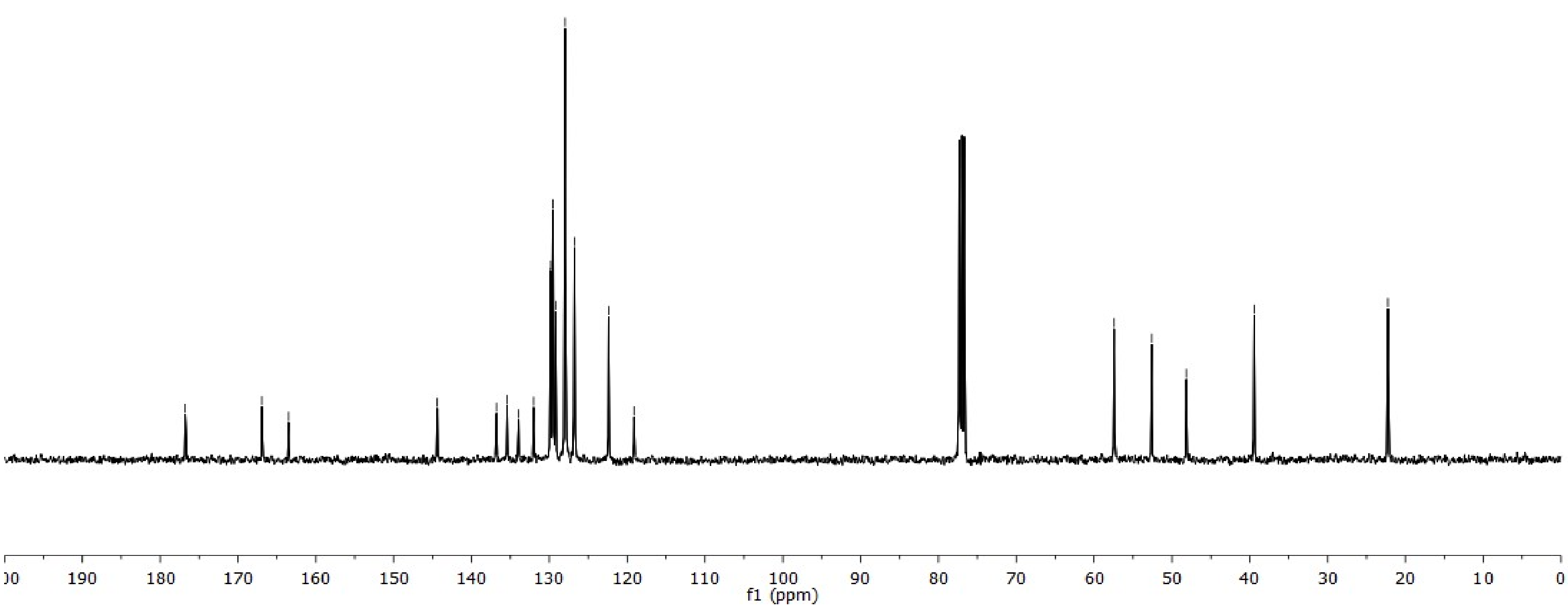

Figure S28. ^{13}C NMR spectra of Methyl (*E*)-2-(4-(iodo(phenyl)methylene)-3-methyl-3-((naphthalen-2-ylsulphonyl)methyl)-2,5-dioxopyrrolidin-1-yl)acetate (**5an**)

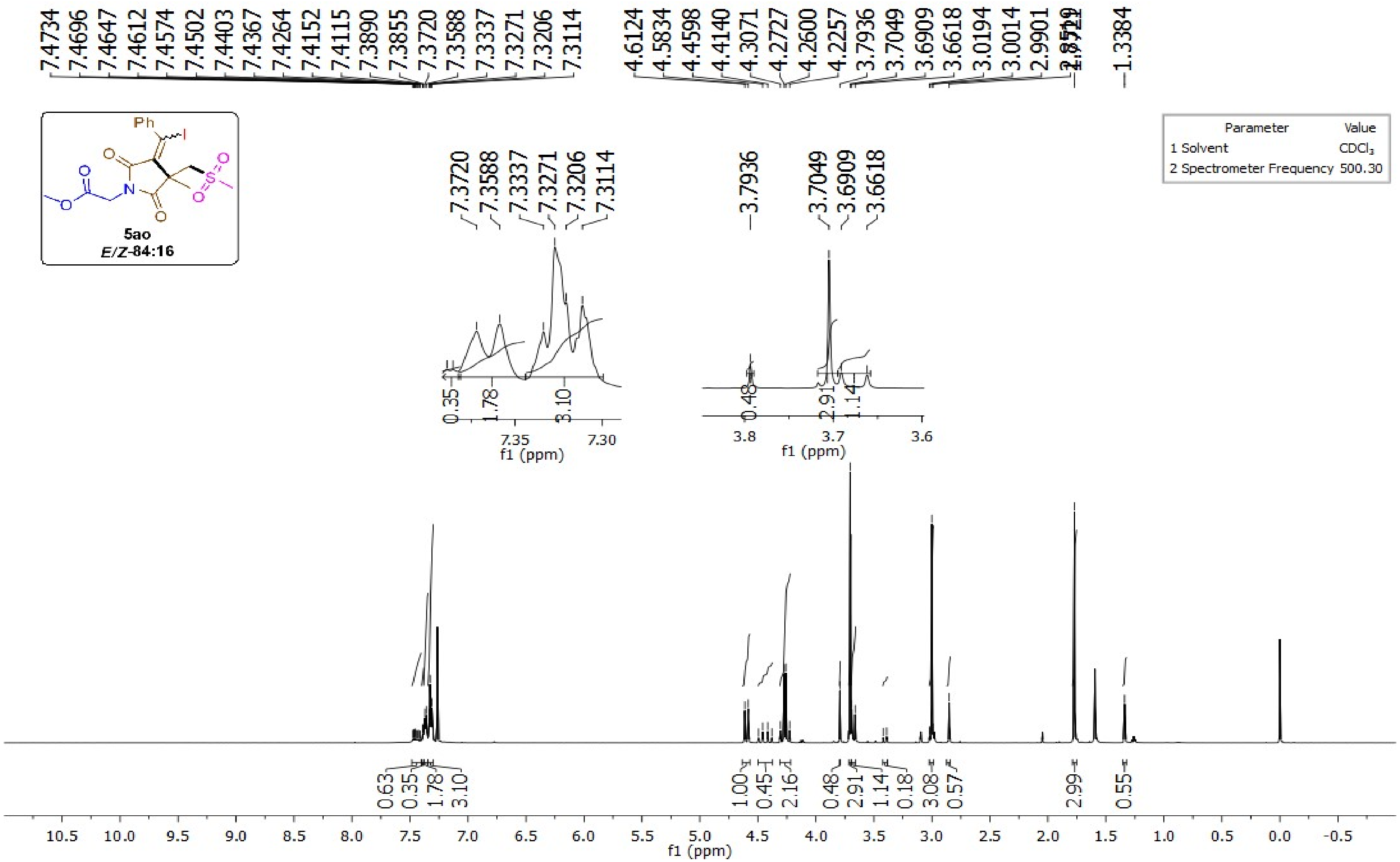

Figure S29. ^{1}H NMR spectra of Methyl 2-(4-(iodo(phenyl)methylene)-3-methyl-3-((methylsulphonyl)methyl)-2,5-dioxo pyrrolidin-1-yl)acetate (**5ao**)

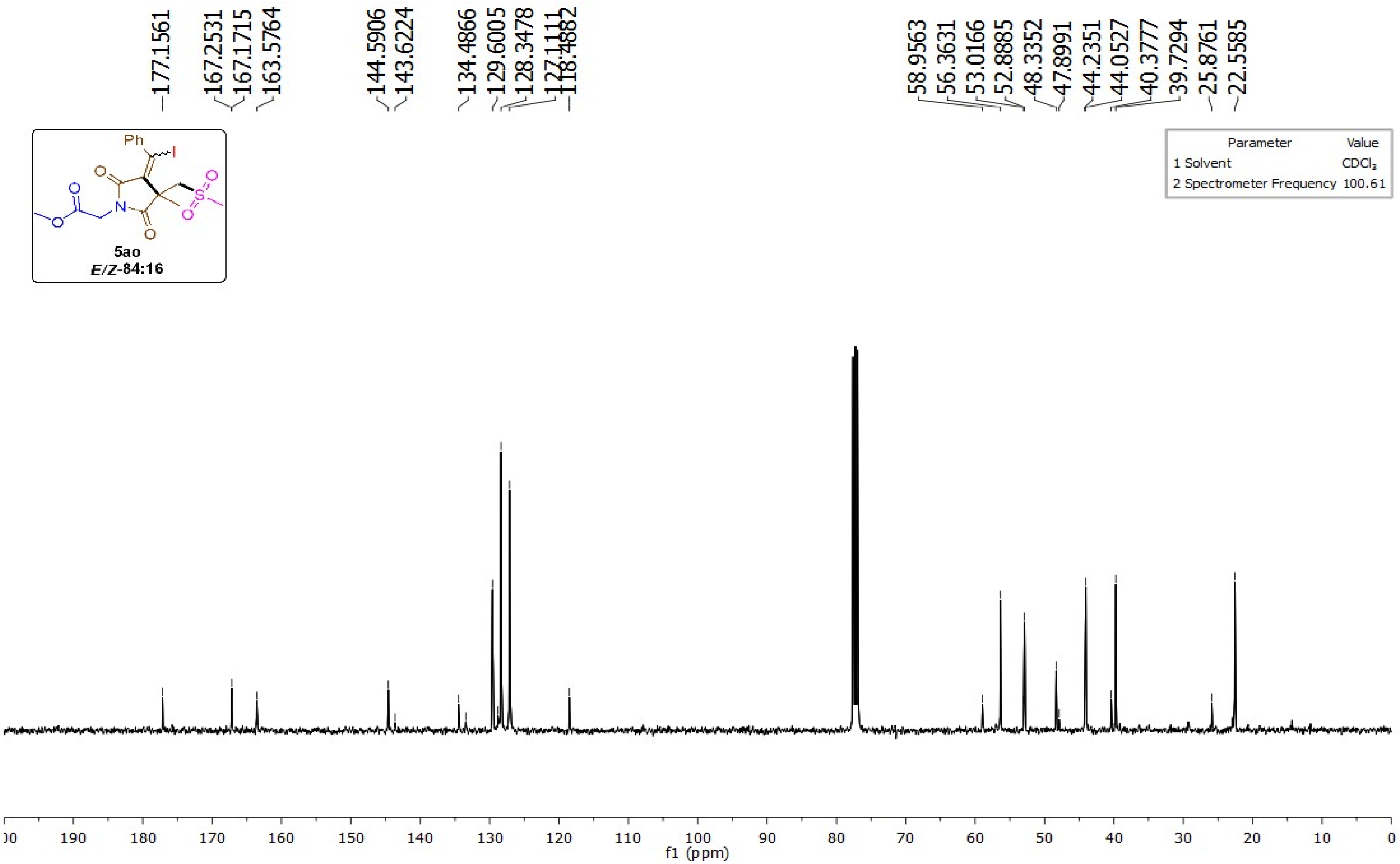

Figure S30. ^{13}C NMR spectra of Methyl 2-(4-(iodo(phenyl)methylene)-3-methyl-3-((methylsulphonyl)methyl)-2,5-dioxo pyrrolidin-1-yl)acetate (**5ao**)

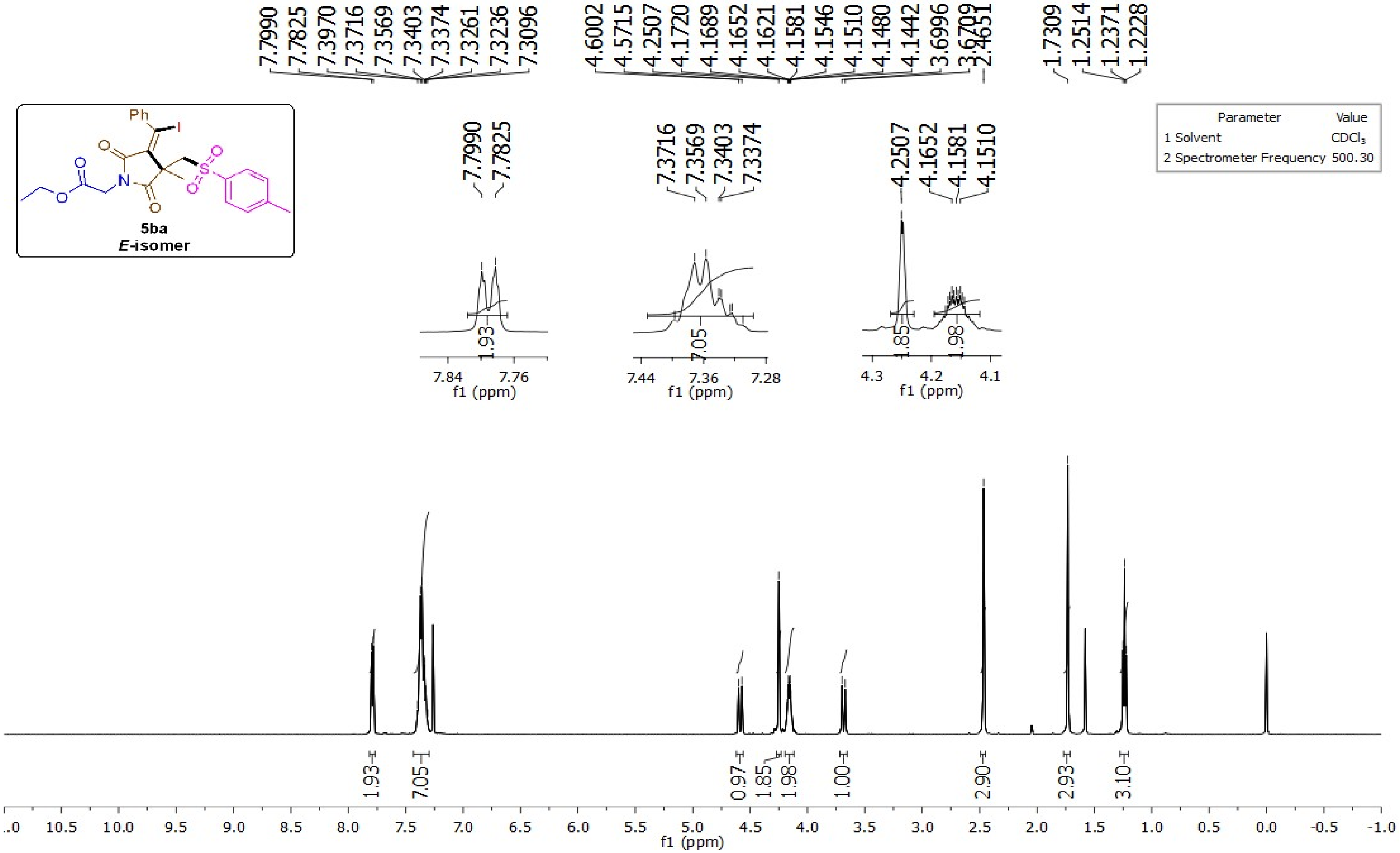

Figure S31. ^{1}H NMR spectra of Ethyl (*E*)-2-(4-(iodo(phenyl)methylene)-3-methyl-2,5-dioxo-3-(tosylmethyl)pyrrolidin-1-yl)acetate (**5ba**)

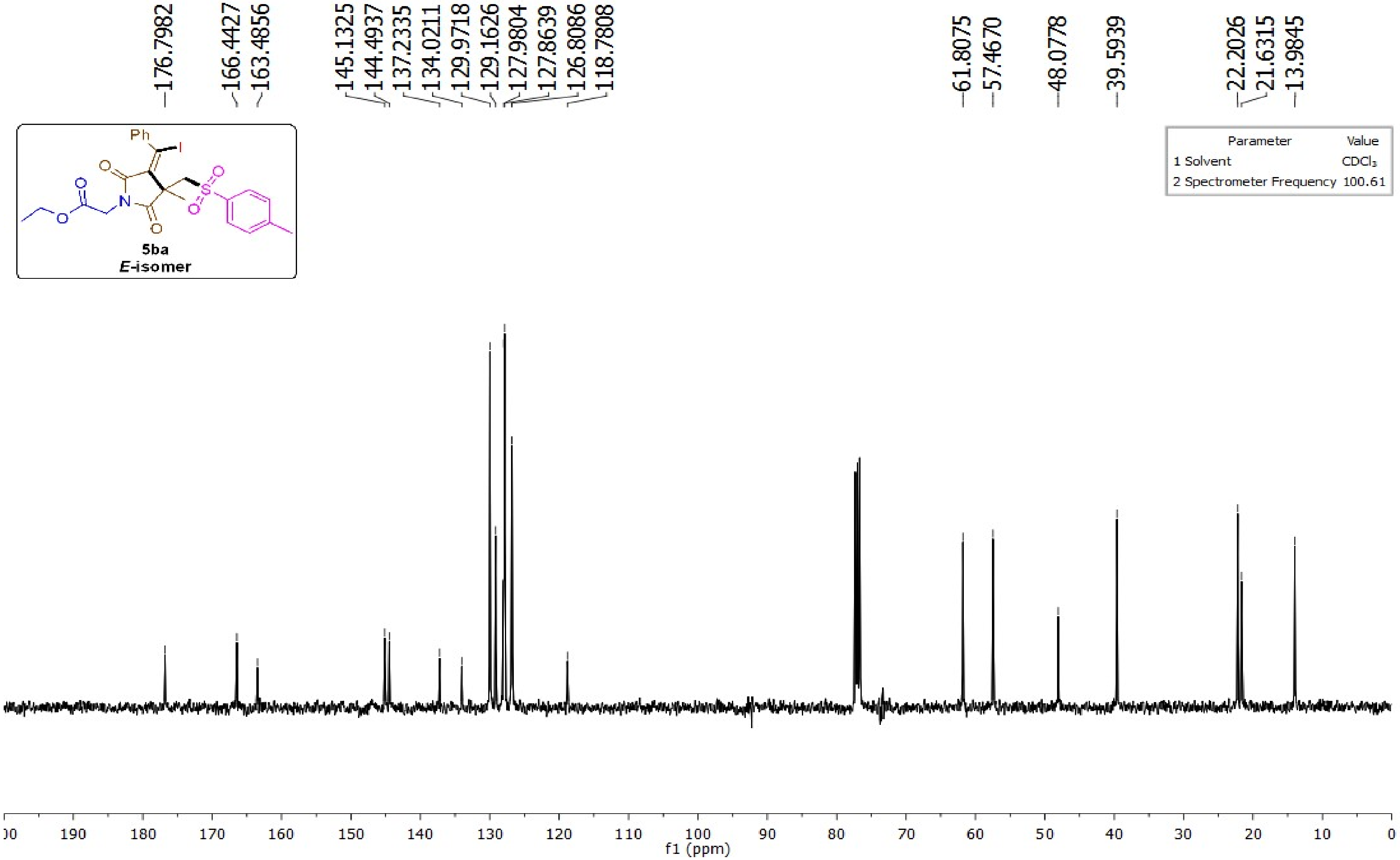

Figure S32. ^{13}C NMR spectra of Ethyl (*E*)-2-(4-(iodo(phenyl)methylene)-3-methyl-2,5-dioxo-3-(tosylmethyl)pyrrolidin-1-yl)acetate (**5ba**)

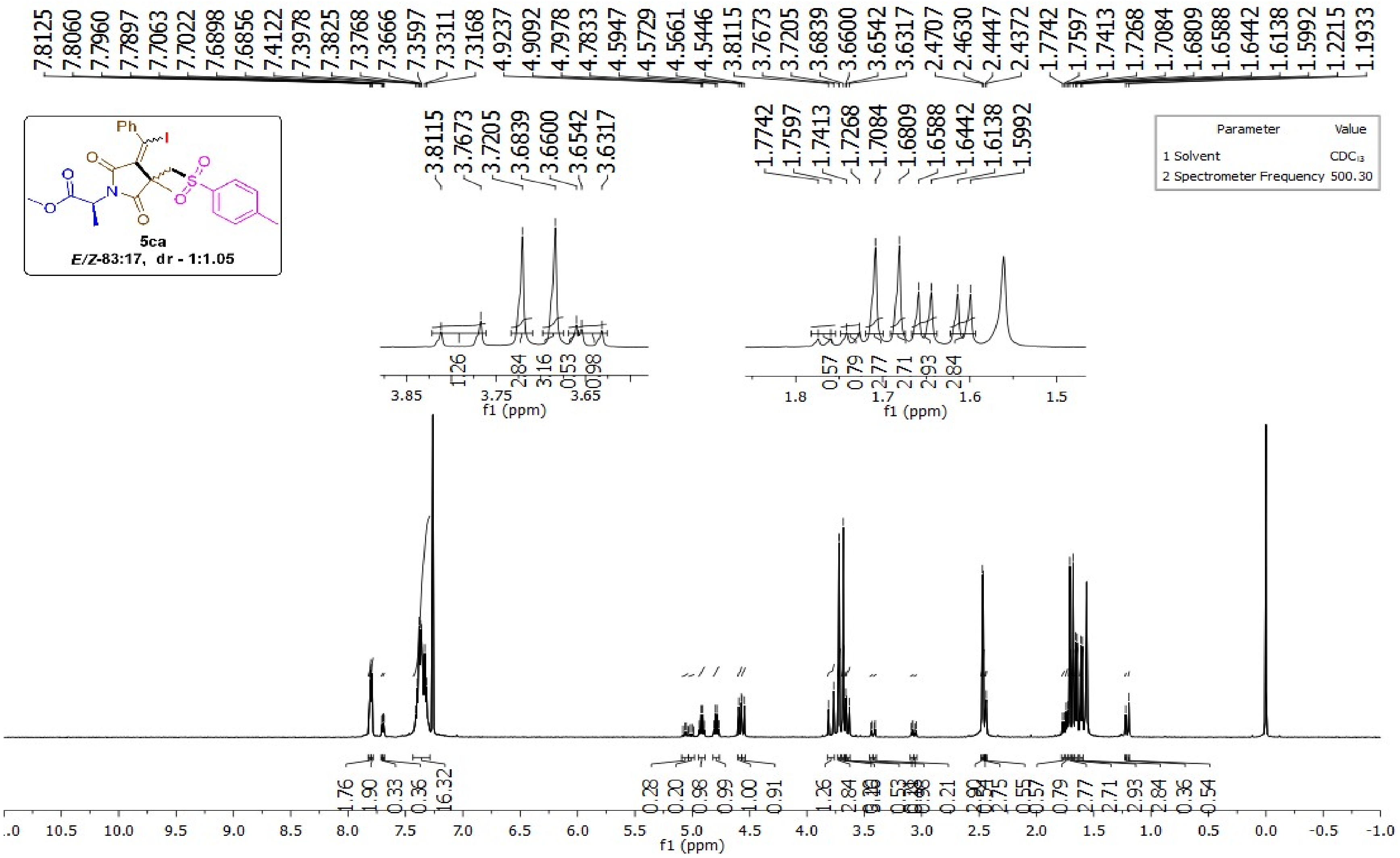

Figure S33. ^{1}H NMR spectra of (*S*)-methyl 2-(4-(iodo(phenyl)methylene)-3-methyl-2,5-dioxo-3-(tosylmethyl)pyrrolidin-1-yl)propanoate (**5ca**)

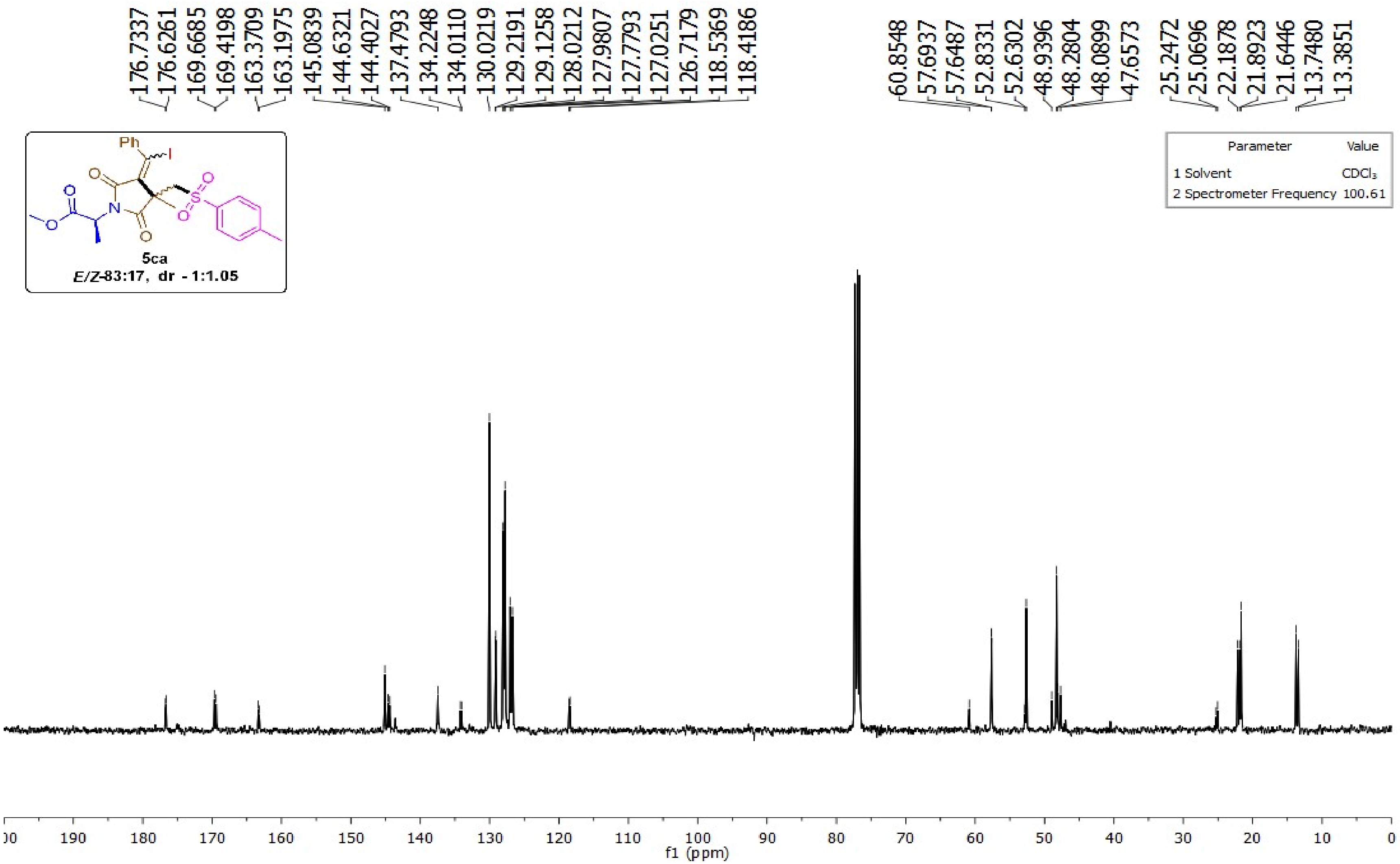

Figure S34. ^{13}C NMR spectra of (*S*)-methyl 2-(4-(iodo(phenyl)methylene)-3-methyl-2,5-dioxo-3-(tosylmethyl)pyrrolidin-1-yl)propanoate (**5ca)**

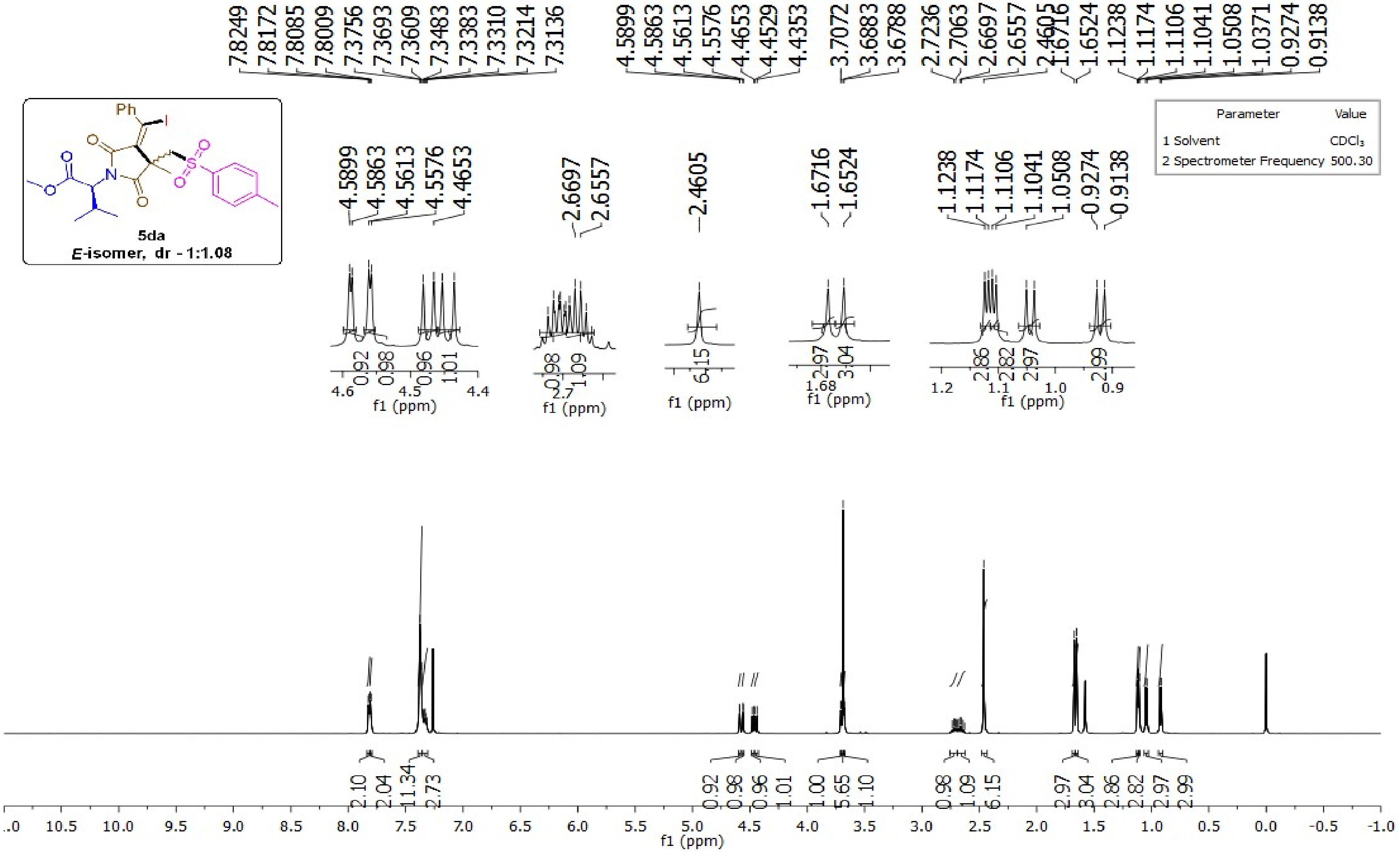

Figure S35. ^{1}H NMR spectra of (*S,E*)-methyl 2-(4-(iodo(phenyl)methylene)-3-methyl-2,5-dioxo-3-(tosylmethyl)pyrrolidin-1-yl)-3-methyl butanoate **(5da)**

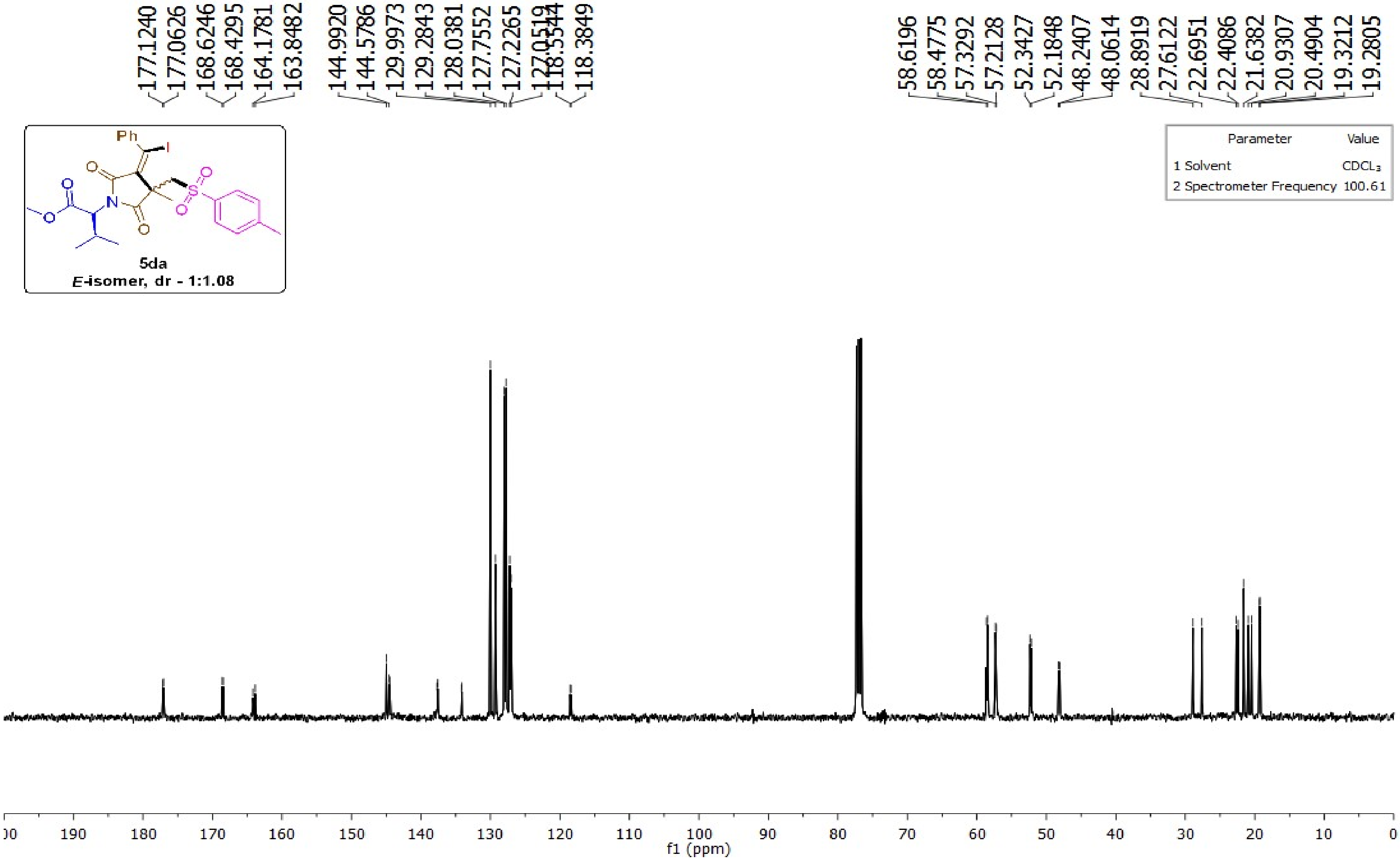

Figure S36. ^{13}C NMR spectra of (*S,E*)-methyl 2-(4-(iodo(phenyl)methylene)-3-methyl-2,5-dioxo-3-(tosylmethyl)pyrrolidin-1-yl)-3-methyl butanoate **(5da)**

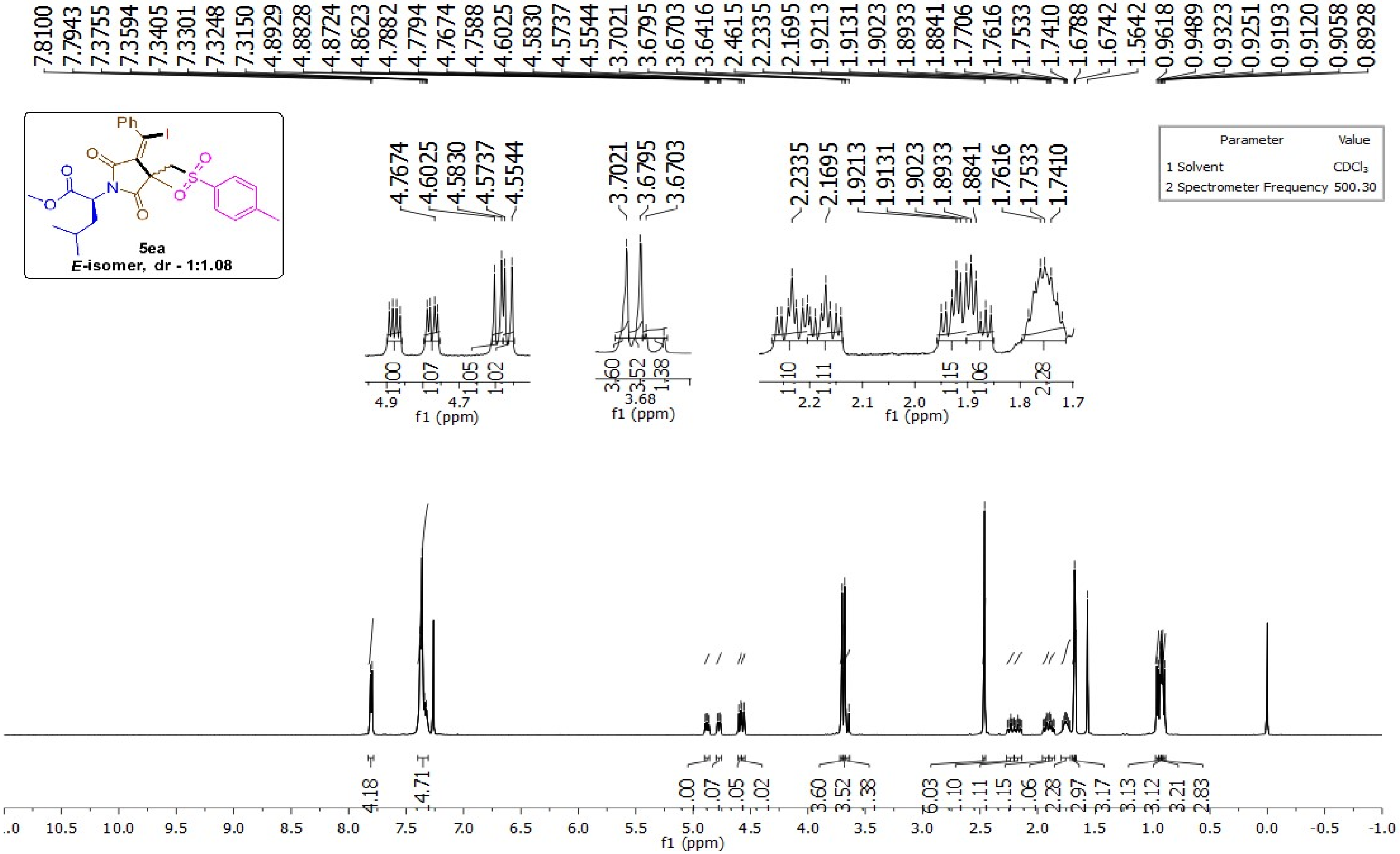

Figure S37. ^{1}H NMR spectra of (*S,E*)-methyl 2-(4-(iodo(phenyl)methylene)-3-methyl-2,5-dioxo-3-(tosylmethyl)pyrrolidin-1-yl)-4-methylpentanoate **(5ea)**

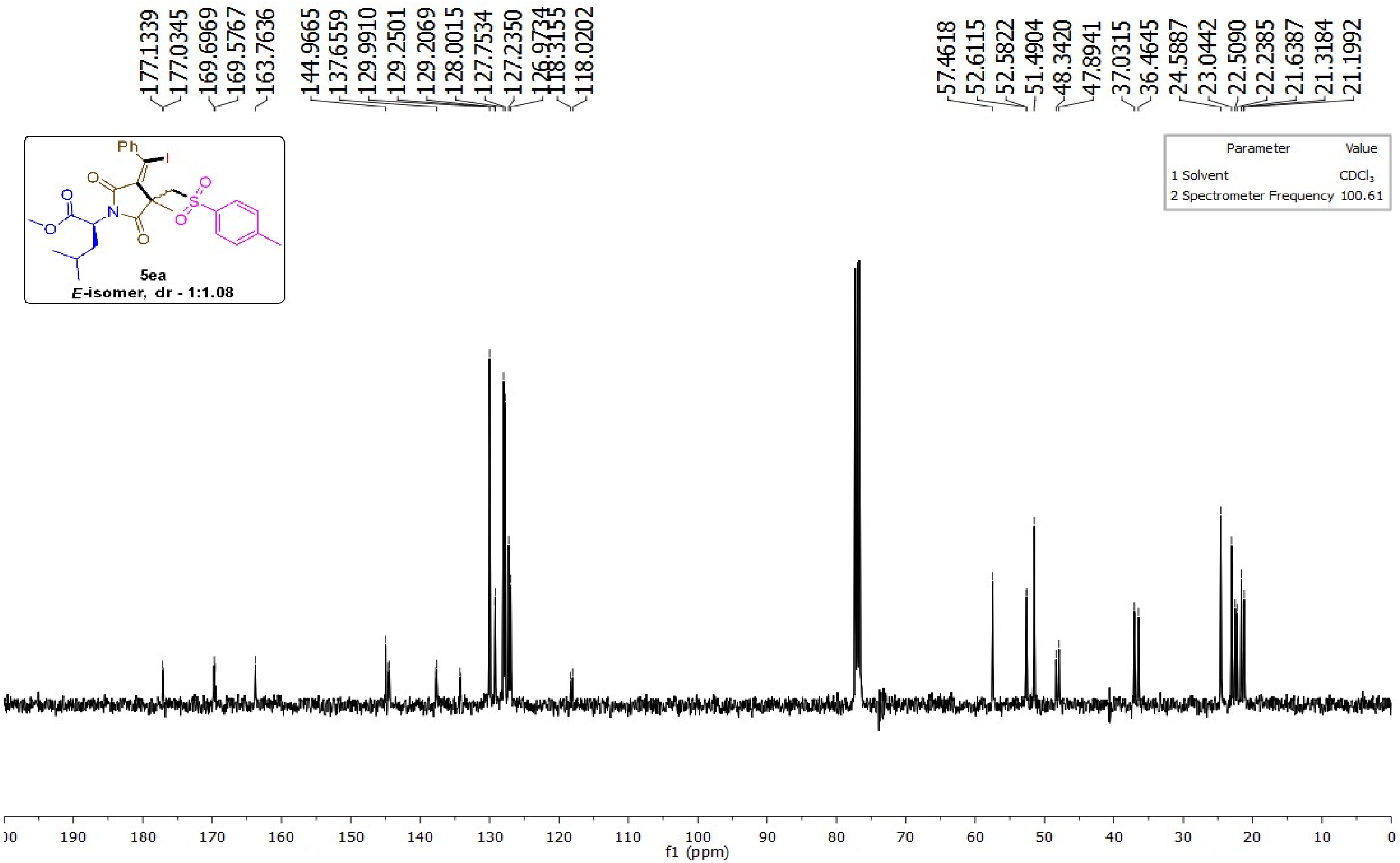

Figure S38. ^{13}C NMR spectra of (*S,E*)-methyl 2-(4-(iodo(phenyl)methylene)-3-methyl-2,5-dioxo-3-(tosylmethyl)pyrrolidin-1-yl)-4-methylpentanoate (**5ea**)

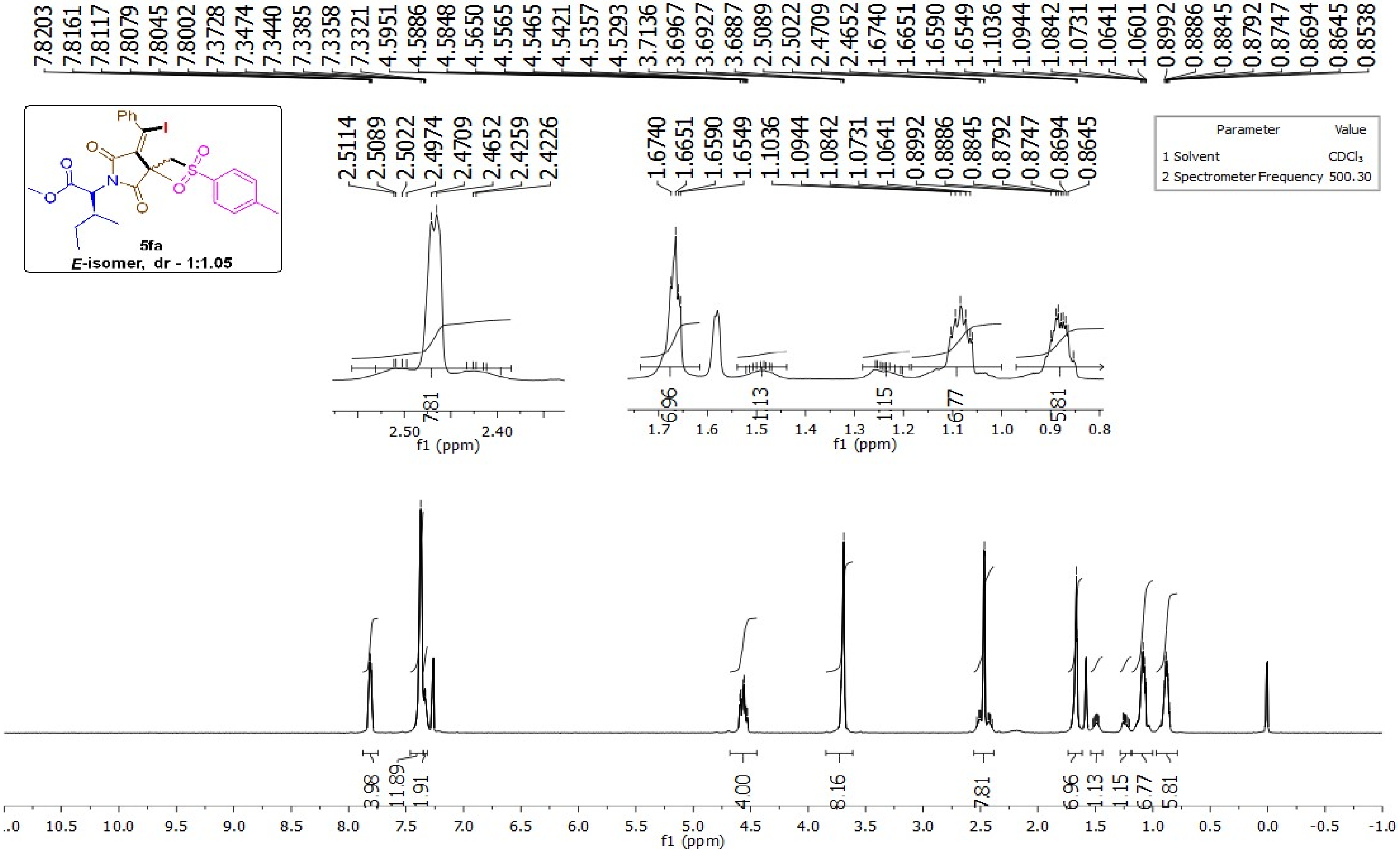

Figure S39. ^{1}H NMR spectra of (2*S*,3*R*)-methyl 2-((*S*,*E*)-4-(iodo(phenyl)methylene)-3-methyl-2,5-dioxo-3-(tosylmethyl)pyrrolidin-1-yl)-3-methylpentanoate **(5fa)**

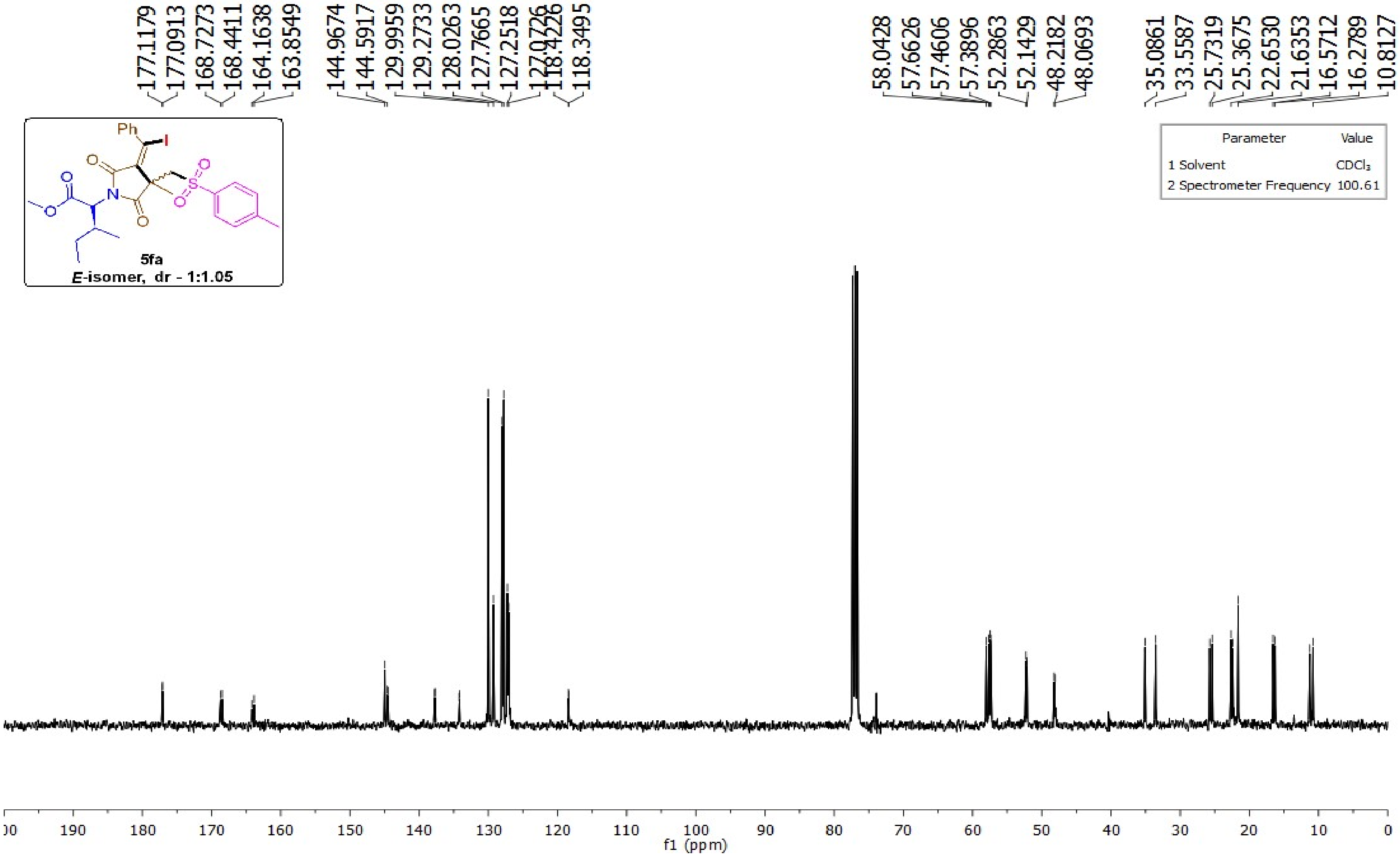

Figure S40. ^{13}C NMR spectra of (2*S*,3*R*)-methyl 2-((*S*,*E*)-4-(iodo(phenyl)methylene)-3-methyl-2,5-dioxo-3-(tosylmethyl)pyrrolidin-1-yl)-3-methylpentanoate **(5fa)**

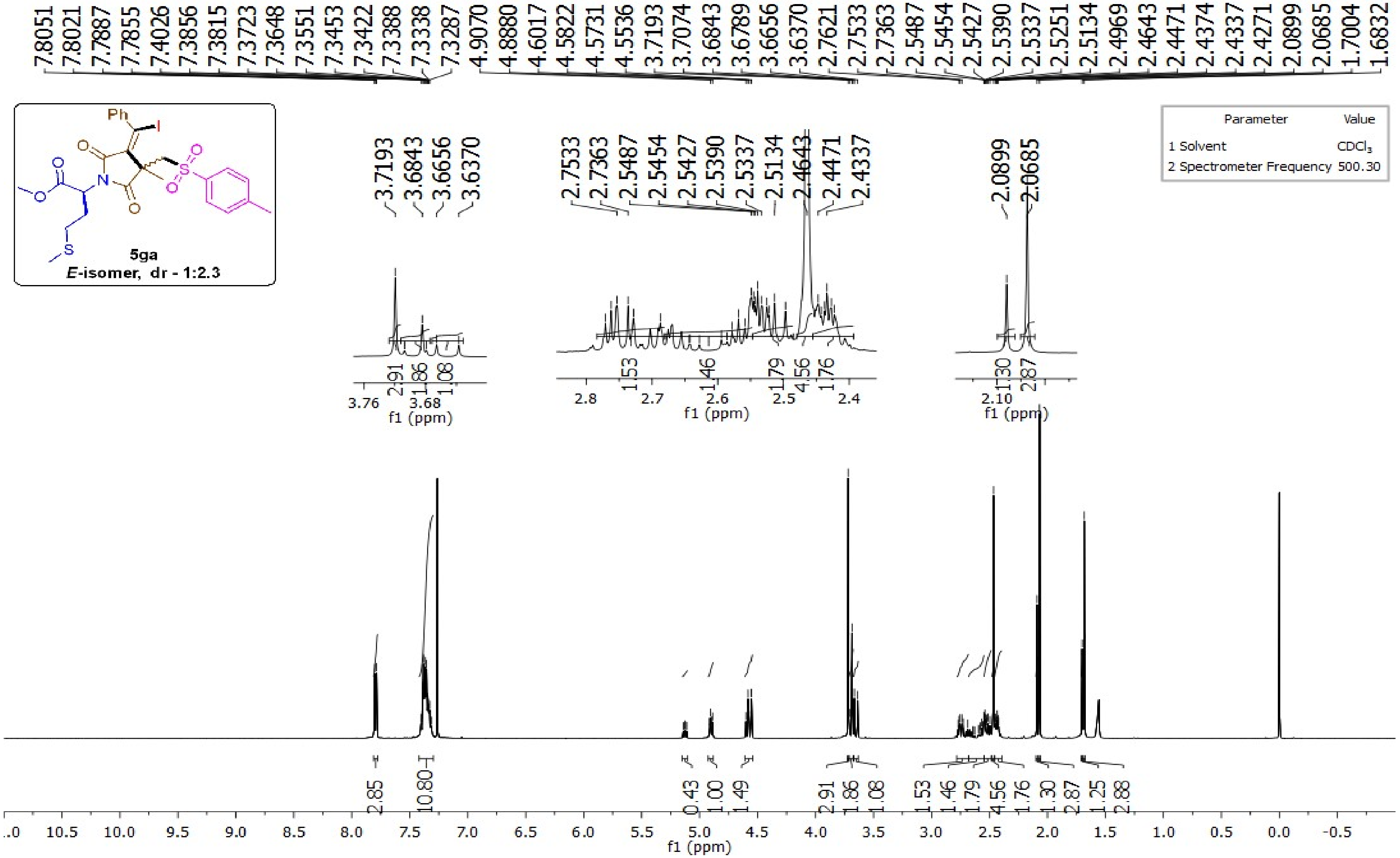

Figure S41. ^{1}H NMR spectra of (*S,E*)-methyl 2-(4-(iodo(phenyl)methylene)-3-methyl-2,5-dioxo-3-(tosylmethyl)pyrrolidin-1-yl)-4-(methylthio)butanoate **(5ga)**

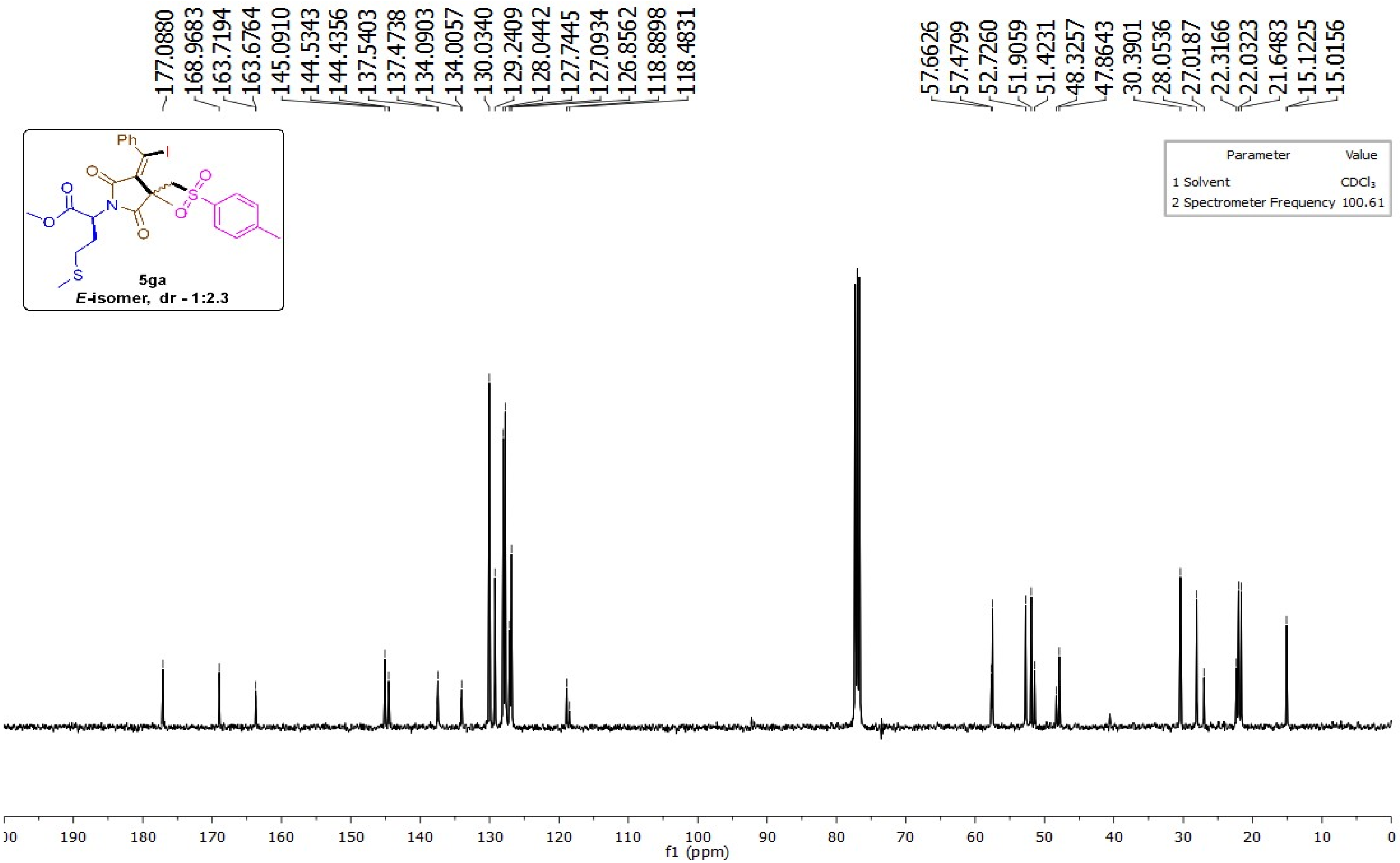

Figure S42. ^{13}C NMR spectra of (*S,E*)-methyl 2-(4-(iodo(phenyl)methylene)-3-methyl-2,5-dioxo-3-(tosylmethyl)pyrrolidin-1-yl)-4-(methylthio)butanoate **(5ga)**

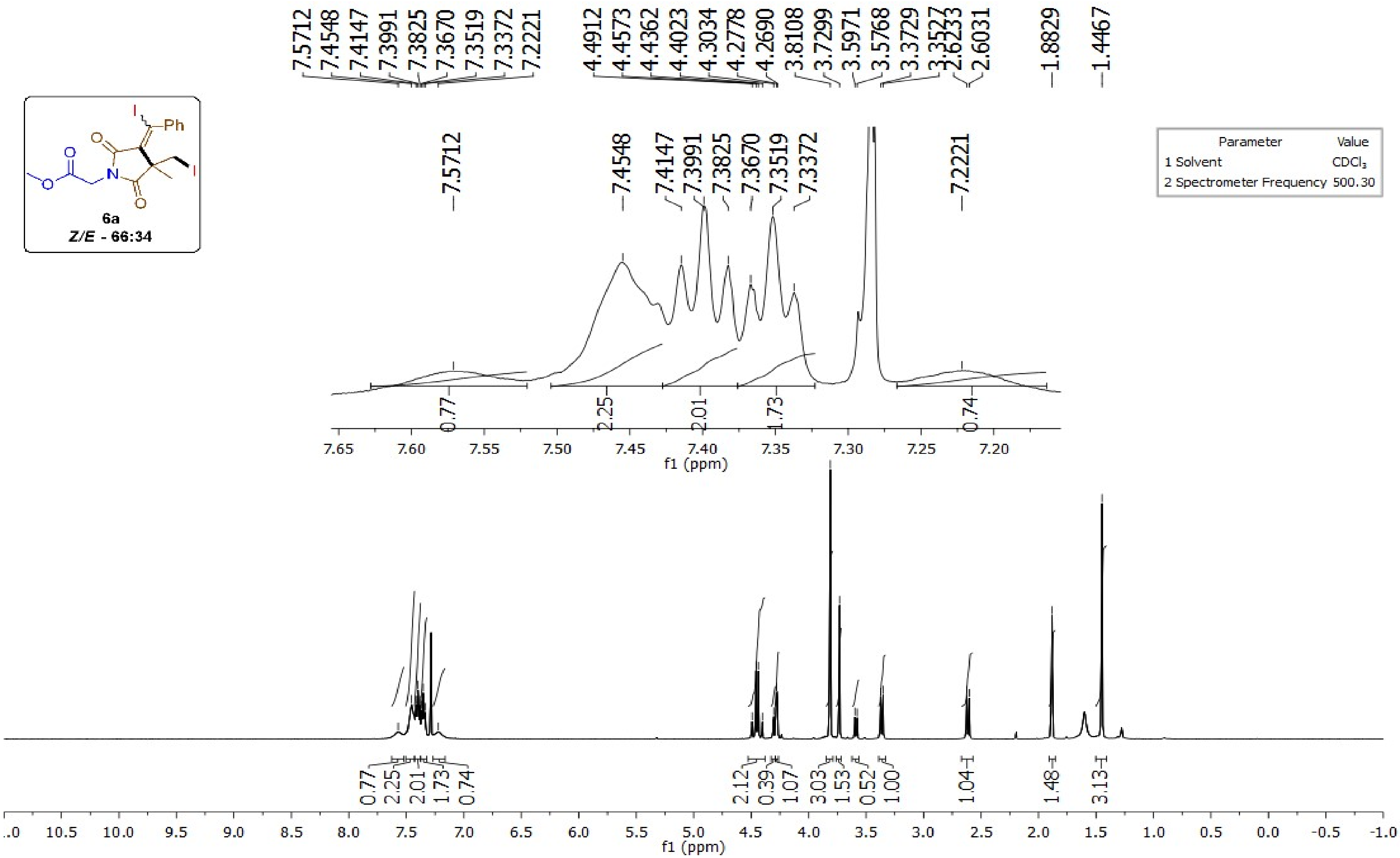

Figure S43. ^{1}H NMR spectra of Methyl 2-(4-(iodo(phenyl)methylene)-3-(iodomethyl)-3-methyl-2,5-dioxopyrrolidin-1-yl)acetate (**6a**)

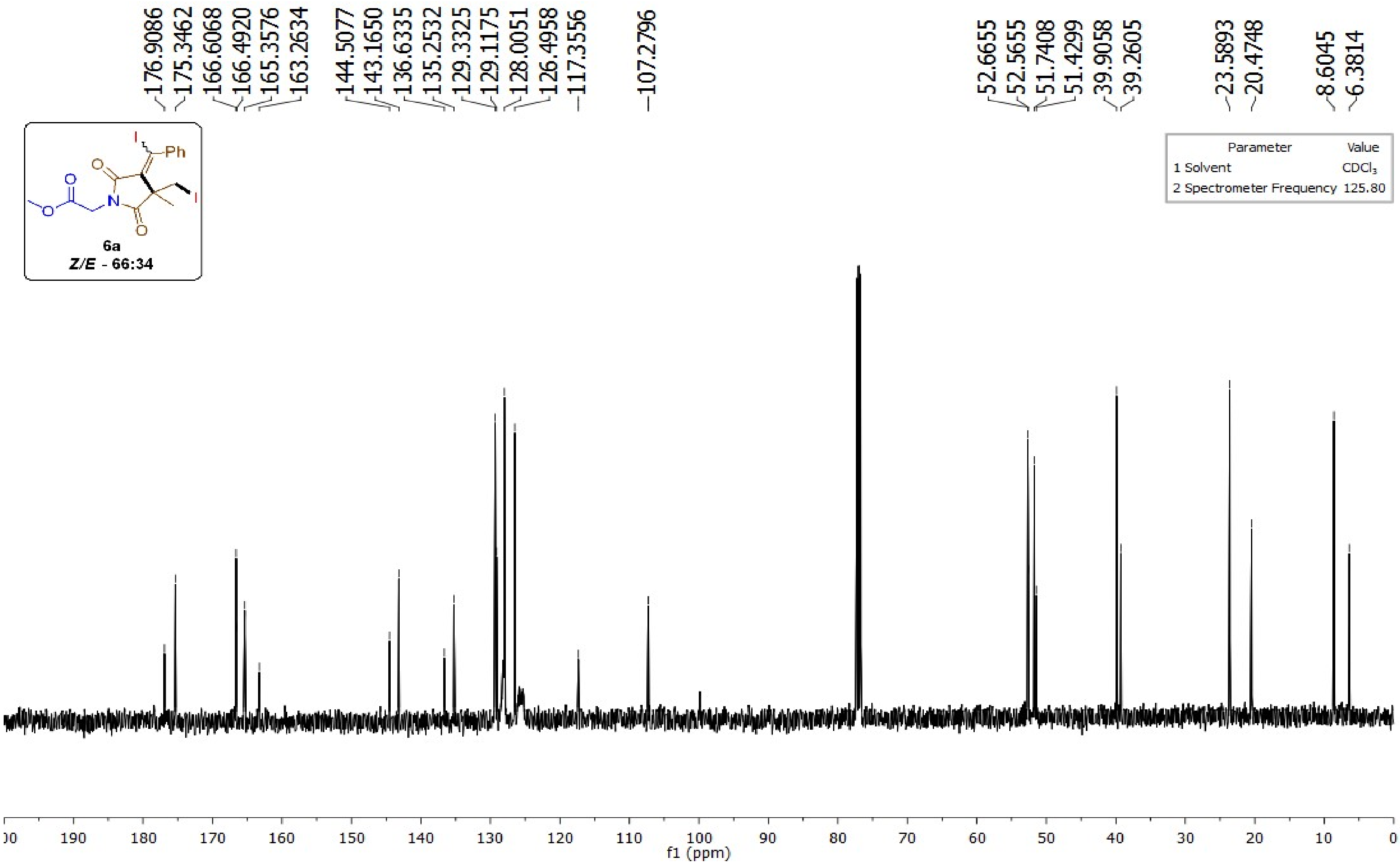

Figure S44. ^{13}C NMR spectra of Methyl 2-(4-(iodo(phenyl)methylene)-3-(iodomethyl)-3-methyl-2,5-dioxopyrrolidin-1-yl)acetate (**6a**)

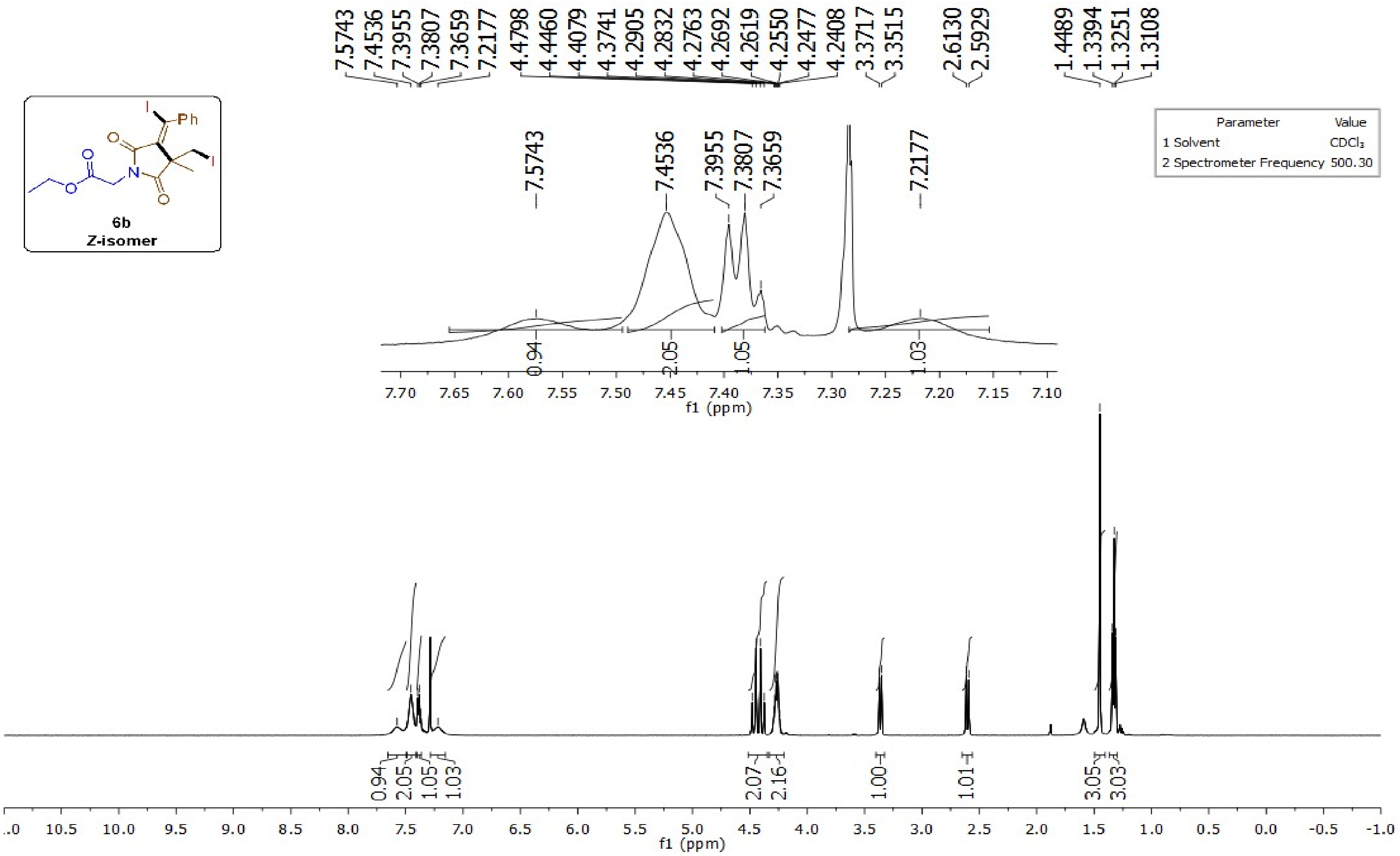

Figure S45. ^{1}H NMR spectra of Ethyl (*Z*)-2-(4-(iodo(phenyl)methylene)-3-(iodomethyl)-3-methyl-2,5-dioxopyrrolidin-1-yl)acetate (**6b**)

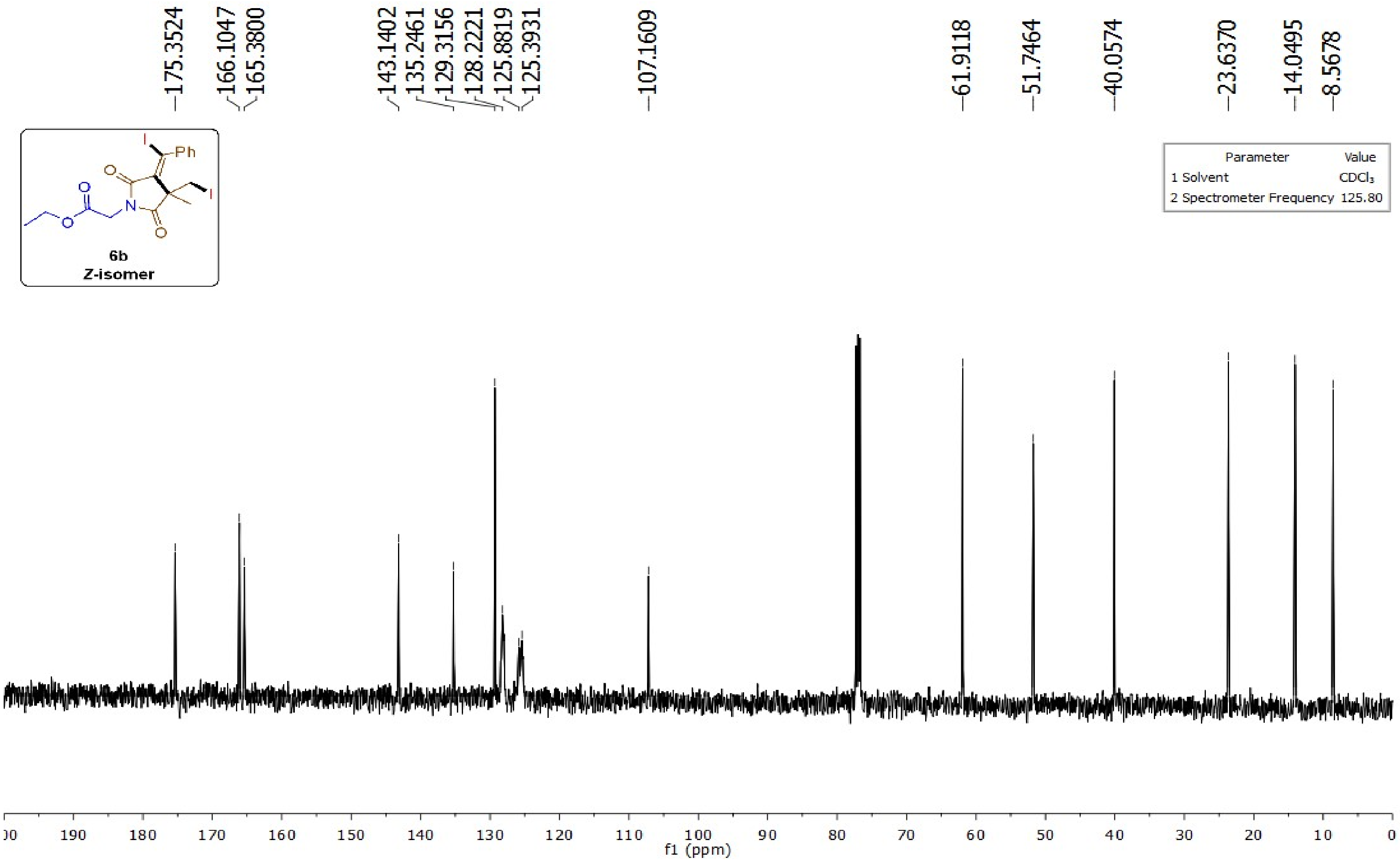

Figure S46. ^{13}C NMR spectra of Ethyl (*Z*)-2-(4-(iodo(phenyl)methylene)-3-(iodomethyl)-3-methyl-2,5-dioxopyrrolidin-1-yl)acetate (**6b**)

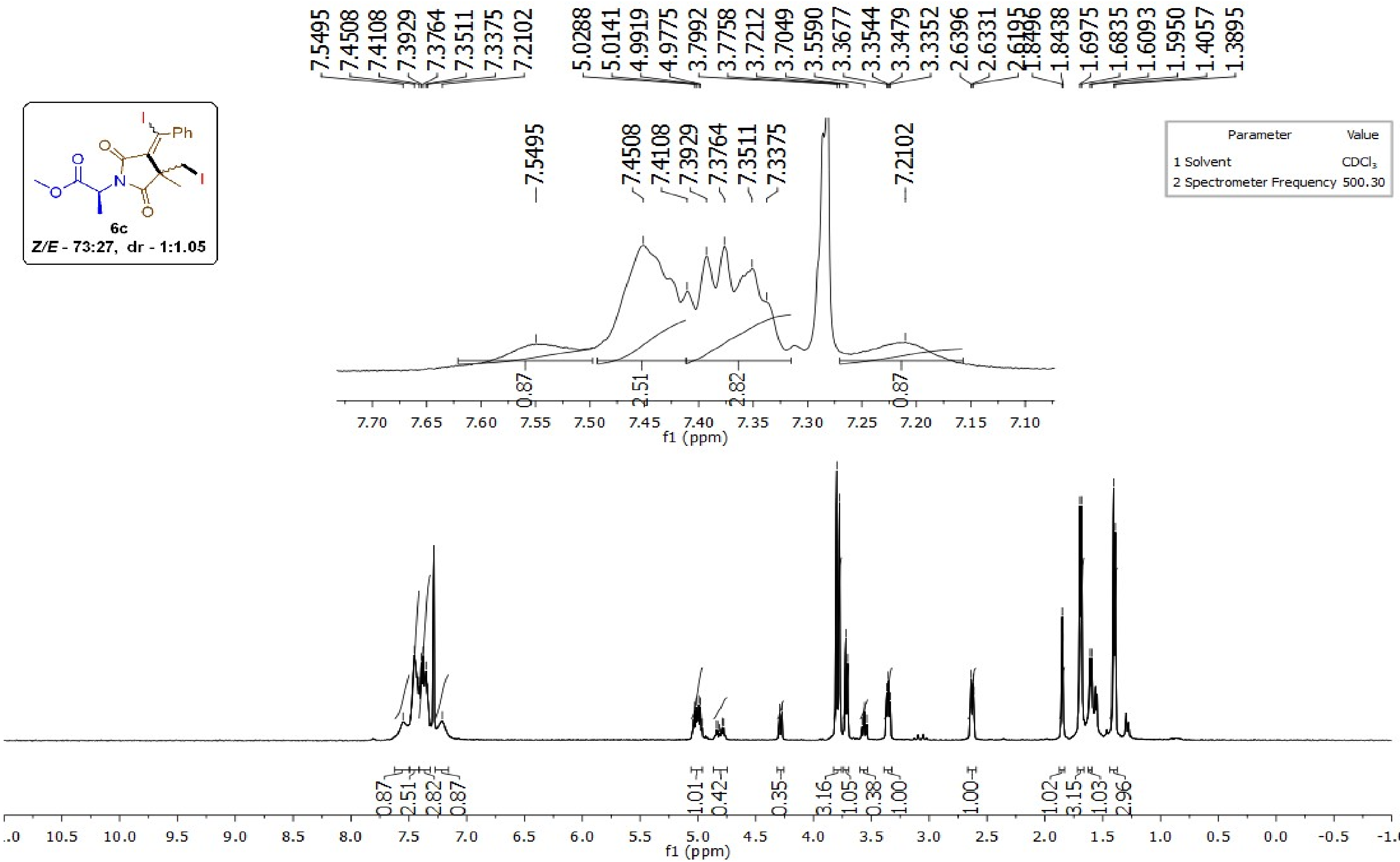

Figure S47. ^{1}H NMR spectra of (*S*)-methyl 2-(4-(iodo(phenyl)methylene)-3-(iodo methyl)-3-methyl-2,5-dioxopyrrolidin-1-yl)propanoate **(6c)**

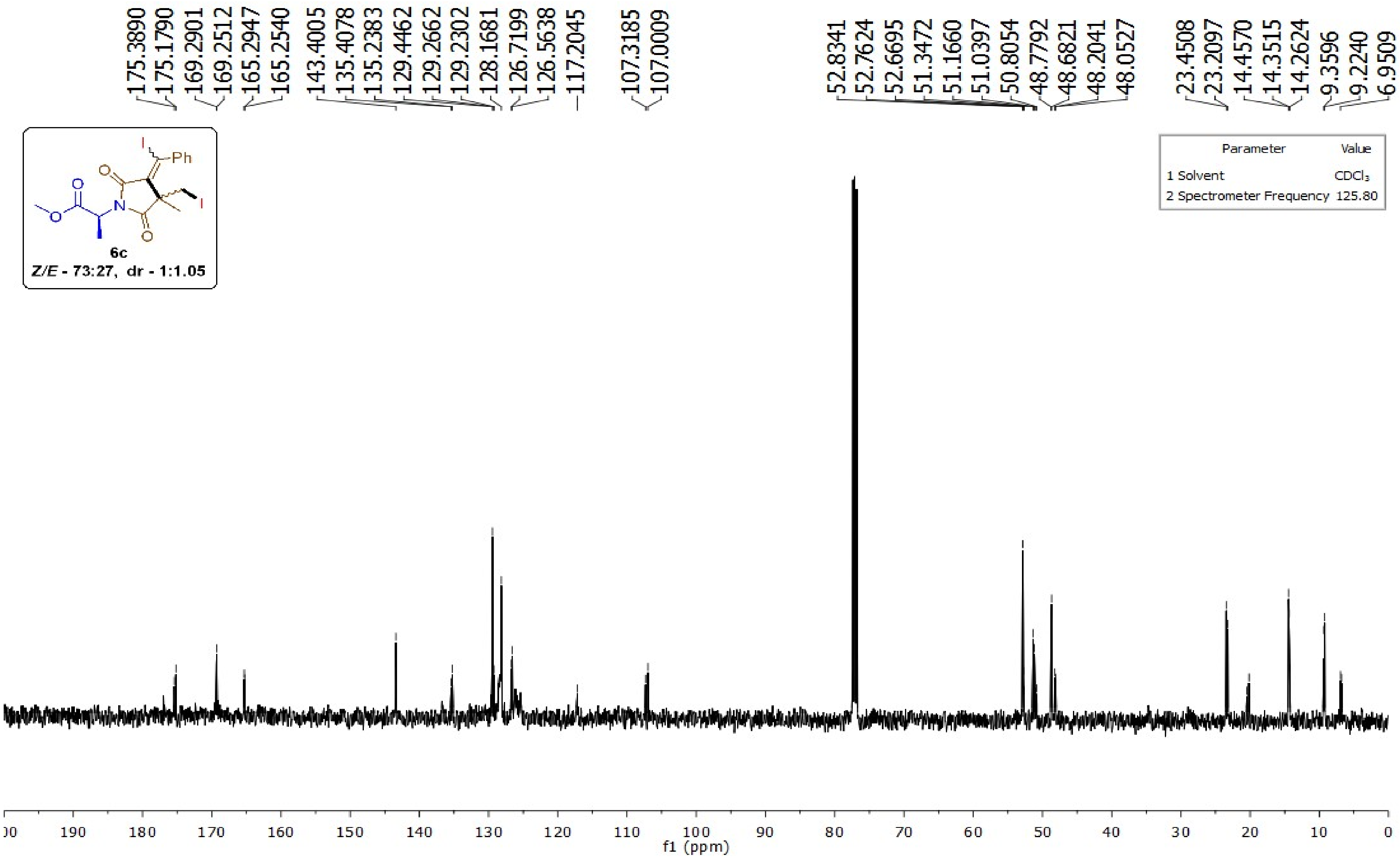

Figure S48. ^{13}C NMR spectra of (*S*)-methyl 2-(4-(iodo(phenyl)methylene)-3-(iodo methyl)-3-methyl-2,5-dioxopyrrolidin-1-yl)propanoate **(6c)**

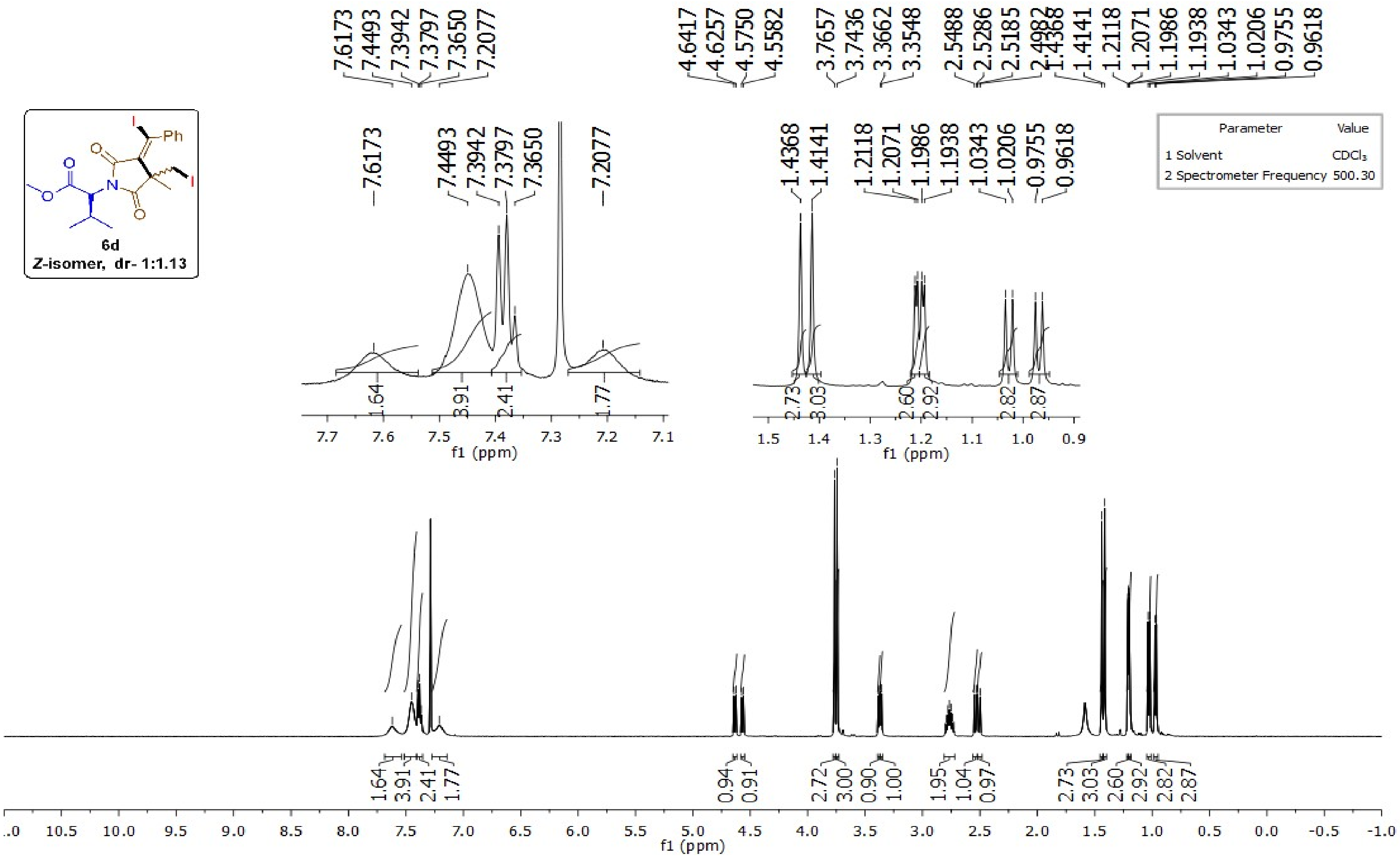

Figure S49. ^{1}H NMR spectra of (*S,Z*)-methyl 2-(4-(iodo(phenyl)methylene)-3-(iodomethyl)-3-methyl-2,5-dioxopyrrolidin-1-yl)-3-methylbutanoate **(6d)**

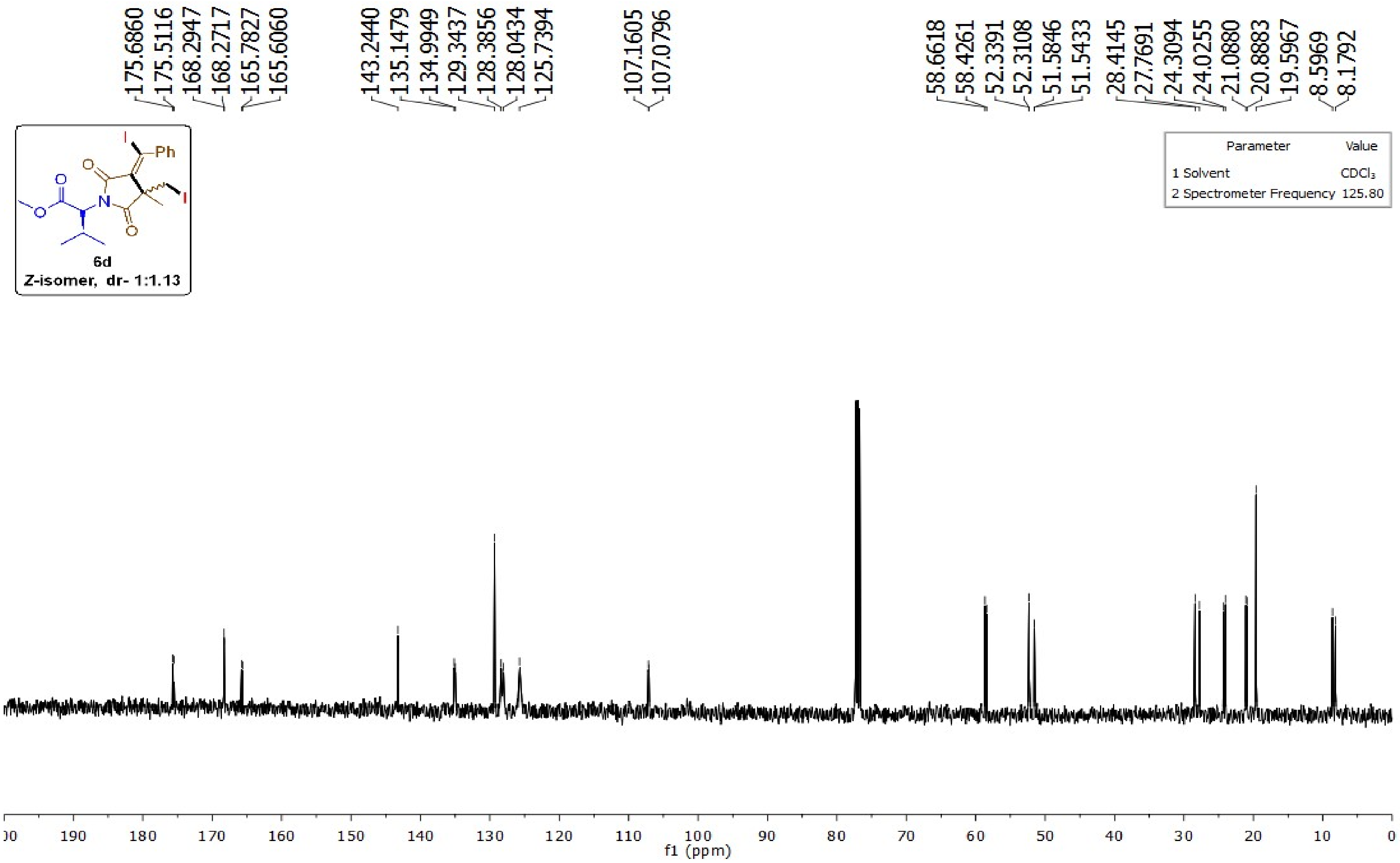

Figure 50. ^{13}C NMR spectra of (*S*,*Z*)-methyl 2-(4-(iodo(phenyl)methylene)-3-(iodomethyl)-3-methyl-2,5-dioxopyrrolidin-1-yl)-3-methylbutanoate **(6d)**

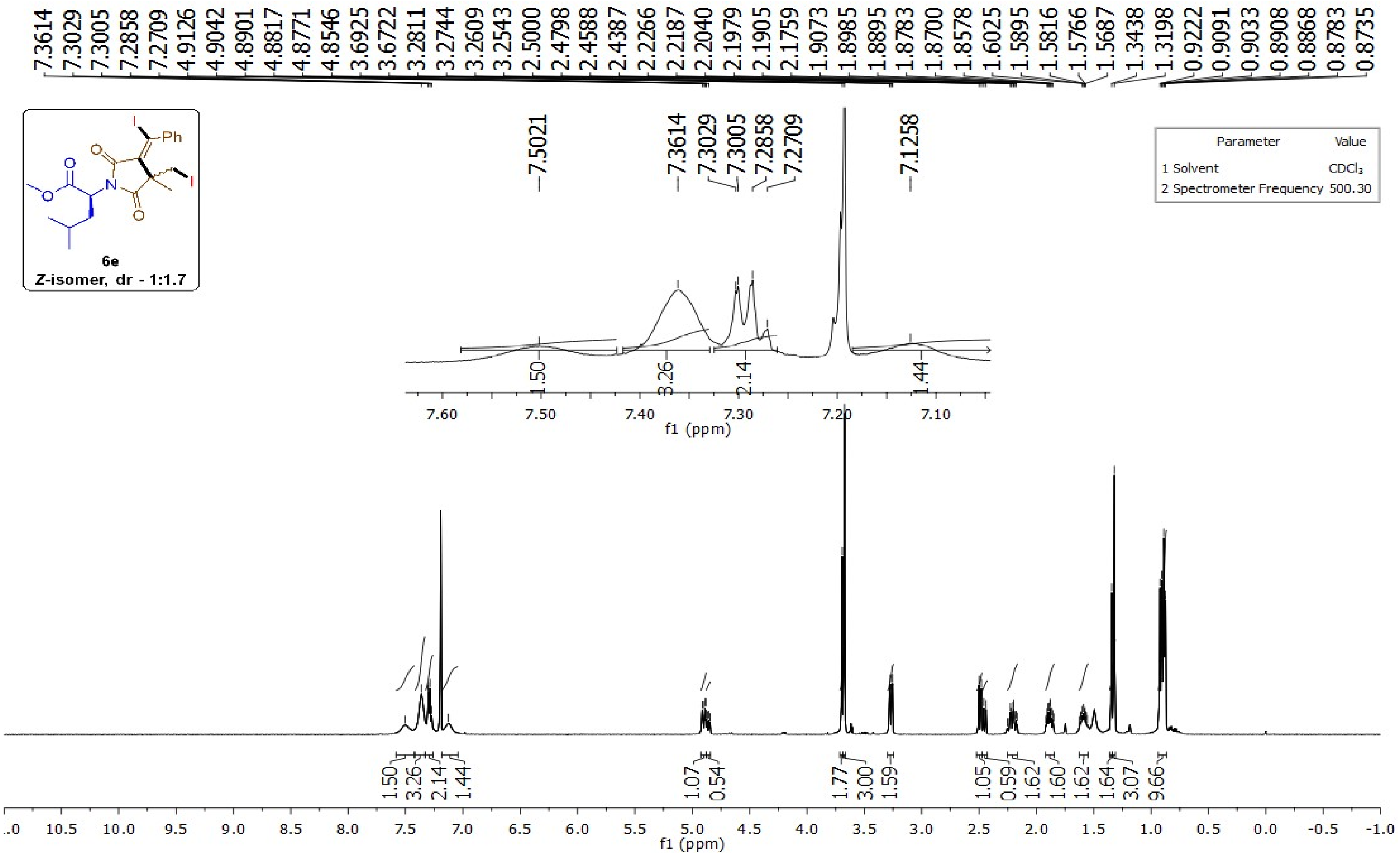

Figure S51. ^{1}H NMR spectra of (*S,Z*)-methyl 2-(4-(iodo(phenyl)methylene)-3-(iodomethyl)-3-methyl-2,5-dioxopyrrolidin-1-yl)-4-methylpentanoate **(6e)**

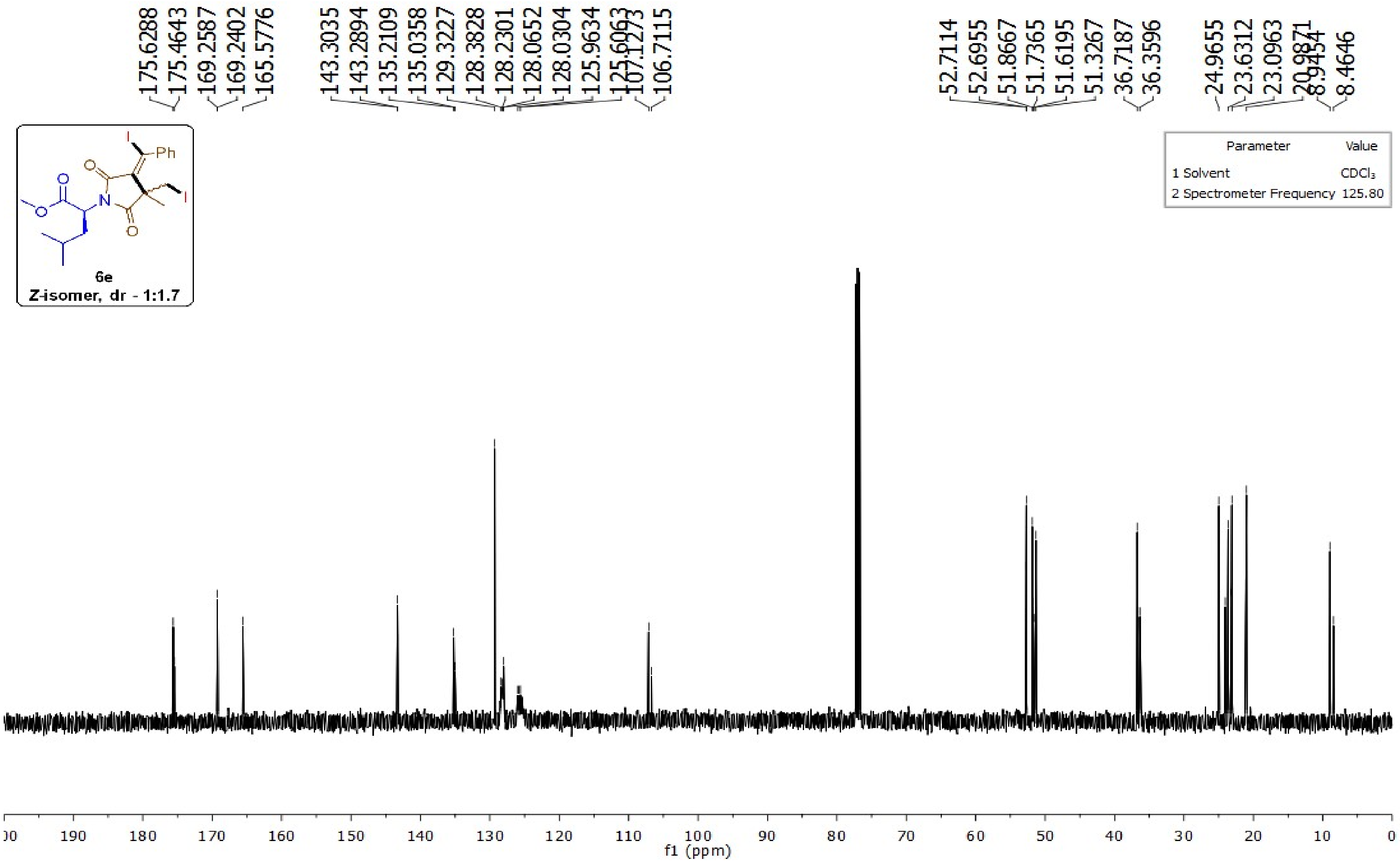

Figure S52. ^{13}C NMR spectra of (*S,Z*)-methyl 2-(4-(iodo(phenyl)methylene)-3-(iodomethyl)-3-methyl-2,5-dioxopyrrolidin-1-yl)-4-methylpentanoate **(6e)**

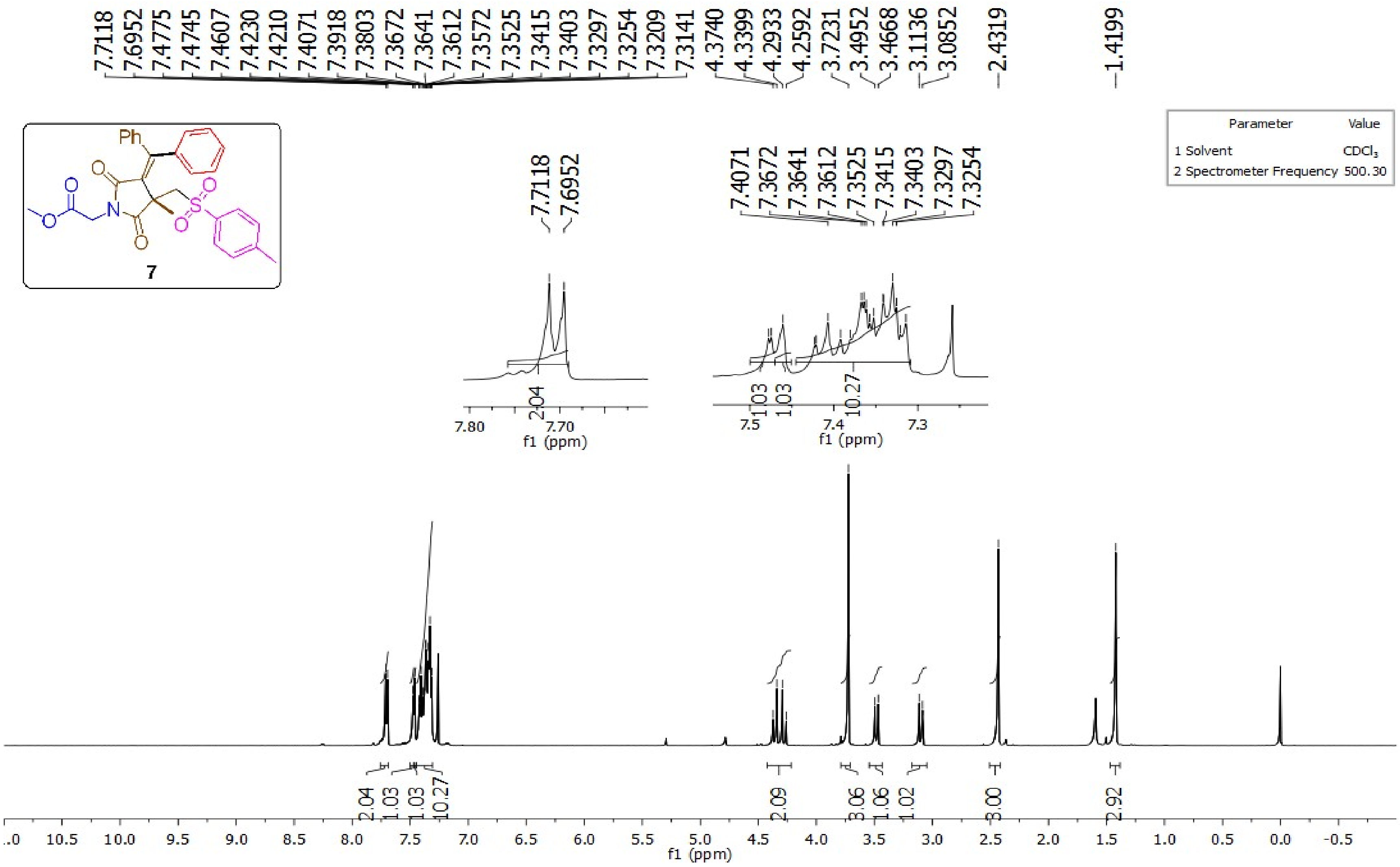

Figure S53. ^{1}H NMR spectra of Methyl 2-(4-(diphenylmethylene)-3-methyl-2,5-dioxo-3-(tosylmethyl) pyrrolidin-1-yl)acetate (**7**)

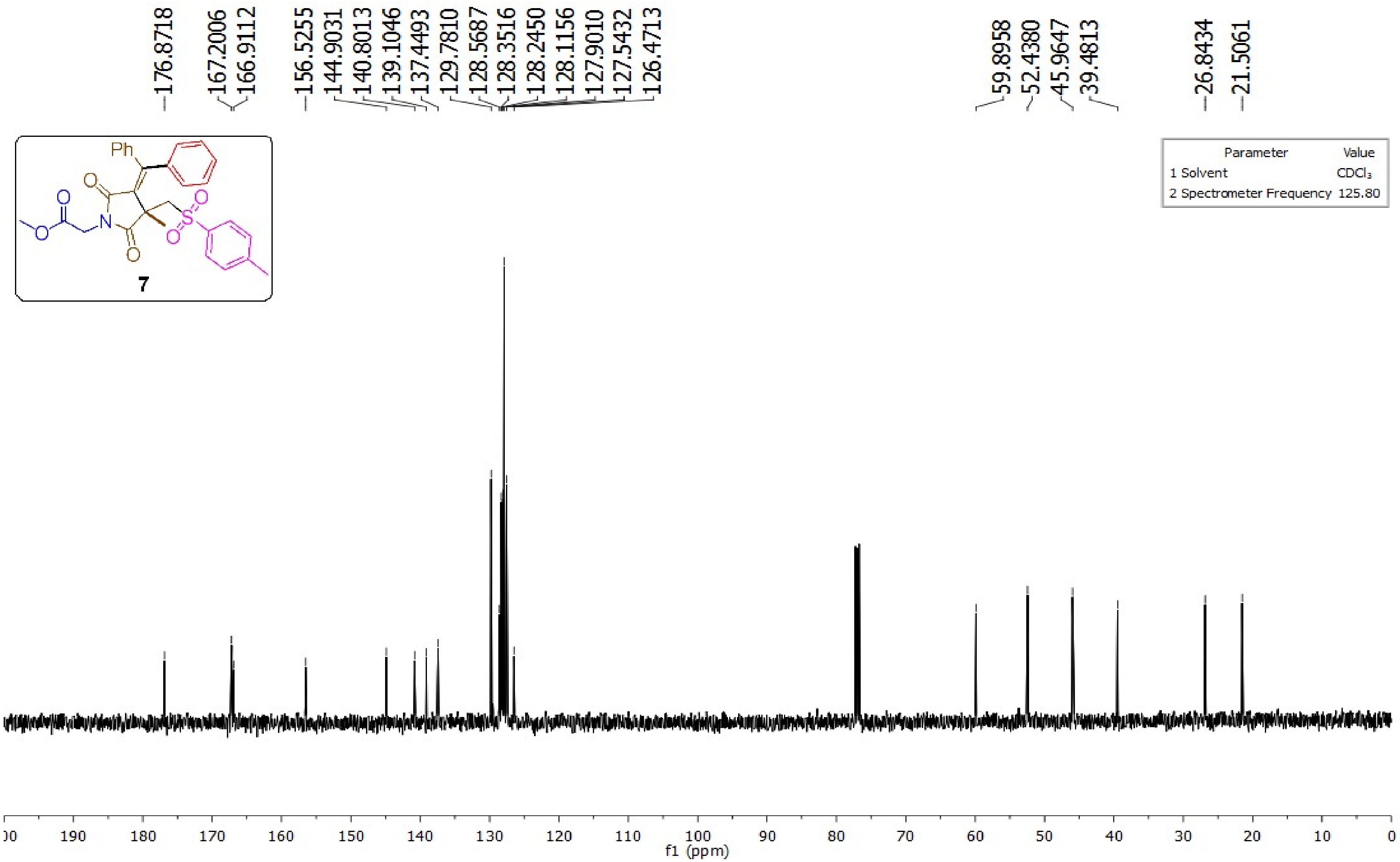

Figure S54. ^{13}C NMR spectra of Methyl 2-(4-(diphenylmethylene)-3-methyl-2,5-dioxo-3-(tosylmethyl) pyrrolidin-1-yl)acetate (**7**)

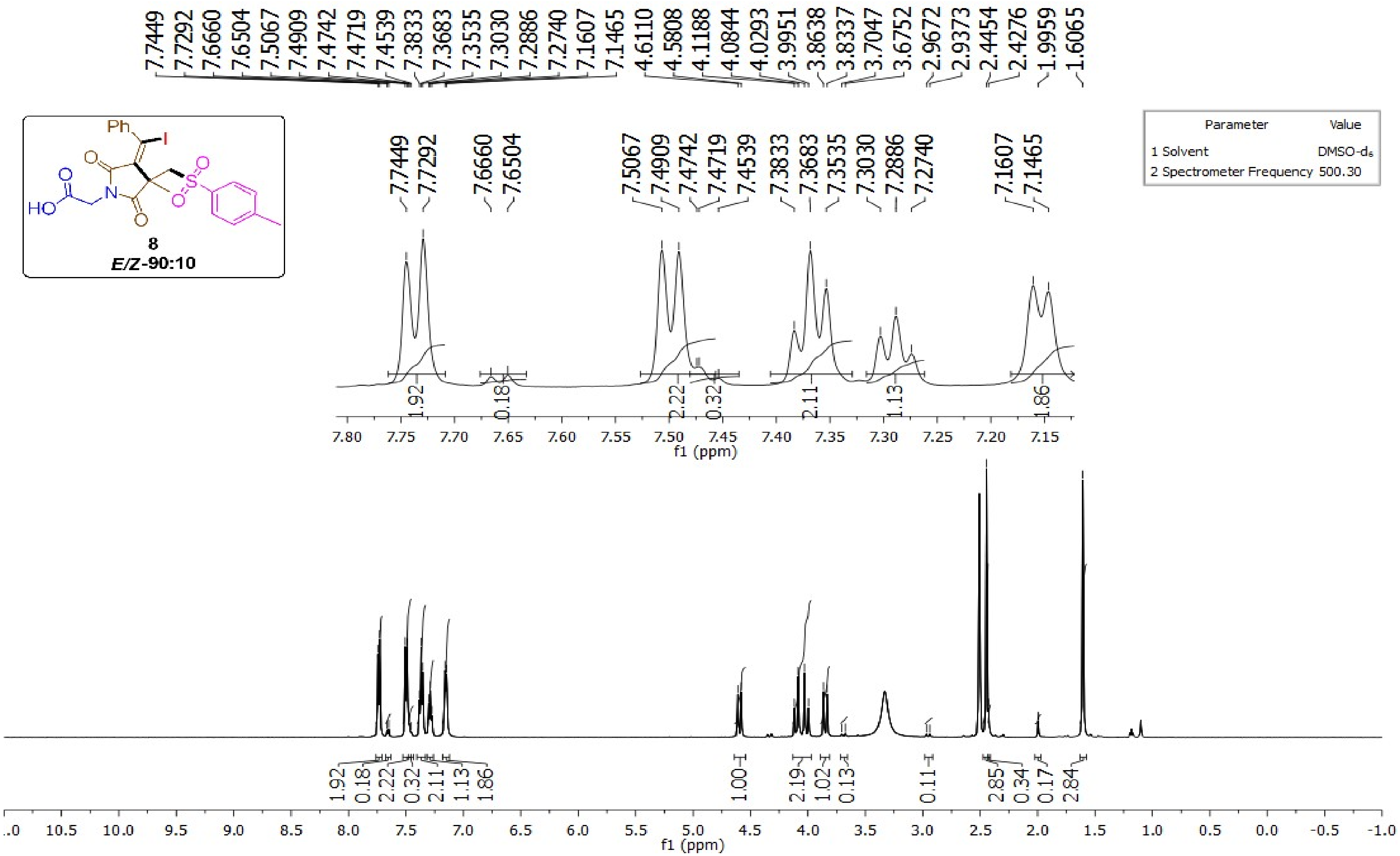

Figure S55. ^{1}H NMR spectra of 2-(4-(iodo(phenyl)methylene)-3-methyl-2,5-dioxo-3-(tosylmethyl) pyrrolidin-1-yl)acetic acid (**8**)

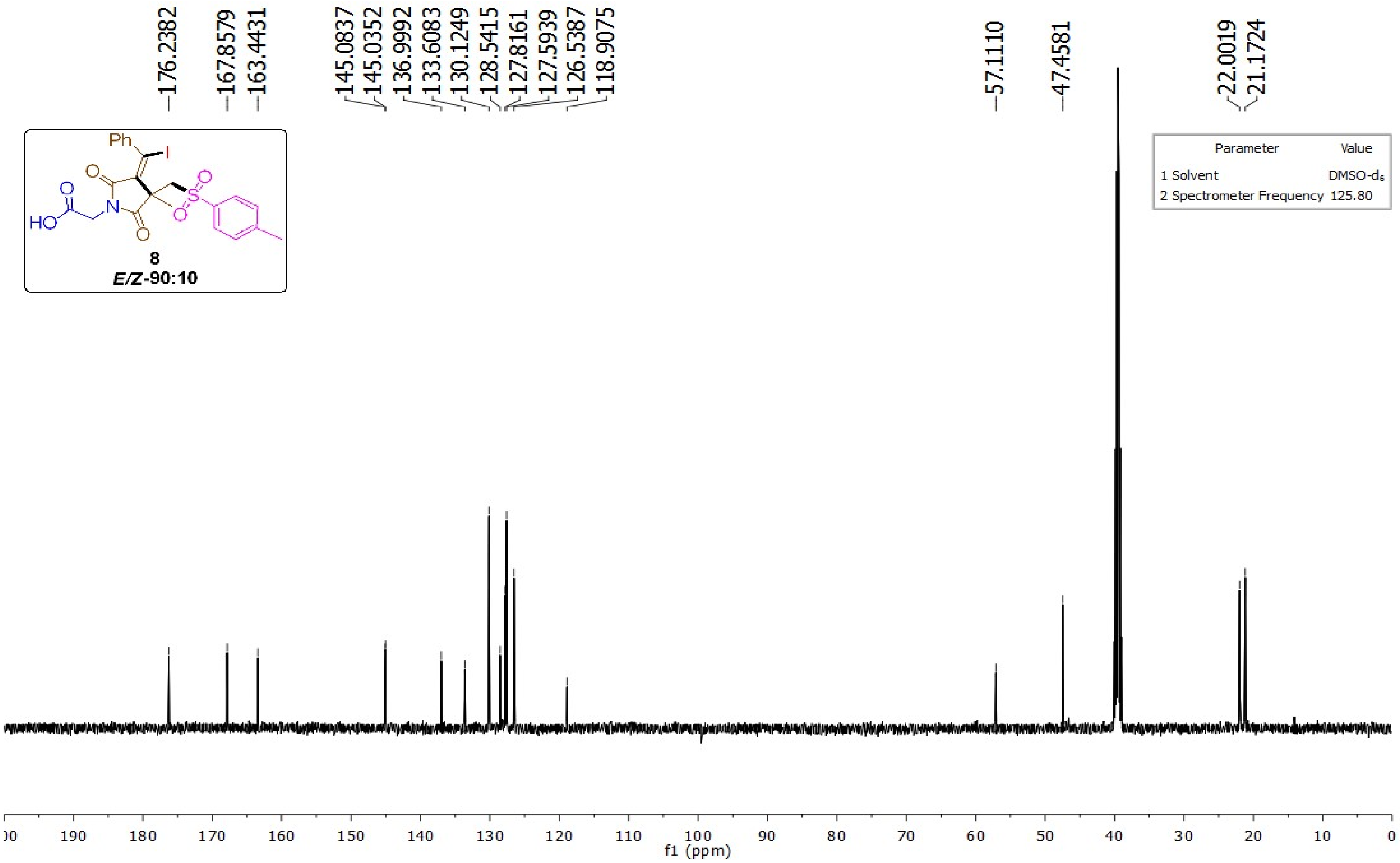

Figure S56. ^{13}C NMR spectra of 2-(4-(iodo(phenyl)methylene)-3-methyl-2,5-dioxo-3-(tosylmethyl) pyrrolidin-1-yl)acetic acid (**8**)

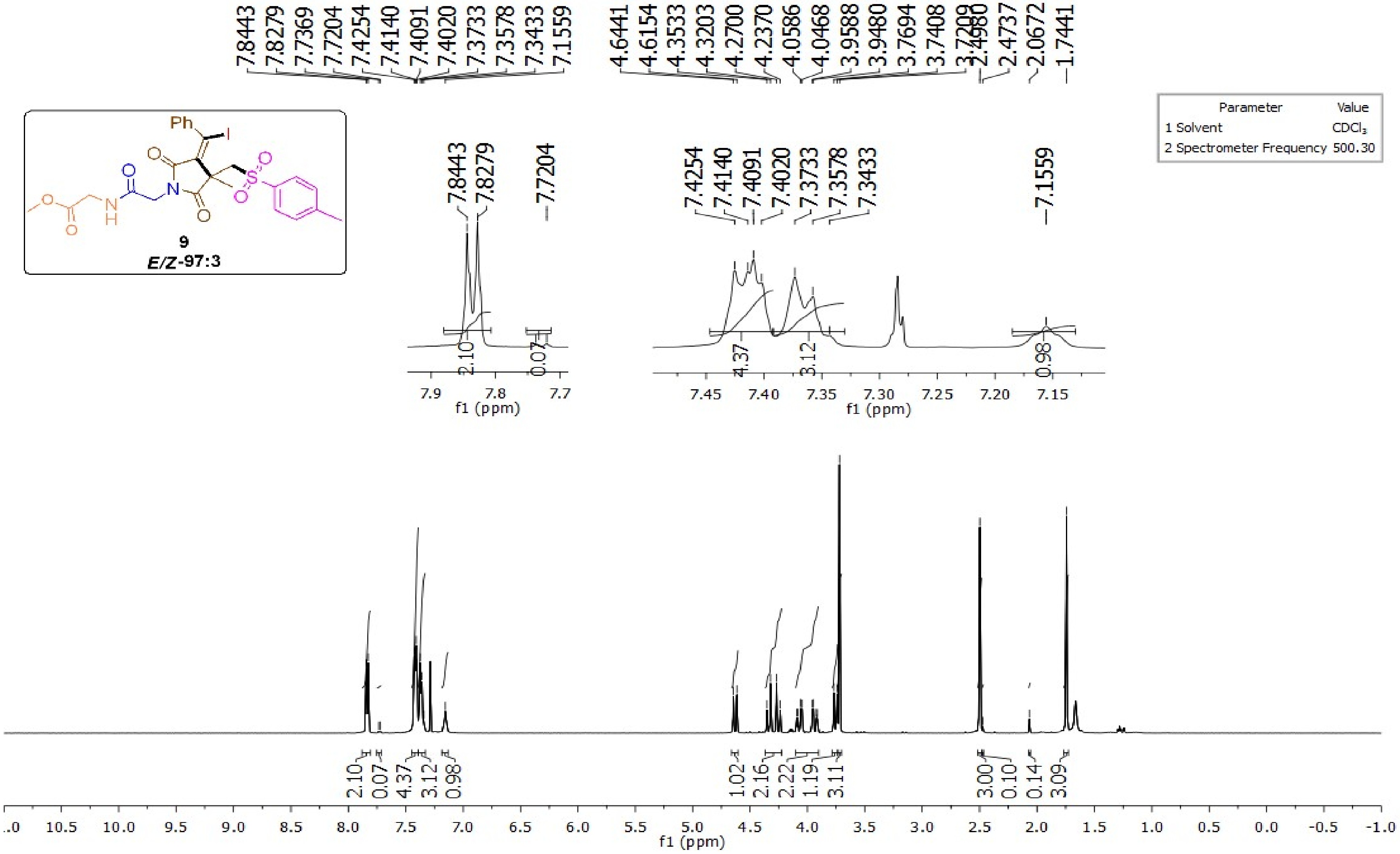

Figure S57. ^{1}H NMR spectra of Methyl (2-(4-(iodo(phenyl)methylene)-3-methyl-2,5-dioxo-3-(tosyl methyl)pyrrolidin-1-yl)acetyl)glycinate (**9**)

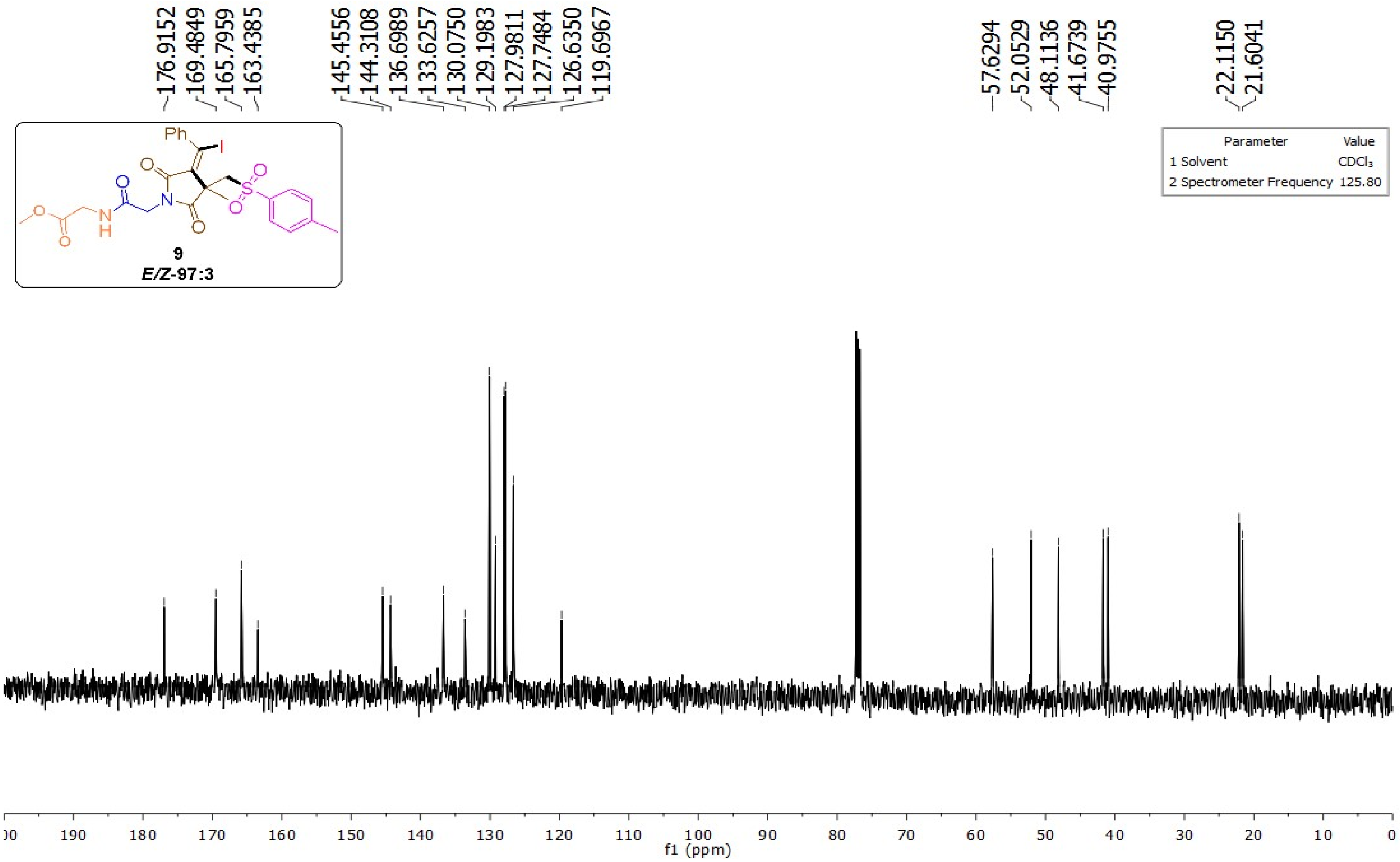

Figure S58. ^{13}C NMR spectra of Methyl (2-(4-(iodo(phenyl)methylene)-3-methyl-2,5-dioxo-3-(tosyl methyl)pyrrolidin-1-yl)acetyl)glycinate (**9**)

11. X-Ray Crystallographic Data of 5aa

Table S6 Crystal data and structure refinement for 5aa

Identification code	RMKSMP2_413_060524	
Chemical formula	$C_{23}H_{22}INO_6S$	
Formula weight	567.37 g/mol	
Temperature	300(2) K	
Wavelength	0.71073 Å	
Crystal size	0.178 x 0.260 x 0.278 mm	
Crystal habit	colorless block	
Crystal system	Monoclinic	
Space group	P 1 21/c 1	
Unit cell dimensions	a = 8.0223(8) Å	α = 90°
	b = 29.704(3) Å	β = 107.350(4)°
	c = 10.4353(11) Å	γ = 90°
Volume	2373.5(4) $Å^3$	
Z	4	
Density (calculated)	1.588 g/cm^3	
Absorption coefficient	1.475 mm^{-1}	
F(000)	1136	
Theta range for data collection	2.16 to 30.09°	
Index ranges	-11<=h<=11, -41<=k<=38, -13<=l<=14	
Reflections collected	42583	
Independent reflections	6952 [R(int) = 0.0551]	
Coverage of independent reflections	99.5%	
Absorption correction	Multi-Scan	
Max. and min. transmission	0.7790 and 0.6840	
Structure solution technique	direct methods	
Structure solution program	XT, VERSION 2018/2	
Refinement method	Full-matrix least-squares on F^2	
Refinement program	SHELXL-2019/1 (Sheldrick, 2019)	
Function minimized	$\Sigma w(F_o^2 - F_c^2)^2$	
Data / restraints / parameters	6952 / 0 / 292	
Goodness-of-fit on F^2	1.100	
Final R indices	3957 data; I>2σ(I)	R1 = 0.0767, wR2 = 0.1462
	all data	R1 = 0.1331, wR2 = 0.1686
Weighting scheme	$w=1/[\sigma^2(F_o^2)+13.9628P]$ where $P=(F_o^2+2F_c^2)/3$	
Largest diff. peak and hole	1.091 and -1.027 $eÅ^{-3}$	
R.M.S. deviation from mean	0.097 $eÅ^{-3}$	

Datablock rmksmp2_413_060524_0m_a - ellipsoid plot

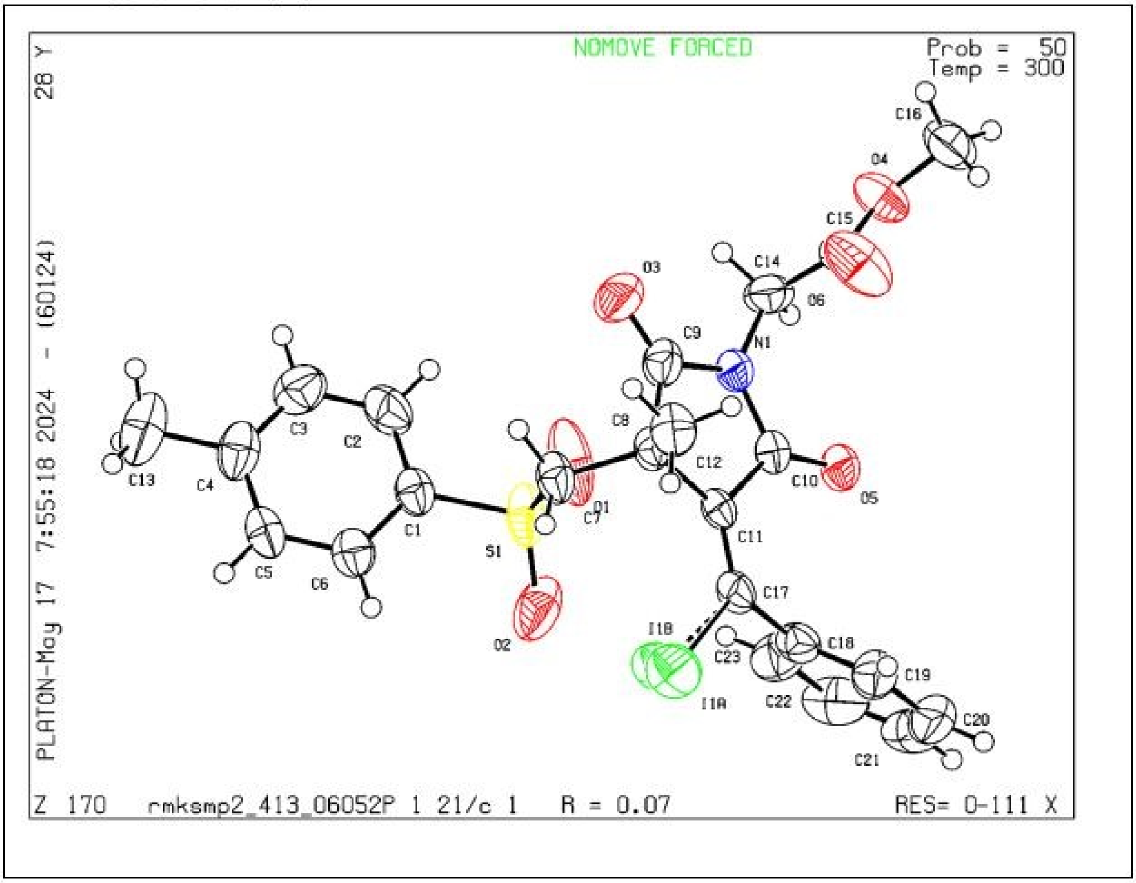

Figure S59. Single Crystal XRD image of compound Methyl 2-(4-(iodo(phenyl)methylene)-3-methyl-2,5-dioxo-3-(tosylmethyl)pyrrolidin-1-yl)acetate (**5aa**)

Crystal structure determination of 5aa

Crystal Data for $C_{23}H_{22}INO_6S$ (*M* =567.37 g/mol): monoclinic, space group P 1 21/c 1, *a* = 8.0223(8) Å, *b* = 29.704(3) Å, *c* = 10.4353(11) Å, *β* = 107.350(4)°, *V* = 2373.5(4) Å^3, *Z* = 4, *T* = 300(2) K, μ(Mo Kα) = 1.475 mm^{-1}, *Dcalc* = 1.588 g/cm^3 , 9881 reflections measured (4.924° < 2θ < 53.43°), 6952 unique [R(int) = 0.0551] which were used in all calculations. The final *R*1 was 0.0767(I > 2σ(I)) and *wR*2 was 0.1686 (all data).
